# Structural diversity of new mixed-ligand Ni(ii) complexes derived from ciprofloxacin and 2-methylbenzimidazole: synthesis, characterization, DFT insights, antimicrobial activity and DNA gyrase B (4DUH) docking

**DOI:** 10.1039/d6ra04805f

**Published:** 2026-09-29

**Authors:** Hany M. Abd El-Lateef, Huda Eltayeb, Mai M. Khalaf, Aly Abdou

**Affiliations:** a Department of Chemistry, College of Science, King Faisal University Al-Ahsa 31982 Saudi Arabia; b Department of Basic Sciences, College of Medicine, Princess Nourah bint Abdulrahman University P. O. Box 84428 Riyadh 11671 Saudi Arabia; c Department of Chemistry, Faculty of Science, Sohag University Sohag 82524 Egypt aly_abdou@science.sohag.edu.eg

## Abstract

A set of mixed ligand nickel(ii) complexes, namely, NiAC ([Ni(CIP)(MIM)(OAc)]), NiPE ([Ni(CIP)(MIM)(H_2_O)]ClO_4_), and NiCL([Ni(CIP)(MIM)(H_2_O)_2_Cl]), derived from the combination of ciprofloxacin (CIP) and 2-methylbenzimidazole (MIM), were synthesized in high yields (85–88%) and characterized by various physicochemical as well as computational techniques. The 1 : 1 : 1 stoichiometry was established by elemental analysis, whereas the non-electrolytic nature of the complexes was established by the molar conductance studies for NiAC (9.62 Ω^−1^ cm^2^ mol^−1^) and NiCL (10.85 Ω^−1^ cm^2^ mol^−1^), in comparison to the 1 : 1 electrolyte nature of the complex, NiPE (68.52 Ω^−1^ cm^2^ mol^−1^). The distorted tetrahedral geometry for the complexes, NiAC and NiPE, and octahedral geometry for the complex, NiCL, were established by the appearance of bands in the region 640, 610, 675 nm, respectively, in the electronic spectrum, as well as by the observed magnetic moments of 3.73, 3.68, 2.95 B.M., respectively. The bidentate coordination mode of CIP *via* the carboxylate and carbonyl groups, as well as the monodentate coordination mode of MIM *via* the nitrogen donor site were established by FT-IR analysis. Thermal analysis indicated good stability with decomposition above 225 °C, with NiO residues at the end of the reaction (∼11–13%). DFT results showed good reactivity for the complexes, especially NiAC, with the lowest HOMO–LUMO energy gap, *i.e.*, Δ*E* = 1.54 eV, the highest electrophilicity, *i.e.*, *ω* = 33.74 eV, and the highest softness, *i.e.*, *σ* = 0.65 eV^−1^. The Ni(ii) complexes exhibited appreciable antibacterial and antifungal activities under the tested conditions. NiAC showed a MIC of 40 µM, while the other complexes displayed MIC values of 40–50 µM. The complexes achieved activity indices of 74.29–93.33% against bacteria and 80.00–95.00% against fungi. Molecular docking was done with the active site of DNA gyrase B, *i.e.*, 4DUH, which showed good binding affinities with NiAC at −8.50 kcal mol^−1^ due to extensive hydrogen bonding and electrostatic interactions. The results revealed the synergistic effect of coordination that enhances biological activity and indicates that these complexes are promising antimicrobial agents.

## Abstract 2

New mixed ligand nickel(ii) complexes, NiAC ([Ni(CIP)(MIM)(OAc)]), NiPE ([Ni(CIP)(MIM)(H_2_O)]ClO_4_), and NiCL([Ni(CIP)(MIM)(H_2_O)_2_Cl]), derived from ciprofloxacin (CIP) and 2-methylbenzimidazole (MIM), were synthesized and characterized.

## Graphical abstract

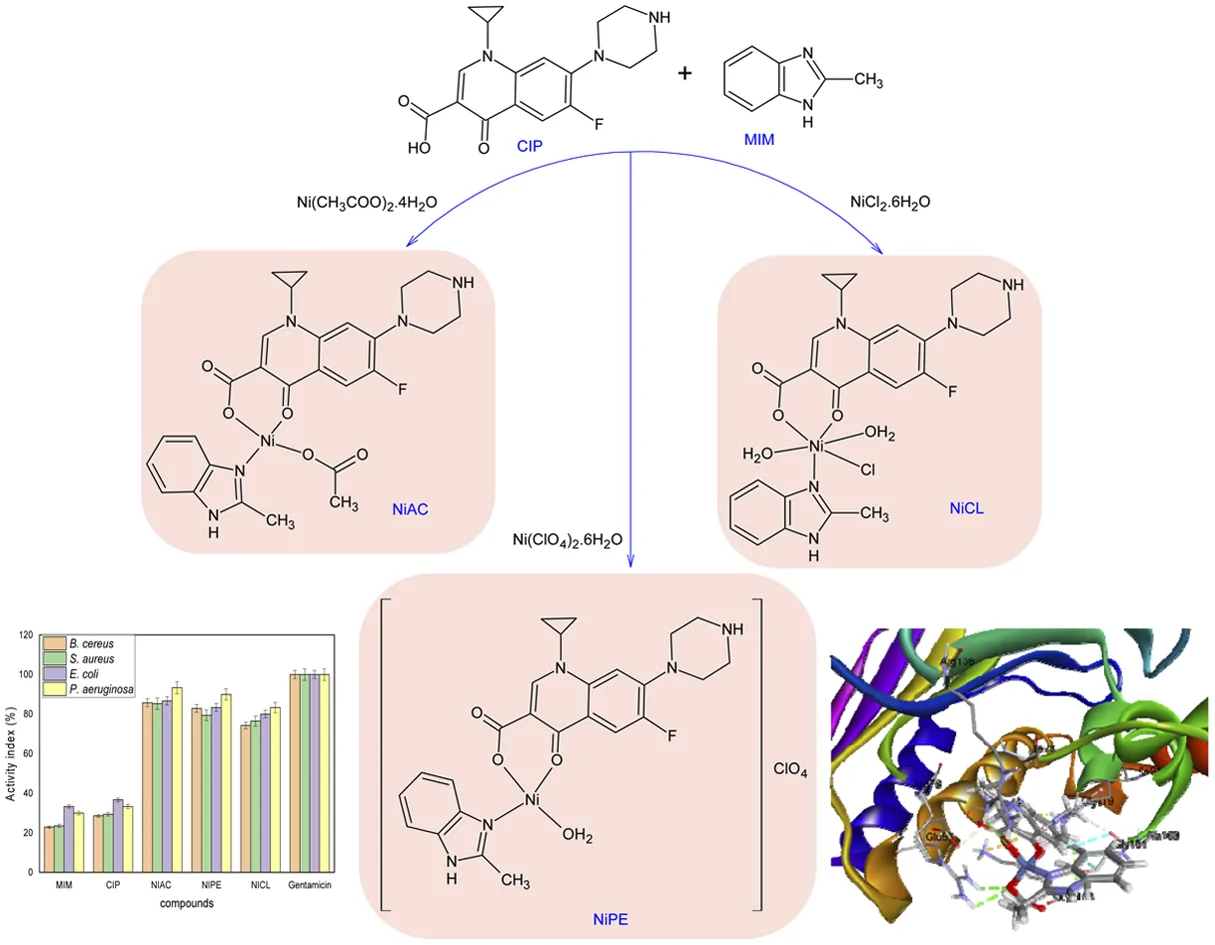

## 1. Introduction

Microbial pathogens, including bacteria and fungi, pose a growing and insidious menace to public health, environmental security, and economic security across the globe.^1^ In this context, bacteria like *E. coli*, *P. aeruginosa*, *B. cereus*, and *S. aureus* cause a variety of diseases ranging from food poisoning and hospital-acquired infections to life-threatening systemic illnesses in humans and animals.^2,3^ On the other hand, fungi like *A. flavus* and *C. albicans* also pose a critical health challenge, including opportunistic infections in immunocompromised subjects and crop losses due to mycotoxin contamination.^4–6^ Aside from the health-related consequences, the microorganisms also contribute to the degradation of water quality, soil fertility, and the economy in the form of healthcare expenses, reduced crop yield, and the cost of cleaning the environment.^7,8^ The pervasiveness of the microorganisms and the harm that they inflict on the environment underscore the need for the development of innovative approaches in the fight against microbes.^9,10^

The efficacy of these antimicrobial agents has been challenged by the alarming rate of antimicrobial resistance, a worldwide health crisis in which these antimicrobial agents have lost efficacy in treating infections.^10,11^ Bacteria and fungi have developed sophisticated ways to circumvent these antimicrobial agents, resulting in persistent infections, higher mortality rates, and higher healthcare costs. The need for novel antimicrobial compounds has opened avenues for exploring alternative therapeutic modalities.^12^ Transition metal complexes, especially those involving multiple ligands, have appeared as very promising compounds.^13^ These compounds have unique structural and electronic properties, which can overcome resistance problems, thus creating a new route to tackle resistant bacterial and fungal strains and preserve critical sectors in our environment and economy.

The advantages of mixed ligand complexes over their binary counterparts include reactivity and spectrum of biological activities.^14^ Unlike binary complexes that usually possess only one kind of ligand, mixed ligand complexes possess two or more kinds of ligands. This increases the complexity of their structures and consequently their physicochemical properties such as solubility, stability, and lipophilicity. In addition, the complexity of their structures increases their capacity for diverse modes of interactions with biological targets. This usually results in a synergistic effect.^15,16^ This usually results in enhanced antimicrobial activities and a better ability to evade resistance mechanisms than their binary counterparts.^17,18^

Nickel(ii) was selected as the metal center because of its versatile coordination chemistry and its ability to form complexes with diverse coordination geometries and electronic environments. This flexibility is particularly relevant to mixed-ligand systems, in which the nature of the coordinated ligands and ancillary groups can substantially influence the structural, electronic, and biological properties of the resulting complexes.^19–21^ In addition, Ni(ii) complexes containing benzimidazole-derived ligands have previously been investigated for their biological activities, supporting the use of nickel as a suitable metal center for exploring new bioactive coordination compounds.^22–24^ In the present study, Ni(ii) was specifically employed to investigate how the simultaneous coordination of ciprofloxacin and 2-methylbenzimidazole affects the structural features, electronic properties, and antimicrobial performance of the resulting mixed-ligand complexes.

Ciprofloxacin (CIP) is a well-known broad-spectrum fluoroquinolone antibiotic that has proven to be highly effective against a number of Gram-positive and Gram-negative bacteria.^25–28^ Its well-established mechanism of action has been the inhibition of the bacterial enzymes DNA gyrase and topoisomerase IV, which are essential for the replication and repair of the bacterial DNA. Despite the efficacy of ciprofloxacin, the occurrence of ciprofloxacin-resistant bacteria has highlighted the need for new derivatives or new formulations of the drug.^29–31^ Complexation of ciprofloxacin with transition metal ions has also shown promise in increasing the efficacy of the drug, broadening its spectrum of activity, and even overcoming the existing resistance, thereby giving a new lease of life to the drug.^25,32–34^

Methyl-benzimidazole (MIM) and its derivatives have been recognized for their various biological activities, such as antimicrobial, anthelmintic, and anticancer activities.^35–37^ The benzimidazole core is one of the privileged structures in medicinal chemistry, commonly functioning as a pharmacophore. The metal complexes derived from methyl-benzimidazole have been reported to display improved biological activities in comparison to the free ligand. The biological activities include improved stability, bioavailability, and antimicrobial activities.^38–41^ Considering the individual importance of both ciprofloxacin and methyl-benzimidazole, it is evident that the synergistic effect of both ligands in one single transition metal complex is an interesting strategy. The strategy is based on the fact that it can result in the development of a new compound with improved and broader-range antimicrobial activities, thus meeting the urgent need for new drugs. Despite extensive studies on ciprofloxacin and benzimidazole-based metal complexes, the rational design of mixed-ligand Ni(ii) systems integrating both pharmacophores within a single coordination environment remains underexplored. The present work introduces a new class of structurally diverse Ni(ii) complexes with tunable geometries, enabling a direct investigation of the synergistic effect of dual ligands on electronic structure and biological activity. Furthermore, the integration of DFT-derived reactivity descriptors with experimental antimicrobial and molecular docking analyses provides a comprehensive structure–activity relationship that has not been systematically reported for such systems.

Therefore, the main aim of this research is to design and extensively investigate the structural, electronic, and thermal characteristics of a series of novel mixed ligand nickel(ii) complexes incorporating the fluoroquinolone-type antibiotic ciprofloxacin (CIP) and 2-methylbenzimidazole (MIM) ligand using both experimental physicochemical studies and DFT computational modeling. Additionally, the research seeks to explore the cooperative impact of metal coordination on the biological activity of the parent ligands by examining their antimicrobial efficacy against various pathogenic strains. A significant aspect of this research is the use of molecular docking studies targeting the DNA gyrase B (4DUH) target to gain insight into the mechanism of ligand–receptor interactions, thereby validating the potential of the designed complexes as the most promising next-generation antimicrobial agents.

## 2. Methodology

### 2.1. Materials and instruments

All the materials and instrumental techniques used in this study have been mentioned in the SI with details, see S1 Materials and instruments.

### 2.2. Synthesis of the 2-methylbenzimidazole ligand (MIM)

The ligand 2-methylbenzimidazole (MIM) was synthesized earlier by us,^41^ details of the synthesis have been mentioned in the SI, see: S2 Synthesis of 2-methyl-benzimidazole (MIM), Scheme S1 and Fig. S1.

### 2.3. Synthesis of the NiAC, NiPE, and NiCL complexes

The NiAC, NiPE, and NiCL complexes were prepared using a slightly modified version of a previously published procedure.^42^ In this case, an aqueous solution of a metal salt (20 mL) was prepared using nickel(ii) chloride hexahydrate (NiCl_2_·6H_2_O, 2.0 mmol, 0.474 g), nickel(ii) acetate tetrahydrate (Ni(CH_3_COO)_2_·4H_2_O, 2.0 mmol, 0.496 g), and nickel(ii) perchlorate hexahydrate (Ni(ClO_4_)2·6H_2_O, 2.0 mmol, 0.730 g). Subsequently, this solution was slowly added to a preheated solution of equimolar amounts of a 2-methylbenzimidazole ligand (MIM, 2.0 mmol, 0.264 g) and a ciprofloxacin ligand (CIP, 2.0 mmol, 0.662 g) in ethanol (20 mL) at 60 °C. After this process, refluxing at 80 °C and constant stirring for 5 h ensured complete reaction. Subsequent filtering of the powder formed upon cooling gave rise to the formation of the complexes. Subsequent washing of these products using ethanol ensured that any traces of unreacted substances were removed. Finally, air-drying of these products gave rise to the formation of the final products. The entire process of making these complexes is shown schematically in Scheme 1.

**Scheme 1:**
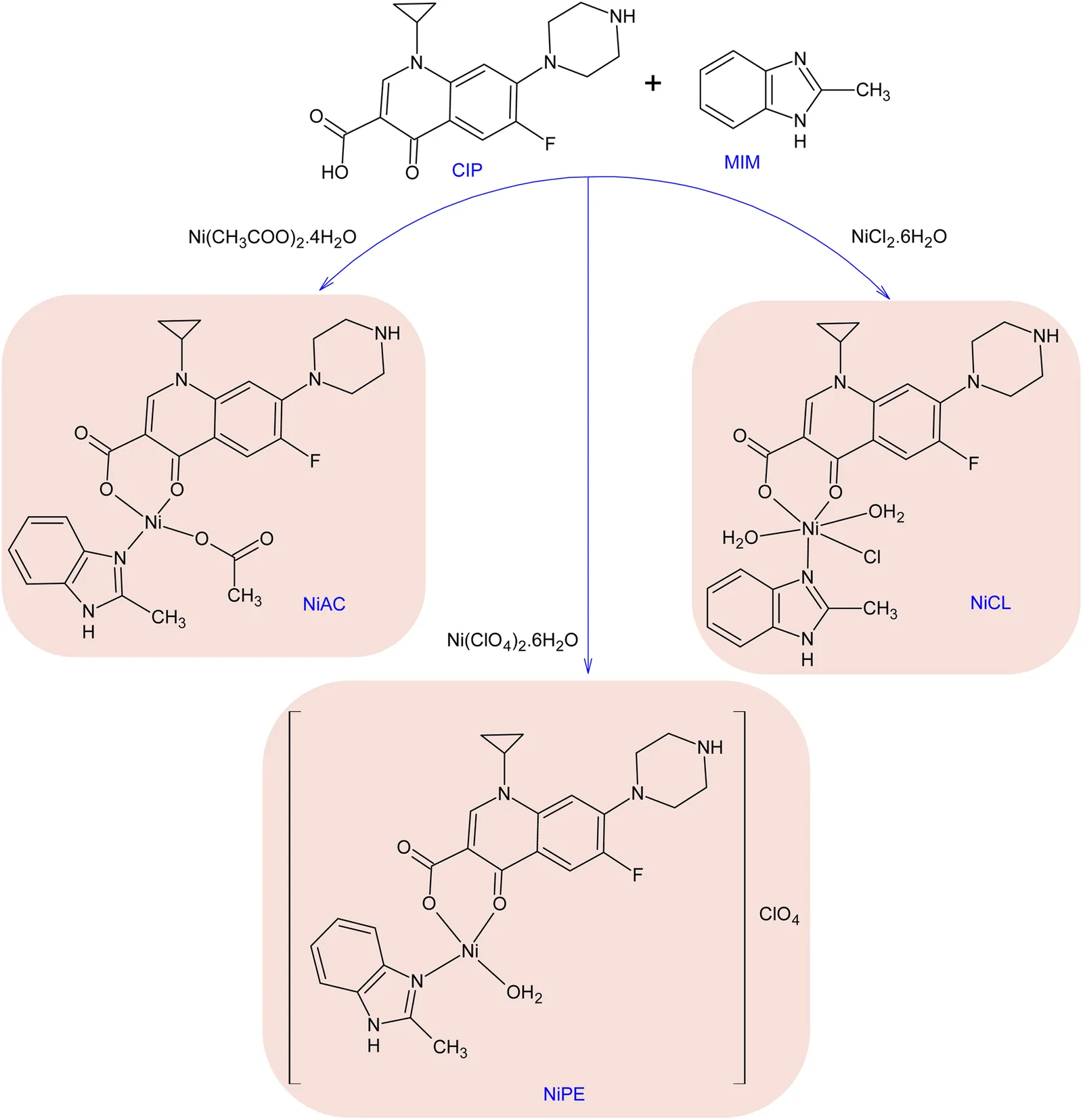
Synthesis of the NIAC, NIPE, and NICL complexes.

### 2.4. DFT calculations

The information about the DFT computation^43–48^ of the MIM, and CIP ligands, and their NiAC, NiPE, and NiCL complexes is presented in the SI Section; see S3 DFT Calculations.

### 2.5. Antimicrobial testing *in vitro*

The *in vitro* screening of the antimicrobial activity of the MIM and CIP ligands, and their NiAC, NiPE, and NiCL complexes, *i.e.*, antibacterial and antifungal activity^49–52^ is presented in the SI Section; see S4 Antimicrobial testing *in vitro*. The antibacterial activities of the investigated compounds were evaluated against *Bacillus cereus*, *Staphylococcus aureus*, *Escherichia coli*, and *Pseudomonas aeruginosa* using the disk diffusion method. The tested compounds were dissolved in DMSO at a concentration of 1.5 mg mL^−1^. The antifungal activities of the investigated compounds were evaluated against *Candida albicans and Aspergillus flavus* using the disk diffusion method. The compounds were dissolved in DMSO at a concentration of 1.5 mg mL^−1^. Following incubation, the diameters of the inhibition zones were measured in millimeters. DMSO was used as the negative control.All measurements were performed in triplicate and expressed as mean ± SD (*n* = 3). The activity index (AI, %) was calculated using Gentamicin as the reference antibacterial drug and Clotrimazole as the reference antifungal drug. The activity index was calculated as the inhibition-zone diameter of the tested compound divided by that of the corresponding standard drug, multiplied by 100.

### 2.6. Molecular docking study

The molecular docking studies for the tested compounds were performed using AutoDock Vina algorithm.^53^ The docking process was intended to examine the interactions between the tested compounds and the DNA gyrase B target protein (PDB ID: 4DUH).^54^ The high-resolution crystal structure of the DNA gyrase B protein (≈1.5 Å) was obtained from the Protein Data Bank (https://www.rcsb.org/structure/4UDH). Before the docking process, the protein structure was correctly prepared by removing the crystal water molecules, co-crystallized ligands, and hetero atoms, followed by the addition of polar hydrogen atoms and Kollman charges. The ligand structures were generated and optimized using the proper preparation protocol.^55^ A precise grid was set for the docking process to cover the target protein's active site completely by setting the *X*, *Y*, *Z* coordinates to 20.879, 12.334, 23.800, respectively. In addition, the docking process's validity was tested by evaluating the docking performance using the native ligand, 4-([4′-methyl-2'-(propanoylamino)-4,5′-bi-1,3-thiazol-2-yl]amino) benzoic acid, co-crystallized ligand with 4DUH.

## 3. Results and discussions

### 3.1. Characterization of the MIM ligand

The MIM ligand was fully characterized based on its ^1^H-NMR, IR, and UV-vis., Fig. S1. The characterization was fully discussed in the SI data, S5 Structure elucidation of the MIM ligand.

### 3.2. Characterization of the NiAC, NiPE, and NiCL complexes

The powder complexes of the synthesized mixed ligand nickel(ii) complexes NIAC, NiPE and NiCL were obtained with high yields 87%, 88%, and 85% respectively. The melting points of these coordination compounds (Table S1) are >300 °C, suggesting improved thermal stability relative to their corresponding free ligands CIP and MIM.

An element presented in the NIAC, NIPE, and NICL complexes, *i.e.*, carbon (C), hydrogen (H), nitrogen (N), and nickel (Ni), was carried out by elemental analysis to confirm the proposed formula of the complexes, Table S2. It was observed that the experimental percentage values obtained for the elements present in the complexes were in excellent agreement with the calculated percentage values. For example, in the case of the NIAC complex containing C_27_H_28_FN_3_NiOS, the experimental percentage values obtained were found to be C = 55.08% (calc. 55.89%), H = 4.37% (calc. 4.86%), N = 12.34% (calc. 12.07%), and Ni = 10.33% (calc. 10.12%). Similarly, in the case of the NIPE complex containing C_25_H_27_FN_3_NiO_8_Cl, the experimental percentage values obtained were found to be C = 47.24% (calc. 47.02%), H = 4.51% (calc. 4.26%), N = 10.72% (calc. 10.87%), and in the case of NICL containing C_25_H_29_C1FN_5_NIO_5_, the actual percentages obtained were found to be C: 50.40% (calc. 50.66%), H: 4.64% (calc. 4.93%), N: 11.55% (calc. 11.82%), and Ni: 10.21% (calc. 9.90%). The consistency in the actual and calculated percentage values serves as an indicator of the 1 : 1 : 1 ratio between the nickel ion and the ciprofloxacin (CIP) and 2-methyl-benzimidazole (MIM) ligands.

Molar conductivity measurements carried out in ethanol solution with a concentration of 10 mM clearly indicated the nature of the complexes, Table S3. The lower conductivity of the two complexes, NIAC (9.62 Q^−1^cm^2^ mol^−1^) and NICL (10.85 Q^−1^cm^2^ mol^−1^), indicate that they are non-electrolytes. This further indicates that the acetate and chloride ions are coordinating in such a way that they are part of the coordination sphere. In contrast, the conductivity value of the NIPE complex is much higher at 68.52 Q^−1^cm^2^ mol^−1^, indicating that it is a 1 : 1 electrolyte. This clearly indicates that the complex has a counter anion outside the coordination sphere. This is clearly indicated in the formula of the complex: [Ni(CIP)(MIM)(H_2_O)]C1O_4_.

The electronic (UV-vis.) spectra of NIAC, NIPE, and NICL complexes, in ethanol, gave valuable information about d–d transitions and coordination geometry, Fig. S2 and Table S4. The bands in the ultraviolet region for free ligands, *i.e.*, CIP: 324, 365 nm, and MIM: 236, 292 nm, were shifted in their respective complexes. NIAC and NIPE complexes showed characteristic bands at 640 nm (15 625.00 cm^−1^) and 610 nm (16 393.44 cm^−1^), respectively. These bands are due to ^3^T_1_(F) → ^3^T_1_(P) transitions, which are very characteristic of tetrahedral geometry. On the other hand, in NICL, a distinct band was observed at 675 nm (14 814.81 cm^−1^), corresponding to a transition of ^3^A_2g_ → ^3^T_1g_(F), which is very characteristic of octahedral geometry.

Magnetic susceptibility measurements were conducted to understand the electronic configuration and geometry of coordination of nickel(ii) complexes, Table S5. For both NiAC and NIPE, magnetic moments of 3.73 B.M. and 3.68 B.M. were observed, respectively. These magnetic moment values indicate that a Ni(ii) ion, *d*^8^ configuration, has two unpaired electrons, thus indicating a distorted tetrahedral geometry. However, a magnetic moment of 2.95 B.M. was observed for the NICL complex, thus indicating that it has an octahedral geometry, t_2g_^6^e_g_^2^, of a nickel(ii) ion. Therefore, it is evident that in all three nickel(ii) complexes, the nickel(ii) ion retains its paramagnetic character. Whereas the significant variation in magnetic moment values is a clear indicator of a change in geometry from a four-coordinate distorted tetrahedral geometry of NIAC and NIPE to a six-coordinate distorted octahedral geometry of NiCL.

The stoichiometry of the NIAC, NIPE, and NICL complexes were further explored using the spectrophotometric Job's method of continuous variation, Fig. S3. This involved the variation of the molar ratios of the nickel(ii) ion and the CIP and MIM ligands while maintaining a constant total concentration. Job's plot results consistently indicated the formation of the [Ni(CIP)(MIM)] unit with a 1 : 1 : 1 molar ratio for all three complexes. This stoichiometry is in perfect agreement with the proposed molecular formulas and is strongly supported by the results obtained from the elemental analysis and mass spectrometric studies.

The coordination mode of ciprofloxacin (CIP) was assigned based on the combined FT-IR, Fig. S4 and Table S6, and DFT results. The carbonyl stretching band of free CIP at 1628 cm^−1^ shifted to 1655, 1648, and 1650 cm^−1^ for NiAC, NiPE, and NiCL, respectively. In addition, the asymmetric carboxylate stretching vibration shifted from 1702 cm^−1^ in free CIP to 1611, 1617, and 1609 cm^−1^, respectively, accompanied by symmetric carboxylate bands at 1382, 1380, and 1388 cm^−1^. The corresponding Δ*ν* values were 229, 237, and 221 cm^−1^. These spectral changes, together with the appearance of M–O bands at 572, 568, and 570 cm^−1^, support the involvement of the carbonyl and carboxylate oxygen atoms of CIP in Ni(ii) coordination. No additional spectroscopic feature was observed that would specifically support coordination through the nitrogen atom of the CIP moiety. The M–N bands observed at 504, 508, and 502 cm^−1^ are assigned to the nitrogen donor of 2-methylbenzimidazole, consistent with the changes observed in its C=N stretching vibration. The DFT-optimized structures further support the proposed donor arrangement, showing Ni–O interactions involving the CIP oxygen atoms and a Ni–N interaction associated with the MIM ligand. Therefore, the experimental and theoretical results consistently support an O,O-bidentate coordination mode of CIP through the carboxylate and quinolone carbonyl oxygen atoms, while the nitrogen atom of CIP is not involved in the proposed primary coordination sphere. Nevertheless, because FT-IR spectroscopy is not by itself sufficient to categorically exclude all alternative coordination modes, this assignment is presented as being supported by the combined spectroscopic, analytical, and DFT evidence.

Mass spectrometry was used to confirm the molecular weights and formulae for the NIAC, and NICL complexes, Fig. S5. The data for each complex showed peaks for the molecular ions (M^+^) at *m*/*z* 583.42 for NiAC, and *m*/*z* 591.57 for NiCL. These results compare favorably with the calculated molecular weights for each of these compounds, namely, C_27_H_28_FN_3_NiOS (calc. 580.23), and C_25_H_29_C1FN_5_NIO_5_ (calc. 592.67), respectively. It is noteworthy that the molecular ion peak observed in the mass spectrum of the NiAC complex does not exactly match the calculated molecular weight derived from elemental analysis. This discrepancy can be attributed to the inherent fragmentation behavior of coordination compounds under ionization conditions. In particular, the coordinated acetate group is relatively labile and can be readily eliminated as CH_3_COO^−^ or CH_3_COOH, resulting in fragment ions such as [M–OAc]^+^. Additionally, partial fragmentation of the ciprofloxacin ligand, including decarboxylation (loss of CO_2_) or cleavage of the piperazine moiety, may further contribute to lower *m*/*z* signals. Moreover, the molecular ion peak for Ni(ii) complexes is often weak, with the base peak corresponding to a more stable fragment ion. Therefore, the observed mass spectral peaks are consistent with the proposed structure when typical fragmentation pathways are considered, while the elemental analysis provides definitive confirmation of the molecular composition. It is noteworthy that the mass spectrum of the NiCL complex does not exhibit a well-resolved chlorine isotopic pattern (^35^Cl/^37^Cl ≈ 3 : 1). This can be attributed to the use of electron ionization (EI), a hard ionization technique that promotes extensive fragmentation of the complex. Under these conditions, preferential cleavage of the Ni–Cl bond occurs, resulting in the formation of dominant fragment ions that do not retain chlorine. Consequently, ligand-based fragments derived from ciprofloxacin and 2-methylbenzimidazole dominates the spectrum, masking any low-intensity chlorine-containing ions. Additionally, the presence of multiple nickel isotopes contributes to peak broadening and further obscures the expected isotopic distribution.

Mass spectrum of NiPE complex showed ion at *m*/*z* = 469.38, that is interpreted as a fragment rather than the intact molecular ion. NiPE is proposed as [Ni(CIP)(MIM)(H_2_O)]ClO_4_ with formula C_25_H_27_FN_5_NiO_8_Cl (calc. 638.655), in which the perchlorate ion remains outside the coordination sphere, consistent with the relatively high molar conductivity of 68.52 Ω^−1^ cm^2^ mol^−1^ and its 1 : 1 electrolyte behavior. Under the ionization conditions, fragmentation can involve cleavage of the outer-sphere perchlorate component, decarboxylation of the ciprofloxacin moiety with loss of CO_2_, and elimination of H_2_O, producing a substantially lower *m*/*z* nickel-containing fragment. Accordingly, the peak at *m*/*z* = 469.38 may be regarded as a diagnostic fragment ion rather than the molecular ion of the intact complex. This interpretation is consistent with the known propensity of coordination compounds to undergo ligand and counter ion loss during mass spectrometric ionization. Moreover, the elemental analysis supports the formula C_25_H_27_FN_5_NiO_8_Cl (calc. 638.655) for the bulk composition and 1 : 1 : 1 Ni : CIP : MIM stoichiometry.

The thermal degradation behavior of the NIAC, NIPE, and NICL complexes has been thoroughly examined by employing thermogravimetric analysis (TGA) in the temperature range from 25–800 °C, Table 1 and Fig. S6. The thermogravimetric analysis (TGA/DTG) of NiAC, NiPE, and NiCL was performed to evaluate their thermal stability and decomposition behavior. The complexes exhibited thermal decomposition above 225 °C, proceeding through successive mass-loss steps. The observed mass losses were compared with calculated values to provide a tentative stoichiometric interpretation of the corresponding decomposition stages. It should be emphasized that the compositions listed in, Table 1, and Fig. S6, such as C_14_H_24_N_2_O_2_ and C_13_H_4_FN_3_O_2_, they are approximate compositions used to rationalize the experimentally observed mass losses. The thermal degradation of the coordinated organic ligands is expected to involve simultaneous and/or consecutive bond cleavage and fragmentation processes; therefore, the proposed assignments should be regarded as tentative possibilities rather than definitive identification of individual decomposition products.

**Table 1:** Thermal degradation data of the NiAC, NiPE, and NiCL

Compounds	TG (°C)	Mass loss (%)			Residue (%)	
		DTG (°C)	Found (calculated)	Assignment	Found (calculated)	Assignment
NiAC	280–475	380	43.155 (43.492)	C_14_H_24_N_2_O_2_	56.721 (56.509)	C_13_H_4_FN_3_NiO_3_
	475–685	550	43.427 (43.636)	C_13_H_4_FN_3_O_2_	12.957 (12.873)	NiO
NiPE	225–420	330	48.815 (48.661)	1H_2_O + C_12_H_21_ClN_2_O_4_	51.139 (51.339)	C_13_H_4_FN_3_NiO_3_
	420–680	545	39.804 (39.644)	C_13_H_4_FN_3_O_2_	11.504 (11.695)	NiO
NiCL	230–450	335	38.442 (38.696)	2H_2_O + C_12_H_21_N_2_O	61.483 (61.305)	C_13_H_4_ClFN_3_NiO_3_
	450–685	575	48.532 (48.702)	C_13_H_4_ClFN_3_O_2_	12.443 (12.603)	NiO

For NiAC, the first major mass-loss step occurs over 280–475 °C with an experimental mass loss of 43.155%, followed by a second step between 475 and 685 °C with a mass loss of 43.427%. For NiPE and NiCL, analogous multistep decomposition processes are observed. The close agreement between the experimental and calculated mass losses supports the proposed stoichiometric interpretation of these thermal events, but does not establish the exact molecular identity of the evolved decomposition products. The final residues of NiAC, NiPE, and NiCL are 12.957%, 11.504%, and 12.443%, respectively, which are close to the calculated values for NiO (12.873%, 11.695%, and 12.603%, respectively). Thus, the TGA results support the proposed thermal decomposition pathways and formation of NiO as the final inorganic residue, while the identities of the intermediate organic decomposition products remain tentative.

The pH stability curves of NiAC, NiPE, and NiCL in aqueous alcoholic media can provide information on the concentrations of protons on the bonding between the ligands and the metal in aqueous media, Fig. 1. By analyzing the absorbance spectra, it can be easily deduced that NiAC, NiPE, and NiCL are highly dependent on the pH value of the solution. In strongly acidic media with a pH value between 2–4, there is a reduction in the level of absorbance of the NiAC, NiPE, and NiCL complexes. This can be easily explained in terms of the instability of the bonding between the ligands and the metal in strongly acidic media. This is because the ligand's carrier atoms get protonated in such media. As the pH value of the solution approaches the neutral region of the pH value scale, there is an increase in the level of absorbance of the NiAC, NiPE, and NiCL complexes. This level of absorbance peaks at a plateau in the pH value range of 6–8. This is the most stable point of the bonding between the ligands and the metal. As the pH value of the solution is further increased and the solution becomes more basic with a pH value higher than 8–10, there is a reduction in the level of absorbance of the NiAC, NiPE, and NiCL complexes.

**Fig. 1:**
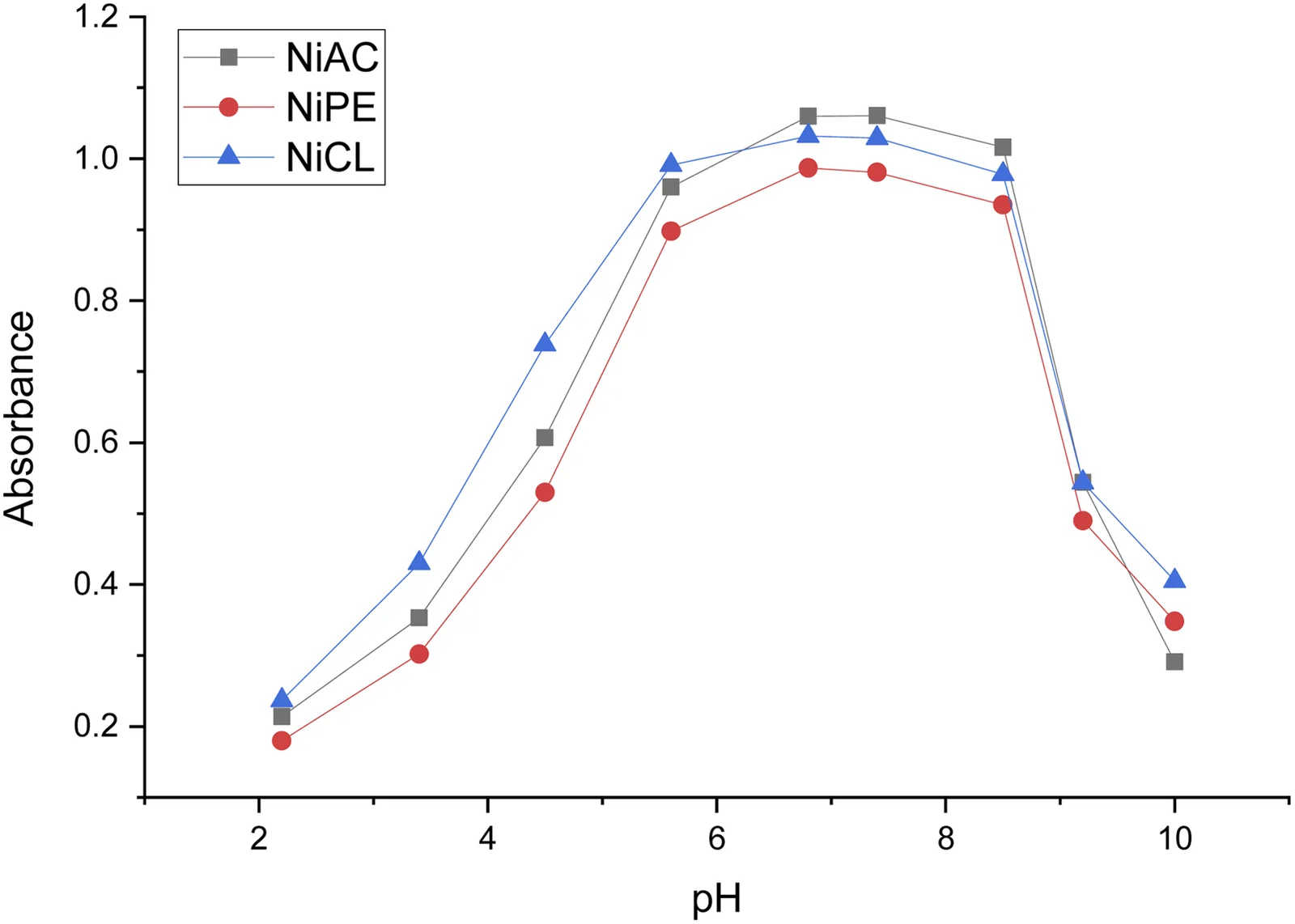
pH stability curve of the NiAC, NiPE, and NiCL complexes, the absorbance was measured at the absorption maxima (*λ*_max_) for each Ni(ii) complex (at 640 nm, 610 nm, and 675 nm for NIAC, NIPE and NICL complexes, respectively).

In conclusion, the physico-chemical characterization of the NIAC, NIPE, and NICL complexes has consistently confirmed the molecular formula and coordination geometries of the complexes as proposed in the Scheme 1 above. The NIAC and NIPE complexes were found to have distorted tetrahedral geometries, while the NICL complex was found to have a distorted octahedral geometry. The elemental analysis, molar conductance, magnetic measurements, and spectroscopic studies (UV-vis, IR, and Mass) are consistent in confirming the 1 : 1 : 1 stoichiometry of the CIP and MIM ligand coordination in the nickel(ii) complexes. Furthermore, the thermal analysis confirms the composition of the nickel(ii) complexes, including the coordinated water molecules, and confirms the stability of the nickel(ii) complexes.

### 3.3. DFT calculations

Both NIAC, and NIPE depict the tetrahedral geometry for the Ni(ii) ion, Fig. 2, consistent with the experimental observations depicted in Scheme 1. However, the NICL depicts the octahedral geometry for the Ni(ii) ion, Fig. 2, consistent with the experimental observations depicted in Scheme 1. The optimized 3D structure of the MIM and CIP ligands were depicted in Fig. S7. This is supported by the calculated bond angles, which are close to the ideal octahedral geometry, as presented in Table S7.

**Fig. 2:**
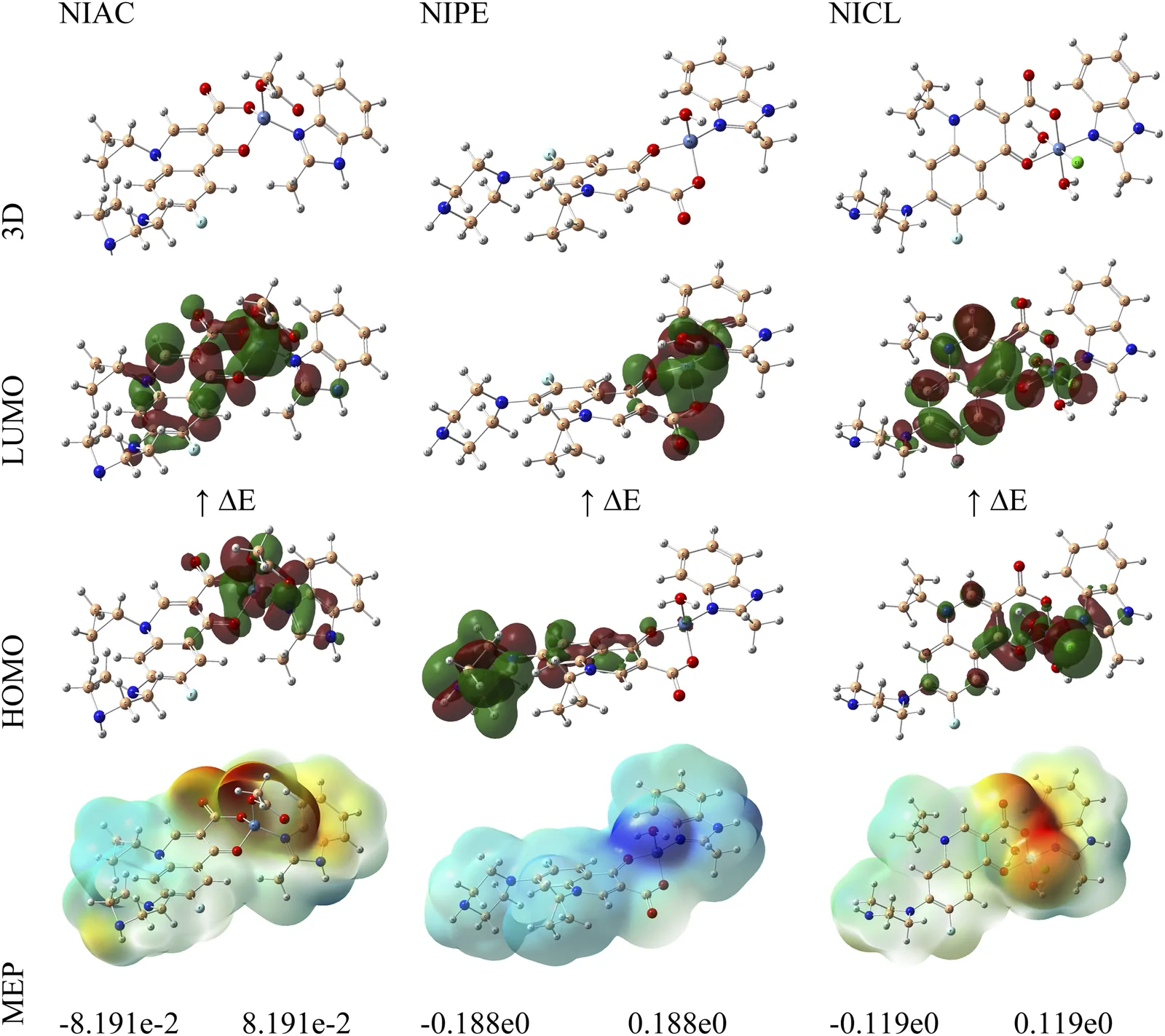
3D, HOMO, LUMO, and MEP of NIAC, NIPE, and NICL complexes.

The Frontier molecular orbitals are the focal point for the action in the molecule. The Highest Occupied Molecular Orbital (HOMO) and the Lowest Unfilled Molecular Orbital (LUMO) control the reactivity as well as the resistance to change in the molecule. The energies for the compounds described in Table 2, as well as the distribution of the orbitals as described in Fig. 2, indicate the positions of the positive and negative areas. In the NIAC, NIPE, and NICL complexes, the HOMO is mainly localized on the ligand skeleton, especially the nitrogen atoms as well as the aromatics, which emphasizes the role the ligand plays in this respect. On the contrary, LUMO is distributed throughout the complex, especially the metal in varying degrees. Moreover, we have computed the values for HOMO, LUMO, the energy gap (Δ*E*), the electronic chemical potential (*µ*), the chemical hardness (*η*), the electrophilicity (*ω*), as well as the softness (*σ*), which are described in Table 2.

**Table 2:** Calculated DFT parameters (Δ*E* = energy gap, IP = ionization potential, EA = electron affinity, *χ* = electronegativity, *µ* = electronic chemical potential, *η* = chemical hardness, *ω* = electrophilicity index, *σ* = softness, Nu = nucleophilicity index)

Compounds	HOMO	LUMO	Δ*E*	*I*	*A*	*χ*	CP	*η*	*σ*	*ω*	Nu
MIM	−6.54	−0.95	5.59	6.54	0.95	3.74	−3.74	2.79	0.18	2.51	0.40
CIP	−6.27	−2.52	3.74	6.27	2.52	4.39	−4.39	1.87	0.27	5.16	0.19
NIAC	−7.98	−6.44	1.54	7.98	6.44	7.21	−7.21	0.77	0.65	33.74	0.03
NIPE	−6.46	−4.79	1.67	6.46	4.79	5.63	−5.63	0.84	0.60	18.94	0.05
NICL	−5.18	−3.23	1.95	5.18	3.23	4.20	−4.20	0.97	0.51	9.07	0.11

The energy level of the Highest Occupied Molecular Orbital (HOMO) is an important factor in assessing the electron-donating ability and nucleophilicity of molecules. The greater the energy level of the HOMO, the greater the electron-donating ability is. The energy level of the HOMO for the compounds under study is as follows: MIM (−6.54 eV), CIP (−6.27 eV), NIAC (−7.98 eV), NIPE (−6.46 eV), and NICL (−5.18 eV). The highest energy level is for NICL, making it the best electron donor. On the contrary, NIAC has the lowest energy level for the HOMO, making it the least electron donor. The energy level of the Lowest Unoccupied Molecular Orbital (LUMO) is an indicator of electron-accepting ability and electrophilicity. The lower energy level for LUMO indicates greater electron-accepting ability, making it an electrophile. The LUMO values of all compounds are: MIM −0.95 eV, CIP −2.52 eV, NIAC −6.44 eV, NIPE −4.79 eV, and NICL −3.23 eV. NIAC has the lowest LUMO value, thus it is the best electron acceptor and electrophile. MIM, on the other hand, has the highest LUMO value, thus it is the weakest electrophile. The energy gap, Δ*E*, is a significant parameter used to describe a molecule's stability and reactivity. A lower value of Δ*E* indicates higher reactivity. The calculated values of Δ*E* are: MIM = 5.59 eV, CIP = 3.74 eV, NIAC = 1.54 eV, NIPE = 1.67 eV, and NICL = 1.95 eV. NIAC has the lowest value of Δ*E*, implying that it is the most reactive compound, followed by NIPE and NICL. A lower value of Δ*E* is usually associated with higher biological activity, since a more reactive compound can participate in a required interaction in a biological system.

The calculated HOMO–LUMO energy gap (Δ*E*) provides a qualitative measure of the electronic excitation energy and can be correlated with the observed UV-vis absorption behavior. Complexes exhibiting smaller Δ*E* values tend to show absorption bands at longer wavelengths (bathochromic shift), consistent with reduced excitation energy requirements. However, it should be noted that the experimental spectra of Ni(ii) complexes involve both d–d transitions and charge transfer transitions (LMCT/MLCT), whereas the computed HOMO–LUMO gap primarily reflects frontier orbital transitions. Therefore, the correlation between Δ*E* and *λ*_max_ is qualitative rather than quantitative. Nevertheless, the DFT results support the observed spectral trends and provide insight into the electronic structure and coordination environment of the Ni(ii) complexes.

Electronegativity (*χ*) refers to the inherent ability of a molecule to attract electrons in a chemical bond. Increasing electronegativity implies that the molecule has a stronger ability to attract electrons in the bond. The electronegativity values of the compounds are as follows: MIM (3.74 eV), CIP (4.39 eV), NIAC (7.21 eV), NIPE (5.63 eV), and NICL (4.20 eV). NIAC has the highest electronegativity value, making it the most electron-attracting compound. In biological targets, highly electronegative compounds may specifically bind to regions that are electron rich. Electronic chemical potential (*µ*), which is the negative of electronegativity, is defined as the escaping tendency of electrons from a molecule. The lower the chemical potential, the greater the tendency for the molecule to accept an electron. The chemical potentials for the molecules under consideration are as follows: MIM (−3.74 eV), CIP (−4.39 eV), NIAC (−7.21 eV), NIPE (−5.63 eV), and NICL (−4.20 eV). The lowest chemical potential is for NIAC, thus emphasizing its strong tendency to accept an electron.

Chemical hardness (*η*) refers to the molecule's ability to be deformed or to transfer charge. Chemical hardness values: MIM = 2.79 eV, CIP = 1.87 eV, NIAC = 0.77 eV, NIPE = 0.84 eV, NICL = 0.97 eV. NIAC has the lowest hardness value, making it the softest molecule, followed by NIPE and NICL. Softness (*σ*) is the inverse of hardness and has a direct relationship with the polarizability and reactivity of the molecule. An increase in softness implies that the molecule would be more reactive and would be more inclined to undergo a charge transfer reaction. Softness values: MIM (0.18 eV^−1^), CIP (0.27 eV^−1^), NIAC (0.65 eV^−1^), NIPE (0.60 eV^−1^), NICL (0.51 eV^−1^). NIAC has the highest softness value, which reflects its high reactivity.

The electrophilicity index (*ω*) is the measure of the tendency of the molecule to accept electrons, which combines the concept of electron affinity as well as chemical potential. The higher the electrophilicity index, the stronger the electrophile. The electrophilicity index of the compounds is as follows: MIM (2.51 eV), CIP (5.16 eV), NIAC (33.74 eV), NIPE (18.94 eV), and NICL (9.07 eV). NIAC has a very high electrophilicity index, making it a very strong electrophile.

Although frontier molecular orbital parameters and global reactivity descriptors (HOMO–LUMO gap, electrophilicity index, and softness) provide valuable insights into the relative electronic properties and reactivity trends of the studied complexes, they can not be interpreted as direct predictors of antimicrobial efficacy or biological toxicity. In the present work, these DFT-derived parameters are used strictly as complementary tools to rationalize electronic stability and comparative reactivity among the synthesized complexes. The experimentally obtained antimicrobial activity remains the primary evidence of biological performance.

### 3.4. Biological activity

The growing worldwide problem of antimicrobial resistance calls for the ongoing search for new antimicrobial agents. Bacteria and fungi, including *E. coli*, *P. aeruginosa*, *B. cereus*, *S. aureus*, *C. albicans*, and *A. flavus*, are major health, agricultural, and industrial concerns. These microorganisms could contaminate water, soil, and foodstuffs, resulting in devastating consequences to the economy and the environment. For example, *P. aeruginosa* and *S. aureus* are well-known agents of hospital-acquired infections, while *E. coli* could induce foodborne diseases. *C. albicans* causes opportunistic infections in immunocompromised patients, while *A. flavus* produces aflatoxins that are powerful carcinogens and induce crop contamination. The need to address the problems caused by the microorganisms highlights the need to investigate new compounds, specifically multi-ligand transition metal complexes, for their antimicrobial activity.

The electronic absorption spectra of NiAC, NiPE, and NiCL remained approximately unchanged over the monitoring period in the DMSO-containing medium, indicating the absence of substantial gross decomposition under these experimental conditions. However, this observation does not necessarily demonstrate complete retention of the original inner-sphere coordination environments. In particular, ligand exchange involving water, chloride, acetate, perchlorate, or DMSO may occur without producing a large change in the overall electronic absorption profile. Therefore, the possibility that partially solvated or ligand-exchanged nickel species contribute to the biological response cannot be excluded. The similar antimicrobial activities observed for NiAC, NiPE, and NiCL may consequently reflect, at least in part, convergence toward related active species under biological conditions. Direct confirmation of such speciation would require more dedicated solution-state studies. This will be as an open question that the present data cannot resolve.

#### 3.4.1. Antibacterial activity

The inhibition zones obtained in the antibacterial assay are presented in Fig. 3a and Table 3. Under the agar-diffusion conditions employed, the parent compounds MIM and CIP produced inhibition zones of 8–10 and 10–11 mm, respectively, whereas the Ni(ii) complexes produced inhibition zones of 24–30 mm across the tested bacterial strains. NiAC gave the largest inhibition zone among the investigated complexes for several of the tested strains, including 30 mm against *B. cereus*. These observations indicate appreciable antibacterial activity of the synthesized Ni(ii) complexes under the conditions of the assay.

**Fig. 3:**
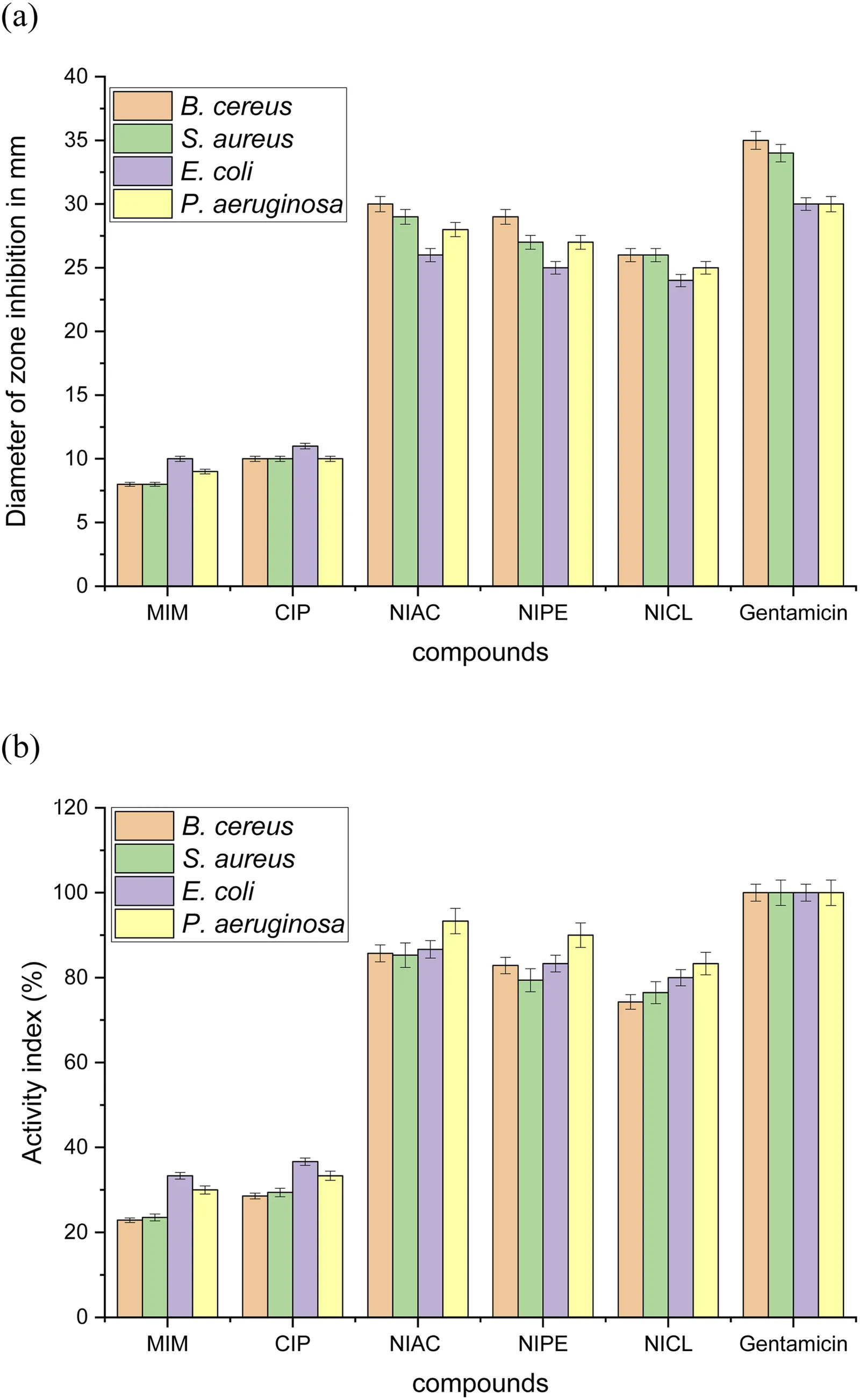
Antibacterial activity as (a): diameter of zone inhibition in mm, (b): activity index (%).

**Table 3:** Antibacterial activity as diameter of zone inhibition in mm

Diameter of zone inhibition in mm				
	Gram-negative bacteria		Gram-positive bacteria	
	*B. cereus*	*S. aureus*	*E. coli*	*P. aeruginosa*
MIM	8 ± 1.25	8 ± 1.18	10 ± 1.33	9 ± 1.22
CIP	10 ± 1.33	10 ± 1.34	11 ± 1.27	10 ± 1.24
NIAC	30 ± 1.18	29 ± 1.31	26 ± 1.26	28 ± 1.31
NIPE	29 ± 1.20	27 ± 1.24	25 ± 1.31	27 ± 1.33
NICL	26 ± 1.38	26 ± 1.17	24 ± 1.40	25 ± 1.25
Gentamicin	35 ± 1.22	34 ± 1.31	30 ± 1.27	30 ± 1.20

To facilitate comparison with the reference antibiotic, the activity index (AI, %) was calculated from the inhibition-zone diameters relative to gentamicin. The calculated values were 85.29–93.33% for NiAC, 79.41–90.00% for NiPE, and 74.29–83.33% for NiCL, whereas lower values were obtained for MIM and CIP. Thus, the activity-index values provide a descriptive comparison of the tested compounds with gentamicin under the same agar-diffusion conditions. Because no inferential statistical analysis was performed, these differences had not been interpreted as statistically significant.

#### 3.4.2. Anti-fungal activity

The antifungal activity of MIM and CIP against *C. albicans* and *A. flavus* produced inhibition zones of 8–10 mm, whereas the Ni(ii) complexes produced inhibition zones of 15–19 mm under the experimental conditions employed. NiAC showed inhibition zones of 19 and 17 mm against *C. albicans* and *A. flavus*, respectively, while NiPE produced zones of 18 and 17 mm and NiCL produced zones of 16 and 15 mm, respectively. These results indicate appreciable antifungal activity for the synthesized Ni(ii) complexes in the present agar-diffusion assay (Table 4 and Fig. 4).

**Table 4:** Anti-fungal activity as diameter of zone inhibition in mm

Comp.	Diameter of zone inhibition in mm	
	*C. albicans*	*A. flavus*
MIM	8 ± 1.22	8 ± 1.31
CIP	10 ± 1.41	9 ± 1.33
NIAC	19 ± 1.23	17 ± 1.24
NIPE	18 ± 1.18	17 ± 1.23
NICL	16 ± 1.39	15 ± 1.20
Clotrimazole	20 ± 1.30	18 ± 1.21

**Fig. 4:**
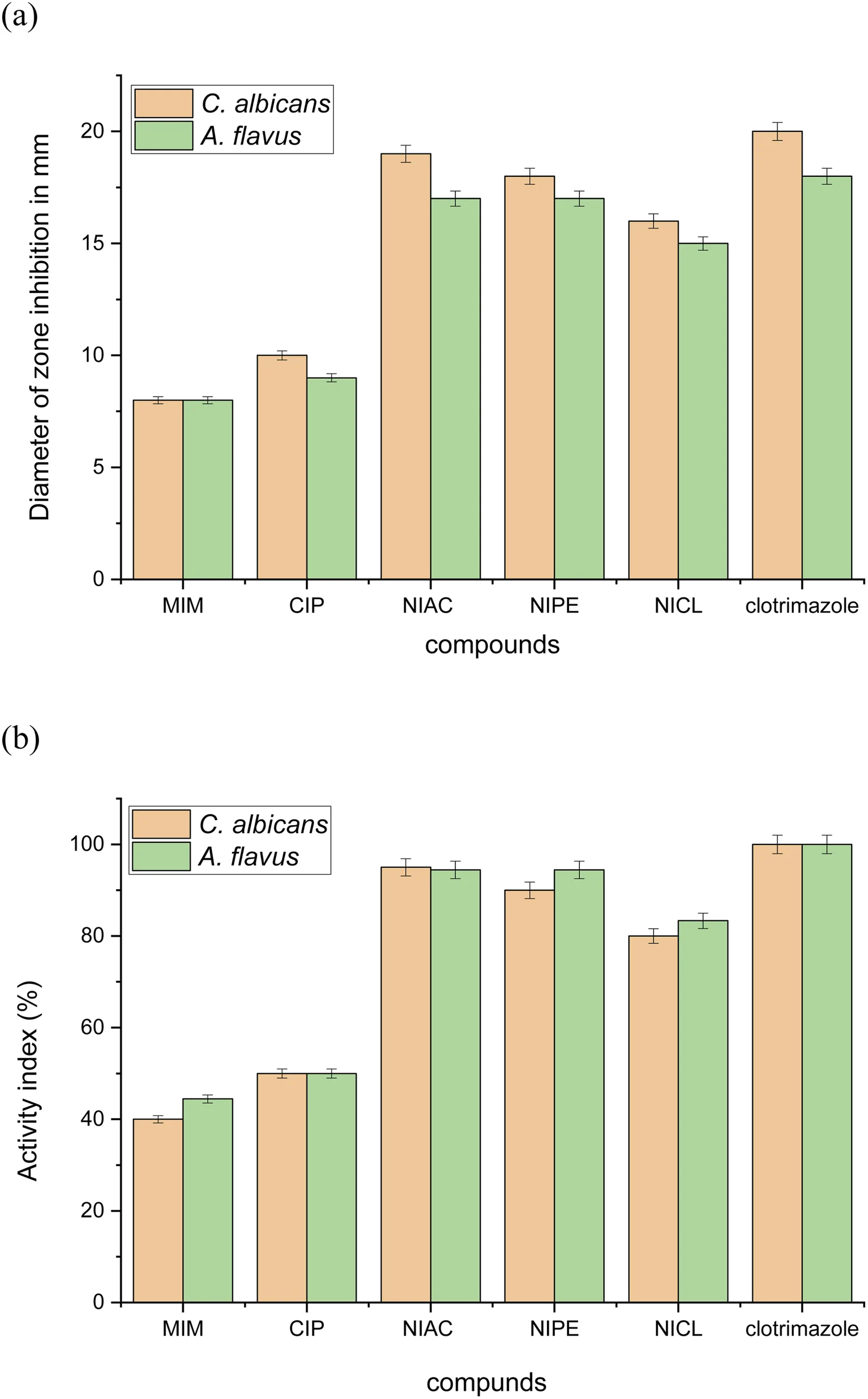
Antifungal activity as (a): diameter of zone inhibition in mm, (b): activity index (%).

The antifungal activity indices calculated relative to clotrimazole were 95.00 and 94.44% for NiAC, 90.00 and 94.44% for NiPE, and 80.00 and 83.33% for NiCL against *C. albicans* and *A. flavus*, respectively. The corresponding values for MIM and CIP were lower. These activity-index values provide a descriptive comparison with the reference antifungal agent under the conditions employed. In the absence of inferential statistical testing, the observed differences are not interpreted as statistically significant (Table 4).

#### 3.4.3. Minimum inhibition concentration (MIC, µM)

The MIC results for the tested compounds are summarized in Fig. S10 and Table 5. For the bacterial strains, MIM showed MIC values of 100 µM, whereas CIP showed values of 90–100 µM. The Ni(ii) complexes showed lower MIC values under the experimental conditions employed. NiAC gave a MIC of 40 µM against all four tested bacterial strains, while NiPE showed values of 40–50 µM and NiCL showed values of 50 µM.

**Table 5:** Antibacterial and antifungal activity as minimum inhibition concentration (MIC, µM)

Minimal inhibitory concentration (MIC) in µM						
	Gram-positive bacteria		Gram-negative bacteria		Fungi	
	*B. cereus*	*S. aureus*	*E. coli*	*P. aeruginosa*	*C. albicans*	*A. flavus*
MIM	100 ± 1.11	100 ± 1.25	100 ± 1.21	100 ± 1.16	100 ± 1.22	100 ± 1.34
CIP	90 ± 1.20	90 ± 1.20	90 ± 1.12	100 ± 1.22	90 ± 1.31	90 ± 1.33
NIAC	40 ± 1.15	40 ± 1.16	40 ± 1.30	40 ± 1.14	40 ± 1.35	40 ± 1.28
NIPE	50 ± 1.22	50 ± 1.22	40 ± 1.21	50 ± 1.25	40 ± 1.27	50 ± 1.31
NICL	50 ± 1.31	50 ± 1.20	50 ± 1.20	50 ± 1.38	50 ± 1.20	50 ± 1.25

For the fungal strains, MIM showed a MIC of 100 µM and CIP showed a MIC of 90 µM. NiAC and NiPE gave MIC values of 40–50 µM, whereas NiCL showed a MIC of 50 µM. Thus, lower MIC values were observed for the Ni(ii) complexes than for the corresponding free ligands in the present assay. These observations suggest that complex formation was associated with increased antimicrobial activity under the experimental conditions used; however, because no statistical significance testing was performed, the observed differences had been regarded as descriptive rather than statistically validated.

Based on the numerical MIC values obtained in this study, NiAC showed the lowest MIC values across the tested bacterial and fungal strains, followed by NiPE and NiCL. Accordingly, the apparent order based on the measured MIC values can be described as NiAC > NiPE/NICL > CIP > MIM. This ranking represents a descriptive comparison of the measured MIC values and had not been interpreted as evidence of statistically significant differences in antimicrobial potency.

Gentamicin was used as a standard reference antibiotic in the agar diffusion assay to enable comparison of inhibition zone diameters. MIC values for Gentamicin were not determined in this study, as its antimicrobial activity and MIC ranges against the tested microorganisms are well documented in the literature.

#### 3.4.4. General compounds order

Overall, the antimicrobial results show that the Ni(ii) complexes produced larger inhibition zones and lower MIC values than the corresponding free ligands under the experimental conditions employed. Among the investigated complexes, NiAC generally showed the largest inhibition zones and the lowest MIC values. The activity-index values also indicate that the Ni(ii) complexes reached relatively high percentages of the corresponding reference drugs under the agar-diffusion conditions used. These observations support an association between Ni(ii) complex formation and the observed antimicrobial response. However, because the present study did not include inferential statistical testing, these comparisons are descriptive and should not be interpreted as statistically significant differences. The relative ranking based on the measured MIC values was NiAC > NiPE/NICL > CIP > MIM.

#### 3.4.5. Possible relationship between chelation and antimicrobial activity

The higher antimicrobial activity observed for the Ni(ii) complexes compared with the corresponding free ligands may be associated with changes in physicochemical properties following coordination. Chelation can modify the charge distribution, polarity, and overall lipophilic character of a metal complex, which may influence its interaction with microbial membranes and its ability to reach intracellular sites. Such effects have been proposed for metal complexes; however, the present study did not directly measure membrane permeability, cellular uptake, or intracellular accumulation. Therefore, the chelation-based explanation should be considered a plausible interpretation of the observed activity rather than a directly demonstrated mechanism.

#### 3.4.6. Correlation with DFT parameters

The calculated global reactivity descriptors (HOMO–LUMO energy gap, chemical hardness, softness, electrophilicity, and chemical potential) primarily reflect the intrinsic electronic properties and reactivity of the complexes. These parameters should be interpreted as qualitative indicators of overall bio-interaction potential rather than quantitative predictors of antibacterial activity. In the present study, the antibacterial results of the Ni(ii) complexes are relatively comparable, showing only minor variations among the compounds; therefore, no direct one-to-one correlation can be established between antibacterial activity and the calculated descriptors. However, these parameters may still provide useful insight into the observed trends in antifungal activity and molecular docking affinities, where subtle differences in electronic structure more clearly influence ligand–target interactions.

The calculated electrophilicity indices provide additional information about the electron-accepting characteristics of the investigated compounds. The higher values calculated for the Ni(ii) complexes suggest that coordination substantially modifies their electronic properties. These descriptors may contribute to understanding differences in chemical reactivity and potential intermolecular interactions; however, they cannot by themselves establish enzyme deactivation or a direct causal relationship with antimicrobial activity. Thus, the DFT results are used here to describe electronic-property differences among the compounds rather than to establish a specific biological mechanism.

#### 3.4.7. MIC comparison with literature

The antibacterial activity of the synthesized Ni(ii) mixed-ligand complexes (NIAC, NIPE, and NICL), derived from ciprofloxacin (CIP) and 2-methylbenzimidazole (MIM), was evaluated against *E. coli* and compared with previously reported ciprofloxacin-based metal complexes to assess their relative efficacy. As summarized in Table 6, the present Ni(ii) complexes exhibit MIC values of 40 µM for NIAC and NIPE, and 50 µM for NICL. These results indicate that NIAC and NIPE display comparable antibacterial potency, while NICL shows slightly reduced activity, suggesting that the nature of the coordinated anion and the resulting coordination sphere plays a significant role in modulating biological performance. Specifically, the presence of acetate and perchlorate (NIAC and NIPE) appears to favor enhanced interaction with the microbial target compared to the chloride-containing octahedral complex (NICL), which may reduce lipophilicity and cellular uptake efficiency.

**Table 6:** A comparison of MIC against *E. coli* of NIAC, NIPE, and NICL complexes and other reported ciprofloxacin-based-mixed-ligand metal complexes and ciprofloxacin-based-binary metal complexes in literature

No.	Compounds	MIC_*E. Coli* (G^−^)_	Reference
1	NIAC	40 (µM)	This work
2	NIPE	40 (µM)	
3	NICL	50 (µM)	
4	CPFX1	13.89 ± 3.61 (µM)	Ciprofloxacin-based-mixed-ligand metal complexes^56^
5	CPFX2	20.85 ± 10.83 (µM)	
6	CPFX3	13.03 ± 3.61 (µM)	
7	CFAPNi	5.93 (µM)	Ciprofloxacin-based-binary metal complexes^57^
8	**CFAPFe**	6.00 (µM)	
9	**CFAPCu**	4.37 (µM)	
10	**CFAPPd**	4.26 (µM)	
11	**CFAPCr**	7.34 (µM)	
where:			
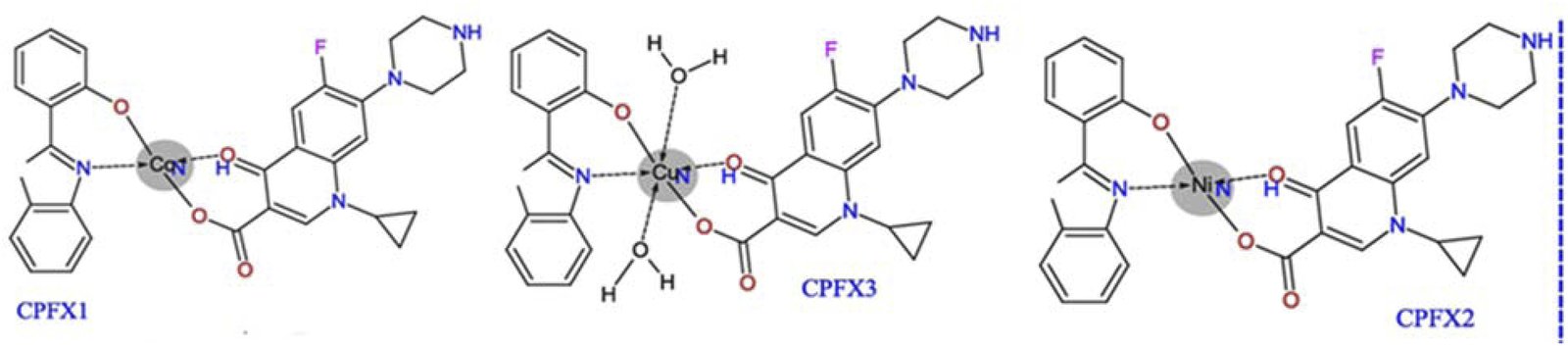			
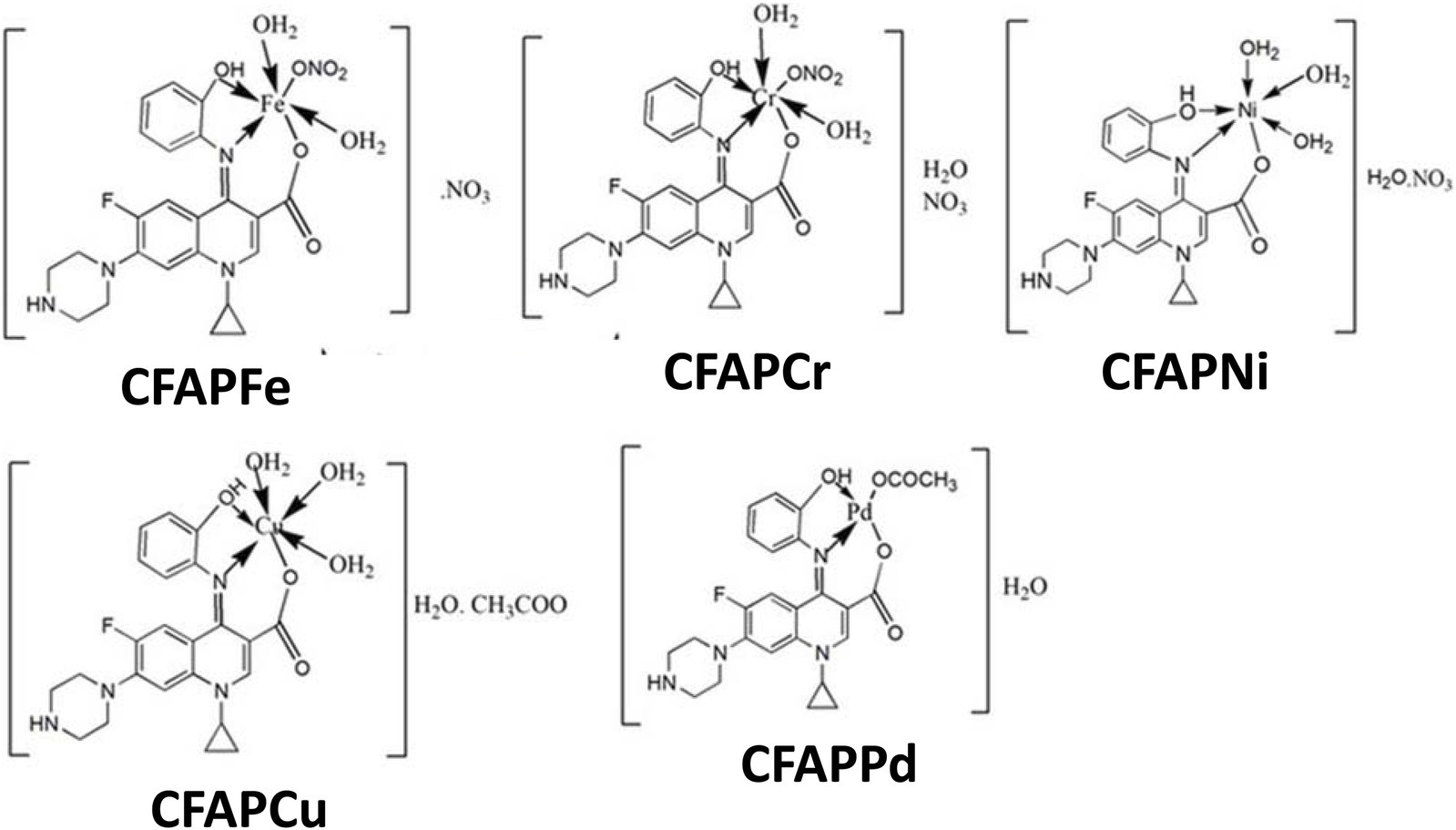			

When compared with literature reports, ciprofloxacin-based mixed-ligand metal complexes (CPFX1, CPFX2, and CPFX3) showed MIC values of 13.89 ± 3.61 µM, 20.85 ± 10.83 µM, and 13.03 ± 3.61 µM,^56^ while ciprofloxacin-based binary metal complexes such as CFAPNi, CFAPFe, CFAPCu, CFAPPd, and CFAPCr exhibited MIC values of 5.93 µM,6.00 µM, 4.37 µM, 4.26 µM, and 7.34 µM.^57^ The present Ni(ii) mixed-ligand complexes therefore show measurable antimicrobial activity, although direct comparison with literature values had been made cautiously because differences in experimental conditions, microorganisms, concentrations, and assay protocols may affect the reported MIC values. The present results suggest that the ligand environment and coordination sphere may influence the antimicrobial response, but this interpretation remains qualitative.

### 3.5. Molecular docking

The selection of *Escherichia coli* DNA gyrase B (4DUH) as a molecular target for investigating antibacterial activity is scientifically robust and well-established within antimicrobial drug discovery. DNA gyrase, a type II topoisomerase, is an essential bacterial enzyme responsible for introducing negative supercoils into bacterial DNA, a critical process for DNA replication, transcription, and recombination. The B subunit (GyrB), specifically the 24 kDa domain represented by 4DUH, contains the ATP-binding site. Inhibition of this site prevents ATP hydrolysis, which is necessary for the energy transduction required for DNA supercoiling. Consequently, blocking GyrB function leads to the accumulation of positively supercoiled DNA, ultimately disrupting vital cellular processes and leading to bacterial cell death. The high-resolution crystal structure of 4DUH, often co-crystallized with inhibitors, provides an excellent template for molecular docking studies, allowing for precise characterization of ligand-protein interactions and rational drug design.

To validate the reliability of the docking protocol, a redocking procedure was performed by extracting the co-crystallized native ligand, 4-([4′-methyl-2'-(propanoylamino)-4,5′-bi-1,3-thiazol-2-yl]amino) benzoic acid, from the DNA gyrase B (4DUH) active site and re-docking it into the same binding pocket under identical docking parameters. The generated docking pose was compared with the experimental crystallographic orientation to assess the accuracy of the docking algorithm. The results confirmed that the docking method was able to reliably reproduce the binding conformation of the native ligand within an acceptable root–mean–square deviation, having an RMSD of 1.017 (RMSD ≤ 2.0 Å), indicating the robustness and validity of the docking protocol used in this study. Fig. S11 illustrates this precise matching, signifying the reliability and effectiveness of the docking procedure.

Molecular docking investigation was performed to evaluate the interaction of the studied compounds with the crystal structure of the *E. coli* DNA gyrase B, and the results were visualized in Fig. 5, and tabulated in Table S10.

**Fig. 5:**
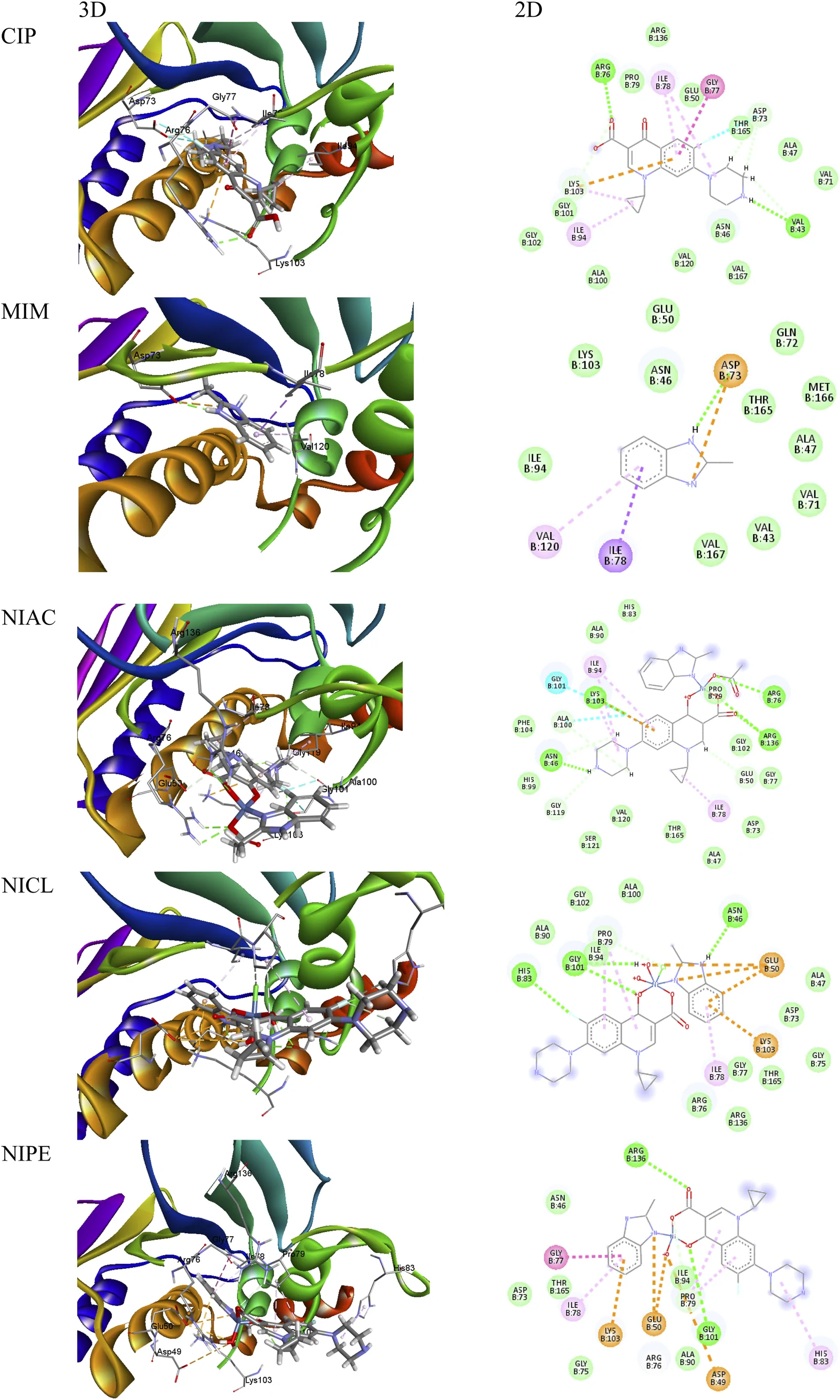
2D and 3D molecular docking poses of MIM, CIP ligands, and NIAC, NIPE, and NICL complexes against 4DUH receptor.

CIP ligand exhibits a binding energy of −7.10 kcal mol^−1^ with 4DUH, indicating a favorable interaction. Its binding profile is characterized by a diverse set of interactions crucial for its inhibitory action. Key interactions include an electrostatic interaction with LYS103 at a distance of 4.44 Å, suggesting a charge-based attraction within the binding pocket. Furthermore, CIP engages in a halogen bond with ASP73 at 3.08 Å, which can contribute to binding specificity and stability. Multiple hydrogen bonds are observed with ARG76 (2.30 Å), ASP73 (2.21 Å), LYS103 (3.50 Å), and VAL43 (2.88 Å). These hydrogen bonds are critical for anchoring the ligand within the active site, providing strong directional interactions. Hydrophobic contacts with ILE78 (4.33 Å), ILE94 (4.30 Å), and LYS103 (4.10 Å) further stabilize the complex by minimizing solvent exposure of non-polar regions.

MIM ligand demonstrates a binding energy of −6.40 kcal mol^−1^, which is less favorable than CIP, suggesting a comparatively weaker interaction with 4DUH. It forms an electrostatic interaction with ASP73 at 4.92 Å. A single hydrogen bond with ASP73 at 2.05 Å is a strong anchoring point, suggesting direct interaction with a critical residue in the active site. Hydrophobic interactions are observed with ILE78 (3.55 Å) and VAL120 (5.14 Å). The fewer and generally weaker interactions, particularly the absence of multiple hydrogen bonds and halogen interactions, contribute to its lower binding affinity.

The NIAC complex exhibits the most potent binding affinity among the studied compounds, with a binding energy of −8.50 kcal mol^−1^. This enhanced affinity compared to the parent ligands (CIP and MIM) underscores the profound impact of metal coordination on ligand-protein interactions. NIAC forms an electrostatic interaction with LYS103 (4.69 Å) and engages in halogen bonds with ALA100 (2.88 Å) and GLY101 (3.09 Å), indicating specific interactions with the protein backbone and side chains. A remarkable array of hydrogen bonds is observed with ALA100 (2.80 Å), ARG136 (3.10 Å), ARG76 (2.28 Å), ASN46 (2.55 Å), GLU50 (2.69 Å), GLY119 (3.03 Å), and LYS103 (2.61 Å). This extensive network of hydrogen bonds provides robust anchoring and precise orientation within the active site, explaining its superior binding. Hydrophobic interactions with ILE78 (4.95 Å), ILE94 (4.80 Å), and LYS103 (3.80 Å) further contribute to the overall stability.

The NIPE complex demonstrates a strong binding affinity with 4DUH, evidenced by a binding energy of −8.10 kcal mol^−1^. NIPE establishes multiple electrostatic interactions with ASP49 (5.48 Å), GLU50 (4.20 Å), and LYS103 (4.43 Å), indicating broad charge-based recognition within the binding pocket. Hydrogen bonds are formed with ARG136 (2.19 Å), GLY101 (3.39 Å), and PRO79 (3.44 Å), providing specific anchoring points. Hydrophobic interactions with HIS83 (4.55 Å), ILE78 (4.52 Å), and PRO79 (4.55 Å) further stabilize the complex.

The NICL complex also exhibits a strong interaction with 4DUH with a binding energy of −7.90 kcal mol^−1^. NICL forms electrostatic interactions with GLU50 (4.16 Å) and LYS103 (4.19 Å), indicating favorable charge–charge interactions. A significant number of hydrogen bonds are observed with ASN46 (2.04 Å), GLY101 (2.53 Å), HIS83 (2.66 Å), and PRO79 (2.48 Å). These interactions are crucial for precise positioning and strong binding within the active site. Hydrophobic contacts with ILE78 (4.73 Å) and PRO79 (4.36 Å) contribute to the overall stability of the ligand-protein complex. The combination of multiple electrostatic interactions and a strong hydrogen bonding network contributes to its high binding affinity, positioning NICL as a potent inhibitor of DNA gyrase after NIPE and NIAC.

Molecular docking studies against DNA gyrase B (PDB ID: 4DUH) were performed to examine the possible binding modes and interaction patterns of the studied compounds within the enzyme active site. The docking results showed favorable predicted interactions of the Ni(ii) complexes with residues located in or near the ATP-binding region of GyrB. However, the calculated binding energies represent computational estimates and should not be interpreted as direct measurements of enzyme inhibition or biological potency. Therefore, the docking results are considered supportive, hypothesis-generating evidence that identifies DNA gyrase B as a possible molecular target, rather than experimental proof of DNA gyrase inhibition.

### 3.6. Possible molecular basis of antibacterial activity

The synthesized Ni(ii) complexes exhibited appreciable antibacterial activity *in vitro*, as demonstrated by their inhibition-zone diameters and MIC values. In particular, NIAC and NIPE showed lower MIC values than the corresponding CIP and MIM ligands against the tested bacterial strains. These findings demonstrate an enhancement of antibacterial activity upon complex formation; however, the molecular mechanism responsible for this enhancement cannot be established solely from the present biological and spectroscopic data.

Molecular docking was therefore employed as a complementary computational approach to explore possible interactions of the synthesized compounds with DNA gyrase B (PDB ID: 4DUH). The docking analysis showed that NIAC, NIPE, and NICL can adopt favorable poses within the GyrB binding region and interact with several residues through hydrogen-bonding, electrostatic, halogen, and hydrophobic interactions. For example, the complexes showed interactions with residues including LYS103, ASP73, ARG76, ALA100, GLU50, and ARG136. These findings indicate that DNA gyrase B may represent a possible molecular target for the studied compounds.

Nevertheless, the docking results should be interpreted as predictive rather than mechanistic evidence. A favorable docking score or the presence of interactions with residues in an enzyme binding site does not demonstrate enzymatic inhibition under biological conditions. IAccordingly, the possibility that DNA gyrase contributes to the antibacterial action of the complexes remains a hypothesis that requires experimental verification.

2Other factors may also contribute to the observed antimicrobial activity, including differences in physicochemical properties, cellular penetration, membrane interactions, ligand release, and interactions with other intracellular targets. The present experimental results do not allow these possibilities to be distinguished. Therefore, the antimicrobial activity observed in this study should be regarded as an experimentally established phenotypic effect, whereas the DNA gyrase interaction represents a plausible molecular explanation suggested by the docking analysis.

## 4. Conclusion

The present study reports the successful synthesis and comprehensive characterization of three novel mixed-ligand Ni(ii) complexes derived from ciprofloxacin (CIP) and 2-methylbenzimidazole (MIM), namely NiAC, NiPE, and NiCL. The formation of the complexes with a 1 : 1 : 1 (Ni:CIP:MIM) stoichiometry was conclusively established by elemental analysis and Job's continuous variation method, in full agreement with spectroscopic and mass spectral data. Physicochemical investigations confirmed distinct coordination environments. FT-IR analysis verified bi-dentate coordination of ciprofloxacin through carboxylate and carbonyl oxygen atoms, while MIM coordinates *via* the imidazole nitrogen atom. UV-vis spectroscopy and magnetic moment measurements supported distorted tetrahedral geometry for NiAC and NiPE, whereas NiCL exhibited an octahedral geometry, consistent with ligand field expectations and electronic configuration of Ni(ii). Molar conductivity measurements confirmed the non-electrolytic nature of NiAC and NiCL, while NiPE behaves as a 1 : 1 electrolyte, indicating the presence of an outer-sphere perchlorate ion. Thermogravimetric analysis further confirmed the proposed coordination modes, including the presence or absence of coordinated water molecules, and demonstrated high thermal stability with final residues corresponding to NiO. DFT calculations strongly support the experimental findings, revealing consistent optimized geometries and frontier molecular orbital distributions. The calculated reactivity descriptors indicated enhanced electrophilicity and reduced HOMO–LUMO energy gap for NiAC, suggesting higher chemical reactivity compared to NiPE and NiCL. These theoretical results correlate well with the observed biological activity trends. All investigated compounds exhibited measurable antibacterial and antifungal activities under the tested conditions. The Ni(ii) complexes generally showed enhanced activity compared with the corresponding free ligands, with NiAC displaying the lowest MIC values. Molecular docking studies against DNA gyrase B (PDB: 4DUH) proposed good binding affinities, particularly for NiAC, through hydrogen bonding and electrostatic interactions. Overall, the combined experimental and theoretical results consistently demonstrate that mixed-ligand coordination enhances the structural diversity, electronic properties, and biological activity of Ni(ii) complexes.

## Author contributions

H. M. Abd El-Lateef: investigation, resources, formal analysis, funding acquisition, writing – original draft, writing – review & editing. H. Eltayeb: formal analysis, writing – original draft, writing-review & editing. M. M. Khalaf: investigation, resources, formal analysis, funding acquisition, writing – original draft, writing – review & editing. A. Abdou: investigation, methodology, resources, formal analysis, data curation, writing – original draft, writing – review & editing.

## Conflicts of interest

The authors declare that they have no known competing financial interests or personal relationships that could have appeared to influence the work reported in this paper.

## Supplementary Material

RA-016-D6RA04805F-s001

## Data Availability

The authors confirm that the data supporting the findings of this study are available within the article and its supplementary information (SI). Supplementary information is available. See DOI: https://doi.org/10.1039/d6ra04805f.
