# Supplementary material for: Structural diversity of new mixed-ligand Ni(ii) complexes derived from ciprofloxacin and 2-methylbenzimidazole: synthesis, characterization, DFT insights, antimicrobial activity and DNA gyrase B (4DUH) docking
Source: RSC Adv. 2026 Sep 29;16(45):58467–82. doi: 10.1039/d6ra04805f (PMC13621423; doi:10.1039/d6ra04805f)
Supplement: Supplementary file 1 [file RA-016-D6RA04805F-s001.pdf]

Supplementary Information (SI) for RSC Advances.
This journal is © The Royal Society of Chemistry 2026

Supplementary data

Structural Diversity of New Mixed-Ligand Ni(II) Complexes Derived from Ciprofloxacin and 2-Methylbenzimidazole: Synthesis, Characterization, DFT Insights, Antimicrobial Activity and DNA Gyrase B (4DUH) Docking

Hany M. Abd El-Lateef[1], Huda Eltayeb[2], Mai M. Khalaf[1], Aly Abdou[3*]

[1]Department of Chemistry, College of Science, King Faisal University, Al-Ahsa 31982, Saudi Arabia

[2] Department of Basic Sciences, College of Medicine, Princess Nourah bint Abdulrahman University, P.O. Box 84428, Riyadh 11671, Saudi Arabia

[3]Department of Chemistry, Faculty of Science, Sohag University, Sohag 82524, Egypt

* Corresponding authors: aly_abdou@science.sohag.edu.eg (A. Abdou)

S. 1. Materials and Instruments

S. 1.1. Materials

The materials used in this study were of analytical reagent grade. These included Nickel(II) chloride hexahydrate ($NiCl_2.6H_2O$), Nickel(II) acetate tetrahydrate ($Ni(CH_3COO)_2.4H_2O$), Nickel(II) perchlorate hexahydrate ($Ni(ClO_4)_2.6H_2O$), and ciprofloxacin, along with organic solvents such as ethanol, dimethyl sulfoxide, and dimethyl formamide.

S. 1.2. Instruments

Elemental analyses for carbon, hydrogen, and nitrogen were carried out using a PerkinElmer 2408 elemental analyzer, while the metal content was determined by atomic absorption spectroscopy using a PerkinElmer 2380 instrument. Infrared spectra were recorded on a Bruker FT-IR spectrometer (model 8101) in the range 4000-400 cm^{-1} to identify functional groups and metal-ligand coordination modes. Electronic absorption spectra were measured using a Jasco V-750 UV-Vis spectrophotometer for 10^{-3} mol L^{-1} solution in Ethanol, providing information on ligand-centered and metal-centered transitions. Mass spectrometric analyses were performed using a Thermo Scientific ISQ gas chromatography-mass spectrometry system equipped with a direct inlet probe and operating under electron ionization conditions, enabling confirmation of molecular weights and fragmentation patterns. Thermal behavior of the ligands and complexes was investigated by thermogravimetric and differential thermal analyses using a Shimadzu thermal analyzer (model 60H) at a heating rate of 10 °C min^{-1} under an inert atmosphere, allowing assessment of thermal stability and the presence of coordinated or lattice solvent molecules. Molar conductance measurements were carried out at room temperature using a Jenway conductivity meter (model 4320) for 10^{-3} mol L^{-1} solutions in Ethanol to evaluate the electrolytic nature of the complexes. Magnetic susceptibility measurements of powdered samples were performed using a Bartington magnetic susceptibility balance, and the effective

magnetic moments were calculated to support the proposed geometries and electronic configurations. In addition, the spectrophotometric continuous variation (Job's) method was employed to determine the metal-to-ligand stoichiometry of the synthesized complexes in solution.

S.2 Synthesis of the 2-methyl-benzimidazole (MIM)

The MIM ligand was made as described before [1-3] on a water bath at 70°C, a combination of o-phenylene-diamine (50.0 mmol, 5.4 g) and acetic acid (50.0 mmol, 3.0 g) was refluxed for 8.0 h in the presence of hydrochloric acid (4.0 N, 10.0 ml). The reaction mixture was then chilled and neutralized with potassium bicarbonate until the effervescence was no longer visible. The resulting precipitate was filtered and re-crystallized from ethanol, as shown in Scheme (S.1).

MIM ligand, Color, buff, m.p. 175-177 °C, yield, 65 %. Anal. Found (calc.) for ($C_8H_8N_2$, 132 g/mol) (%): C, 72.70 (72.73), H, 6.10 (6.06), N, 21.20 (21.21). ^{1}H-NMR, (DMSO-d_6) (δ, ppm): 12.23 (s, 1H, -NH), 7.10–7.45 (m, 4H, Aromatic protons), 2.32 (s, 1H, -CH_3 group). Fourier transform infrared spectroscopy (FT-IR), (υ, cm^{-1}): 3108 (-NH), 2950 (-$CH_{aliphatic}$), 1617 (C=N). UV-vis. (λ_{max}, nm): 236 ($\pi \rightarrow \pi^*$) and 292 ($n \rightarrow \pi^*$).

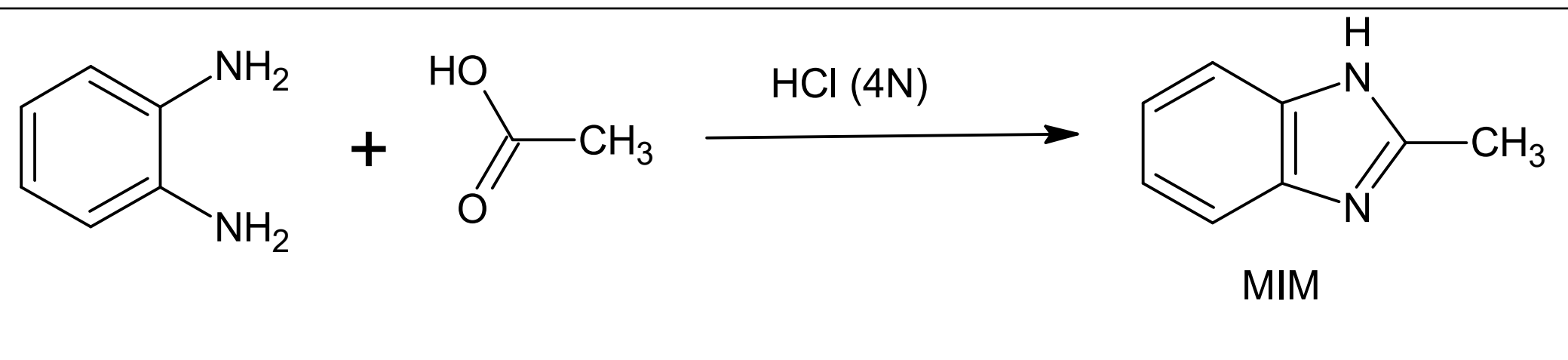

Scheme (S.1): Synthesis of MIM ligand

S.3. DFT calculation

All quantum chemical calculations for the free ligands and their complexes were performed using the ORCA 6.1.0 [4]. The geometrical optimizations were carried out within the framework of Density Functional Theory (DFT) employing the B3LYP hybrid functional. The def2-TZVP basis set was applied to all non-metal atoms (C, H, N, and O), while the def2-SVP basis set was employed for the transition-metal centers [5-7]. The initial molecular geometries and corresponding input files were generated using the Avogadro molecular modeling package [8]. Upon completion of geometry optimization, the frontier molecular orbitals (HOMO and LUMO) were visualized and analyzed using the Multiwfn program [9]. The global reactivity descriptors, ionization potential (IP), electron affinity (*EA*), energy gap (ΔE), electronegativity (χ), chemical potential (μ), chemical hardness (η), softness (σ), electrophilicity index (ω), and nucleophilicity (Nu), were calculated [10-13], see equations (1-9). These parameters provided crucial insights into the reactivity, and electronic behavior of the ligands and their complexes.

$IP=-E_{HOMO}$	equation (1)
$EA=-E_{LUMO}$	equation (2)
$\Delta E = IP - EA$	equation (3)
$X=-[E_{LUMO}+E_{HOMO}]/2$	equation (4)
$\mu=(E_{HOMO}+E_{LUMO})/2$	equation (5)
$\eta=-[E_{LUMO}-E_{HOMO}]/2$	equation (6)
$\sigma=1/\eta$	equation (7)
$\omega=\mu^2/2\eta$	equation (8)
$Nu = 1/\omega$	equation (9)

S.4. Antimicrobial testing *in vitro*

***In Vitro* Antibacterial activity**

The *in vitro* antibacterial activities of the understudy compounds were evaluated against two Gram-positive (*Bacillus cereus*, *Staphylococcus aureus*) and two Gram-negative (*Escherichia coli*, *Pseudomonas aeruginosa*), using the disk diffusion method [14]. A 100 µL suspension of each bacterium, containing approximately 0.5×10^6 CFU, was evenly spread onto Muller Hinton Agar plates using a sterile swab. The compounds were dissolved in DMSO at a concentration of 1.5 mg/mL, and sterile discs (6 mm in diameter) were impregnated with the solutions and placed on the inoculated agar surface. The plates were inverted and incubated at 37 °C for 24 hours. As the samples diffused, bacterial growth was inhibited around the discs. After incubation, the antibacterial efficacy was assessed by measuring the diameter of the inhibition zones in millimeters. DMSO, serving as the solvent, showed no effect on bacterial growth and was used as a negative control. All measurements were performed in triplicate, and results are expressed as mean ± standard deviation (SD, n = 3).

***In Vitro* Aantifungal activity**

The antifungal activity screening was conducted using the disk diffusion method. The antifungal properties of the ligand and its complexes were tested against two fungal strains, *Candida albicans* and *Aspergillus flavus*. In this procedure, discs were soaked in compound solutions at a concentration of 1.5 mg/mL in DMSO and placed at various positions on Sabouraud Dextrose Agar plates inoculated with fungal spore suspensions (10^5 CFU/mL) [15]. The plates with *Candida albicans* and *Aspergillus flavus* were incubated at 32 °C for 24 hours and 37 °C for 7 days, respectively. Following incubation, the inhibition zones (measured in millimeters) formed around each disc was recorded to assess the antifungal activity of the compounds. All measurements were performed in triplicate, and results are expressed as mean ± standard deviation (SD, n = 3).

Activity Index (%)

The effectiveness of the antibiotic Gentamicin and the antifungal agent clotrimazole was also evaluated using the same methods to determine their efficacy as standard antibacterial and antifungal agents against the tested microorganisms. The activity index (AI, %) was calculated according to the following equation: AI (%) = (inhibition zone diameter of the test compound / inhibition zone diameter of Gentamicin) × 100 [16]. This comparison provided a comprehensive assessment of the antibacterial and antifungal activities of the compounds in relation to the standard drugs, Gentamicin and clotrimazole.

Minimum inhibitory concentration (MIC)

The Minimum Inhibitory Concentrations (MIC) were determined using the broth dilution method. In this procedure, each sample was dissolved in DMSO at various concentrations ranging from 10.0 to 200 µM under sterile conditions. To each tube containing the sample, 650 µL of Muller Hinton Broth and 100 µL of the tested microorganism were added. The tubes were then incubated at 37 °C for 24 hours. The MIC was defined as the lowest concentration of the antibacterial sample that inhibited visible microbial growth, as indicated by the absence of turbidity in the mixture [17].

S.5. Structure elucidation of the MIM ligand

The synthesized 2-methylbenzimidazole (MIM) ligand was obtained as a buff-colored crystalline solid in a moderate yield (65%) with a melting point of 175-177 °C, indicating good purity and consistency with reported benzimidazole derivatives.

Elemental analysis showed excellent agreement between the experimental and calculated values for $C_8H_8N_2$ (C: 72.70% vs. 72.73%, H: 6.10% vs. 6.06%, N: 21.20% vs. 21.21%), confirming the proposed molecular formula and high purity of the ligand.

The ^{1}H-NMR spectrum recorded in DMSO-d6 provides clear structural evidence for MIM formation. A characteristic singlet at δ = 12.23 ppm is assigned to the imidazole -NH proton, confirming the presence of the benzimidazole moiety. The multiplet observed in the aromatic region at δ = 7.10-7.45 ppm corresponds to four aromatic protons of the fused benzene ring. Additionally, a singlet at δ = 2.32 ppm is attributed to the methyl ($-CH_3$) group attached to the heterocyclic ring, further confirming successful cyclization and substitution.

The FT-IR spectrum supports these findings. The band at 3108 cm^{-1} is assigned to the stretching vibration of the -NH group, while the band at 2950 cm^{-1} corresponds to aliphatic C-H stretching of the methyl group. The strong absorption at 1617 cm^{-1} is characteristic of the azomethine (C=N) group within the imidazole ring, confirming ring formation and heterocyclic integrity.

The UV-Vis spectrum exhibits two distinct absorption bands at 236 nm and 292 nm, corresponding to $\pi \rightarrow \pi^*$ and $n \rightarrow \pi^*$ electronic transitions, respectively.

Overall, the combined analytical, spectroscopic, and physicochemical data conclusively confirm the successful synthesis of the MIM ligand with the expected structure, and electronic features.

MIM ligand, Color, buff, m.p. 175-177 °C, yield, 65 %. Anal. Found (calc.) for ($C_8H_8N_2$, 132 g/mol) (%): C, 72.70 (72.73), H, 6.10 (6.06), N, 21.20 (21.21). ^{1}H-NMR, (DMSO-d_6) (δ,

ppm): 12.23 (s, 1H, -NH), 7.10–7.45 (m, 4H, Aromatic protons), 2.32 (s, 1H, $-CH_3$ group). Fourier transform infrared spectroscopy (FT-IR), (υ, cm^{-1}): 3108 (-NH), 2950 ($-CH_{aliphatic}$), 1617 (C=N). UV-vis. (λ_{max}, nm): 236 ($\pi \rightarrow \pi^*$) and 292 ($n \rightarrow \pi^*$).

S.6. Stoichiometry of the synthesized mixed-ligand complexes

The continuous variation method was used to establish the composition of the ternary M : MIM: CIP) complexes [18, 19]. The molar fractions of two of the components were varied continuously, keeping their total concentration constant in the presence of a large excess of the third component. Under these conditions, the ternary system was modified to a pseudo-binary system [18, 19].

Firstly, for determination of the stoichiometry of M: MIM in the presence of CIP as mixed ligands. A series of solutions containing different ratios of M:MIM, were prepared (in the presence of an excess of CIP), keeping the total concentration of both M ion and MIM constant. The ratio of M:MIM, was determined from the relationship between absorbance (Abs) (at the λ_{max} of the target complex) and mole fraction of MIM (MIM/(MIM+M)).

Secondly, the ratio of M:CIP was determined as described above in the presence of excess (MIM). A series of solutions containing different ratios of M:CIP, were prepared (in the presence of an excess of MIM), keeping the total concentration of both M ion and CIP constant. The ratio of M:CIP, was determined from the relationship between absorbance (Abs) (at the λ_{max} of the target complex) and mole fraction of CIP (CIP/(CIP+M)).

The results proved the 1:1:1 (M:MIM:CIP.) ternary complexes were formed for NIAC, NiPE, and NiCL complexes.

Table (S.1): Physical properties of the of the NiAC, NiPE, and NiCL					
	**ligands**		**complexes**		
	**CIP**	**MIM**	**NiAC**	**NiPE**	**NiCL**
Color	White	buff	Blue-violet	Bale blue	green
Melting point (^{o}C)	255	175-177	> 300	> 300	> 300
Yield (%)	---	65	87	88	85

Table (S.2): elemental analysis: found (calculated) of the of the NiAC, NiPE, and NiCL					
	**ligands**		**complexes**		
	**CIP**	**MIM**	**NiAC**	**NiPE**	**NiCL**
C	----	72.70 (72.73)	55.08 (55.89)	47.24 (47.02)	50.40 (50.66)
H	----	6.10 (6.06)	4.37 (4.86)	4.51 (4.26)	4.64 (4.93)
N	----	21.20 (21.21)	12.34 (12.07)	10.72 (10.97)	11.55 (11.82)
M	----	----	10.33 (10.12)	9.38 (9.19)	10.21 (9.90)

Table (S.3): conductivity in ethanol of the of the NiAC, NiPE, and NiCL					
	**ligands**		**complexes**		
	**CIP**	**MIM**	**NiAC**	**NiPE**	**NiCL**
μ_v, Ω^{-1}cm^{2}mol^{-1}	----	----	9.62	68.52	10.85

Table (S.4): UV-Vis. Spectra of the of the NiAC, NiPE, and NiCL					
	**ligands**		**complexes**		
	**CIP**	**MIM**	**NiAC**	**NiPE**	**NiCL**
λ_{max}, nm	324, 365	236 and 292	640 nm (15625.00 cm^{-1})	610 nm (16393.44 cm^{-1})	675 nm (14814.81 cm^{-1})
			[20]	[20]	

Table (S.5): Magnetic of the of the NiAC, NiPE, and NiCL					
	**ligands**		**complexes**		
	**CIP**	**MIM**	**NiAC**	**NiPE**	**NiCL**
μ_{eff} (B.M)	----	----	3.73	3.68	2.95

Table (S.6): IR of the of the NiAC, NiPE, and NiCL					
	**ligands**		**complexes**		
	**CIP**	**MIM**	**NiAC**	**NiPE**	**NiCL**
υ (-C=O)$_{CIP}$	1628	----	1655	1648	1650
ν(-COO)$_{asymmetric}$	1702	----	1611	1617	1609
ν(-COO)$_{symmetric}$	----	----	1382	1380	1388
Δ[ν(-COO)$_{asymmetric}$ - ν(-COO)$_{symmetric}$]	----	----	229	237	221
υ (-N-H)$_{MIM}$	----	3109	3110	3113	3107
υ (-C=N)$_{MIM}$	----	1618	1588	1585	1889
υ (M-O)	----	----	572	568	570
υ (M-N)	----	----	504	508	502

Table (S.7): bonds angles and Dihedral angles of the NIAC, NIPE, and NICL complexes					
NiAC		NiPE		NiCL	
O(63)-Ni(67)-O(42)	110.15	O(60)-Ni(63)-O(42)	122.27	O(49)-Ni(67)-O(46)	94.81
O(63)-Ni(67)-O(39)	109.60	O(60)-Ni(63)-O(39)	112.67	O(49)-Ni(67)-Cl(25)	85.52
O(63)-Ni(67)-N(7)	119.65	O(60)-Ni(63)-N(7)	109.00	O(49)-Ni(67)-N(13)	87.19
O(42)-Ni(67)-O(39)	107.57	O(42)-Ni(63)-O(39)	91.96	O(49)-Ni(67)-O(4)	93.45
O(42)-Ni(67)-N(7)	104.33	O(42)-Ni(63)-N(7)	108.84	O(49)-Ni(67)-O(1)	178.49
O(39)-Ni(67)-N(7)	104.83	O(39)-Ni(63)-N(7)	111.00	O(46)-Ni(67)-Cl(25)	87.20
Ni(67)-O(63)-C(60)	109.34	Ni(63)-O(60)-H(62)	104.57	O(46)-Ni(67)-N(13)	177.78
H(66)-C(61)-H(65)	109.47	Ni(63)-O(60)-H(61)	104.61	O(46)-Ni(67)-O(4)	91.63
H(66)-C(61)-H(64)	109.46	H(62)-O(60)-H(61)	104.34	O(46)-Ni(67)-O(1)	85.65
H(66)-C(61)-C(60)	109.46	Ni(63)-O(42)-C(40)	108.93	Cl(25)-Ni(67)-N(13)	92.00
H(65)-C(61)-H(64)	109.47	O(42)-C(40)-O(41)	119.27	Cl(25)-Ni(67)-O(4)	178.38
H(65)-C(61)-C(60)	109.48	O(42)-C(40)-C(26)	118.28	Cl(25)-Ni(67)-O(1)	93.06
H(64)-C(61)-C(60)	109.48	O(41)-C(40)-C(26)	120.90	N(13)-Ni(67)-O(4)	89.20
O(63)-C(60)-O(62)	119.97	Ni(63)-O(39)-C(25)	115.78	N(13)-Ni(67)-O(1)	92.33
O(63)-C(60)-C(61)	120.08	H(59)-C(37)-H(58)	122.58	O(4)-Ni(67)-O(1)	87.98
O(62)-C(60)-C(61)	119.96	H(59)-C(37)-C(36)	114.18	Ni(67)-O(49)-C(47)	122.63
Ni(67)-O(42)-C(40)	109.66	H(59)-C(37)-C(35)	114.31	O(49)-C(47)-O(48)	118.07

O(42)-C(40)-O(41)	118.90	H(58)-C(37)-C(36)	113.98	O(49)-C(47)-C(33)	121.67
O(42)-C(40)-C(26)	122.14	H(58)-C(37)-C(35)	115.74	O(48)-C(47)-C(33)	120.08
O(41)-C(40)-C(26)	118.95	C(36)-C(37)-C(35)	60.16	Ni(67)-O(46)-C(32)	126.48
Ni(67)-O(39)-C(25)	118.48	H(57)-C(36)-H(56)	122.54	H(66)-C(44)-H(65)	122.56
H(59)-C(37)-H(58)	112.06	H(57)-C(36)-C(37)	113.50	H(66)-C(44)-C(43)	114.14
H(59)-C(37)-C(36)	116.27	H(57)-C(36)-C(35)	116.29	H(66)-C(44)-C(42)	114.36
H(59)-C(37)-C(35)	117.73	H(56)-C(36)-C(37)	113.93	H(65)-C(44)-C(43)	113.91
H(58)-C(37)-C(36)	122.98	H(56)-C(36)-C(35)	114.46	H(65)-C(44)-C(42)	115.81
H(58)-C(37)-C(35)	119.18	C(37)-C(36)-C(35)	60.13	C(43)-C(44)-C(42)	60.17
C(36)-C(37)-C(35)	59.26	H(55)-C(35)-C(37)	117.01	H(64)-C(43)-H(63)	122.53
H(57)-C(36)-H(56)	108.10	H(55)-C(35)-C(36)	118.83	H(64)-C(43)-C(44)	113.45
H(57)-C(36)-C(37)	123.51	H(55)-C(35)-N(27)	119.21	H(64)-C(43)-C(42)	116.38
H(57)-C(36)-C(35)	122.02	C(37)-C(35)-C(36)	59.71	H(63)-C(43)-C(44)	113.87
H(56)-C(36)-C(37)	116.36	C(37)-C(35)-N(27)	113.57	H(63)-C(43)-C(42)	114.47
H(56)-C(36)-C(35)	120.22	C(36)-C(35)-N(27)	114.57	C(44)-C(43)-C(42)	60.15
C(37)-C(36)-C(35)	60.08	H(54)-N(34)-C(33)	107.70	H(62)-C(42)-C(44)	116.73
H(55)-C(35)-C(37)	123.14	H(54)-N(34)-C(32)	107.70	H(62)-C(42)-C(43)	119.05
H(55)-C(35)-C(36)	123.14	C(33)-N(34)-C(32)	110.73	H(62)-C(42)-N(34)	119.17
H(55)-C(35)-N(27)	109.46	H(53)-C(33)-H(52)	107.98	C(44)-C(42)-C(43)	59.68
C(37)-C(35)-C(36)	60.67	H(53)-C(33)-N(34)	110.42	C(44)-C(42)-N(34)	113.48
C(37)-C(35)-N(27)	116.58	H(53)-C(33)-C(31)	109.77	C(43)-C(42)-N(34)	114.75
C(36)-C(35)-N(27)	116.60	H(52)-C(33)-N(34)	109.25	H(61)-N(41)-C(40)	107.69
H(54)-N(34)-C(33)	116.35	H(52)-C(33)-C(31)	108.94	H(61)-N(41)-C(39)	107.70
H(54)-N(34)-C(32)	116.37	N(34)-C(33)-C(31)	110.44	C(40)-N(41)-C(39)	110.74
C(33)-N(34)-C(32)	95.83	H(51)-C(32)-H(50)	107.98	H(60)-C(40)-H(59)	107.97
H(53)-C(33)-H(52)	109.82	H(51)-C(32)-N(34)	109.27	H(60)-C(40)-N(41)	110.43
H(53)-C(33)-N(34)	114.07	H(51)-C(32)-C(30)	108.95	H(60)-C(40)-C(38)	109.77
H(53)-C(33)-C(31)	108.31	H(50)-C(32)-N(34)	110.52	H(59)-C(40)-N(41)	109.24
H(52)-C(33)-N(34)	103.98	H(50)-C(32)-C(30)	109.87	H(59)-C(40)-C(38)	108.93
H(52)-C(33)-C(31)	109.84	N(34)-C(32)-C(30)	110.19	N(41)-C(40)-C(38)	110.44
N(34)-C(33)-C(31)	110.75	H(49)-C(31)-H(48)	108.41	H(58)-C(39)-H(57)	107.99
H(51)-C(32)-H(50)	109.83	H(49)-C(31)-C(33)	109.09	H(58)-C(39)-N(41)	110.51
H(51)-C(32)-N(34)	114.06	H(49)-C(31)-N(29)	111.12	H(58)-C(39)-C(37)	109.88
H(51)-C(32)-C(30)	108.30	H(48)-C(31)-C(33)	109.62	H(57)-C(39)-N(41)	109.26
H(50)-C(32)-N(34)	103.99	H(48)-C(31)-N(29)	109.23	H(57)-C(39)-C(37)	108.94
H(50)-C(32)-C(30)	109.81	C(33)-C(31)-N(29)	109.34	N(41)-C(39)-C(37)	110.21
N(34)-C(32)-C(30)	110.77	H(47)-C(30)-H(46)	107.81	H(56)-C(38)-H(55)	108.41
H(49)-C(31)-H(48)	109.60	H(47)-C(30)-C(32)	109.36	H(56)-C(38)-C(40)	109.09
H(49)-C(31)-C(33)	108.16	H(47)-C(30)-N(29)	109.02	H(56)-C(38)-N(36)	111.13
H(49)-C(31)-N(29)	114.54	H(46)-C(30)-C(32)	109.83	H(55)-C(38)-C(40)	109.61
H(48)-C(31)-C(33)	110.92	H(46)-C(30)-N(29)	111.42	H(55)-C(38)-N(36)	109.23
H(48)-C(31)-N(29)	104.50	C(32)-C(30)-N(29)	109.36	C(40)-C(38)-N(36)	109.34
C(33)-C(31)-N(29)	109.11	C(31)-N(29)-C(30)	117.71	H(54)-C(37)-H(53)	107.79
H(47)-C(30)-H(46)	109.60	C(31)-N(29)-C(24)	120.82	H(54)-C(37)-C(39)	109.36

H(47)-C(30)-C(32)	110.95	C(30)-N(29)-C(24)	120.88	H(54)-C(37)-N(36)	109.03
H(47)-C(30)-N(29)	104.52	H(45)-C(28)-N(27)	118.58	H(53)-C(37)-C(39)	109.82
H(46)-C(30)-C(32)	108.15	H(45)-C(28)-C(26)	119.52	H(53)-C(37)-N(36)	111.42
H(46)-C(30)-N(29)	114.54	N(27)-C(28)-C(26)	121.87	C(39)-C(37)-N(36)	109.38
C(32)-C(30)-N(29)	109.07	C(35)-N(27)-C(28)	118.20	C(38)-N(36)-C(37)	117.69
C(31)-N(29)-C(30)	117.94	C(35)-N(27)-C(20)	121.83	C(38)-N(36)-C(31)	120.85
C(31)-N(29)-C(24)	109.01	C(28)-N(27)-C(20)	119.32	C(37)-N(36)-C(31)	120.91
C(30)-N(29)-C(24)	109.13	C(40)-C(26)-C(28)	120.73	H(52)-C(35)-N(34)	118.16
H(45)-C(28)-N(27)	117.87	C(40)-C(26)-C(25)	118.19	H(52)-C(35)-C(33)	119.60
H(45)-C(28)-C(26)	117.92	C(28)-C(26)-C(25)	120.17	N(34)-C(35)-C(33)	122.24
N(27)-C(28)-C(26)	124.20	O(39)-C(25)-C(26)	121.94	C(42)-N(34)-C(35)	118.38
C(35)-N(27)-C(28)	108.57	O(39)-C(25)-C(19)	119.69	C(42)-N(34)-C(27)	121.88
C(35)-N(27)-C(20)	108.04	C(26)-C(25)-C(19)	118.37	C(35)-N(34)-C(27)	119.36
C(28)-N(27)-C(20)	114.33	N(29)-C(24)-C(23)	120.65	C(47)-C(33)-C(35)	120.14
C(40)-C(26)-C(28)	117.50	N(29)-C(24)-C(22)	119.89	C(47)-C(33)-C(32)	119.28
C(40)-C(26)-C(25)	122.38	C(23)-C(24)-C(22)	119.46	C(35)-C(33)-C(32)	120.42
C(28)-C(26)-C(25)	120.13	F(38)-C(23)-C(24)	120.68	O(46)-C(32)-C(33)	123.58
O(39)-C(25)-C(26)	122.63	F(38)-C(23)-C(21)	119.09	O(46)-C(32)-C(26)	118.12
O(39)-C(25)-C(19)	120.17	C(24)-C(23)-C(21)	120.21	C(33)-C(32)-C(26)	118.30
C(26)-C(25)-C(19)	117.19	H(44)-C(22)-C(24)	118.37	N(36)-C(31)-C(30)	120.69
N(29)-C(24)-C(23)	119.91	H(44)-C(22)-C(20)	120.96	N(36)-C(31)-C(29)	119.91
N(29)-C(24)-C(22)	120.07	C(24)-C(22)-C(20)	120.66	C(30)-C(31)-C(29)	119.40
C(23)-C(24)-C(22)	120.01	H(43)-C(21)-C(23)	118.84	F(45)-C(30)-C(31)	120.68
F(38)-C(23)-C(24)	120.09	H(43)-C(21)-C(19)	120.98	F(45)-C(30)-C(28)	119.10
F(38)-C(23)-C(21)	120.10	C(23)-C(21)-C(19)	120.18	C(31)-C(30)-C(28)	120.21
C(24)-C(23)-C(21)	119.81	N(27)-C(20)-C(22)	122.40	H(51)-C(29)-C(31)	118.34
H(44)-C(22)-C(24)	119.99	N(27)-C(20)-C(19)	118.38	H(51)-C(29)-C(27)	120.96
H(44)-C(22)-C(20)	119.99	C(22)-C(20)-C(19)	119.22	C(31)-C(29)-C(27)	120.69
C(24)-C(22)-C(20)	120.02	C(25)-C(19)-C(21)	122.25	H(50)-C(28)-C(30)	118.63
H(43)-C(21)-C(23)	120.22	C(25)-C(19)-C(20)	117.48	H(50)-C(28)-C(26)	121.01
H(43)-C(21)-C(19)	120.23	C(21)-C(19)-C(20)	120.25	C(30)-C(28)-C(26)	120.35
C(23)-C(21)-C(19)	119.55	H(18)-C(10)-H(17)	108.23	N(34)-C(27)-C(29)	122.31
N(27)-C(20)-C(22)	119.46	H(18)-C(10)-H(16)	108.82	N(34)-C(27)-C(26)	118.31
N(27)-C(20)-C(19)	121.08	H(18)-C(10)-C(8)	110.00	C(29)-C(27)-C(26)	119.38
C(22)-C(20)-C(19)	119.45	H(17)-C(10)-H(16)	109.73	C(32)-C(26)-C(28)	121.98
C(25)-C(19)-C(21)	121.18	H(17)-C(10)-C(8)	110.23	C(32)-C(26)-C(27)	118.06
C(25)-C(19)-C(20)	117.93	H(16)-C(10)-C(8)	109.79	C(28)-C(26)-C(27)	119.96
C(21)-C(19)-C(20)	120.88	H(15)-N(9)-C(8)	127.40	H(24)-C(16)-H(23)	111.64
H(18)-C(10)-H(17)	109.48	H(15)-N(9)-C(2)	127.08	H(24)-C(16)-H(22)	105.63
H(18)-C(10)-H(16)	109.47	C(8)-N(9)-C(2)	101.80	H(24)-C(16)-C(14)	111.34
H(18)-C(10)-C(8)	109.46	C(10)-C(8)-N(9)	122.97	H(23)-C(16)-H(22)	105.79
H(17)-C(10)-H(16)	109.48	C(10)-C(8)-N(7)	123.44	H(23)-C(16)-C(14)	113.00
H(17)-C(10)-C(8)	109.46	N(9)-C(8)-N(7)	113.33	H(22)-C(16)-C(14)	108.99
H(16)-C(10)-C(8)	109.48	Ni(63)-N(7)-C(8)	124.82	H(21)-N(15)-C(14)	128.36

H(15)-N(9)-C(8)	113.82	Ni(63)-N(7)-C(1)	129.48	H(21)-N(15)-C(8)	127.62
H(15)-N(9)-C(2)	113.65	C(8)-N(7)-C(1)	105.11	C(14)-N(15)-C(8)	102.72
C(8)-N(9)-C(2)	101.13	H(14)-C(6)-C(5)	119.69	C(16)-C(14)-N(15)	119.00
C(10)-C(8)-N(9)	124.83	H(14)-C(6)-C(4)	119.99	C(16)-C(14)-N(13)	127.60
C(10)-C(8)-N(7)	124.77	C(5)-C(6)-C(4)	120.30	N(15)-C(14)-N(13)	113.33
N(9)-C(8)-N(7)	110.40	H(13)-C(5)-C(6)	119.54	Ni(67)-N(13)-C(14)	131.53
Ni(67)-N(7)-C(8)	125.98	H(13)-C(5)-C(3)	119.92	Ni(67)-N(13)-C(7)	124.97
Ni(67)-N(7)-C(1)	126.02	C(6)-C(5)-C(3)	120.52	C(14)-N(13)-C(7)	103.49
C(8)-N(7)-C(1)	107.99	H(12)-C(4)-C(6)	121.00	H(20)-C(12)-C(11)	119.79
H(14)-C(6)-C(5)	119.82	H(12)-C(4)-C(2)	120.86	H(20)-C(12)-C(10)	120.07
H(14)-C(6)-C(4)	119.81	C(6)-C(4)-C(2)	118.13	C(11)-C(12)-C(10)	120.14
C(5)-C(6)-C(4)	120.37	H(11)-C(3)-C(5)	119.56	H(19)-C(11)-C(12)	119.50
H(13)-C(5)-C(6)	119.87	H(11)-C(3)-C(1)	122.07	H(19)-C(11)-C(9)	119.93
H(13)-C(5)-C(3)	119.86	C(5)-C(3)-C(1)	118.34	C(12)-C(11)-C(9)	120.57
C(6)-C(5)-C(3)	120.27	N(9)-C(2)-C(4)	127.32	H(18)-C(10)-C(12)	121.00
H(12)-C(4)-C(6)	121.08	N(9)-C(2)-C(1)	110.15	H(18)-C(10)-C(8)	120.90
H(12)-C(4)-C(2)	121.07	C(4)-C(2)-C(1)	122.02	C(12)-C(10)-C(8)	118.10
C(6)-C(4)-C(2)	117.85	N(7)-C(1)-C(3)	129.07	H(17)-C(9)-C(11)	118.48
H(11)-C(3)-C(5)	121.15	N(7)-C(1)-C(2)	109.49	H(17)-C(9)-C(7)	122.76
H(11)-C(3)-C(1)	121.15	C(3)-C(1)-C(2)	120.62	C(11)-C(9)-C(7)	118.77
C(5)-C(3)-C(1)	117.70	H(61)-O(60)-Ni(63)-N(7)	160.60	N(15)-C(8)-C(10)	127.15
N(9)-C(2)-C(4)	128.79	H(61)-O(60)-Ni(63)-O(39)	36.92	N(15)-C(8)-C(7)	109.96
N(9)-C(2)-C(1)	109.71	H(61)-O(60)-Ni(63)-O(42)	-70.99	C(10)-C(8)-C(7)	122.84
C(4)-C(2)-C(1)	121.49	H(62)-O(60)-Ni(63)-N(7)	-89.99	N(13)-C(7)-C(9)	129.88
N(7)-C(1)-C(3)	129.39	H(62)-O(60)-Ni(63)-O(39)	146.33	N(13)-C(7)-C(8)	110.46
N(7)-C(1)-C(2)	108.56	H(62)-O(60)-Ni(63)-O(42)	38.41	C(9)-C(7)-C(8)	119.58
C(3)-C(1)-C(2)	122.04	C(40)-O(42)-Ni(63)-N(7)	179.65	Ni(67)-O(4)-H(6)	108.04
C(60)-O(63)-Ni(67)-N(7)	-50.21	C(40)-O(42)-Ni(63)-O(39)	-67.37	Ni(67)-O(4)-H(5)	110.56
C(60)-O(63)-Ni(67)-O(39)	70.81	C(40)-O(42)-Ni(63)-O(60)	51.17	H(6)-O(4)-H(5)	102.78
C(60)-O(63)-Ni(67)-O(42)	-171.02	C(26)-C(40)-O(42)-Ni(63)	40.45	Ni(67)-O(1)-H(3)	110.80
C(61)-C(60)-O(63)-Ni(67)	-150.43	O(41)-C(40)-O(42)-Ni(63)	-153.64	Ni(67)-O(1)-H(2)	109.26
O(62)-C(60)-O(63)-Ni(67)	29.57	C(25)-O(39)-Ni(63)-N(7)	166.35	H(3)-O(1)-H(2)	107.96
O(62)-C(60)-C(61)-H(64)	-150.08	C(25)-O(39)-Ni(63)-O(42)	55.30	C(47)-O(49)-Ni(67)-O(1)	-140.73
O(62)-C(60)-C(61)-H(65)	-30.06	C(25)-O(39)-Ni(63)-O(60)	-71.09	C(47)-O(49)-Ni(67)-O(4)	58.70
O(62)-C(60)-C(61)-H(66)	89.93	C(35)-C(36)-C(37)-H(58)	-107.03	C(47)-O(49)-Ni(67)-N(13)	147.73
O(63)-C(60)-C(61)-H(64)	29.92	C(35)-C(36)-C(37)-H(59)	105.23	C(47)-O(49)-Ni(67)-Cl(25)	-120.04
O(63)-C(60)-C(61)-H(65)	149.94	H(56)-C(36)-C(37)-C(35)	-105.51	C(47)-O(49)-Ni(67)-O(46)	-33.23
O(63)-C(60)-C(61)-H(66)	-90.07	H(56)-C(36)-C(37)-H(58)	147.46	C(33)-C(47)-O(49)-Ni(67)	17.64
C(40)-O(42)-Ni(67)-N(7)	155.32	H(56)-C(36)-C(37)-H(59)	-0.29	O(48)-C(47)-O(49)-Ni(67)	-167.29
C(40)-O(42)-Ni(67)-O(39)	44.35	H(57)-C(36)-C(37)-C(35)	107.89	C(32)-O(46)-Ni(67)-O(1)	-152.93
C(40)-O(42)-Ni(67)-O(63)	-75.06	H(57)-C(36)-C(37)-H(58)	0.86	C(32)-O(46)-Ni(67)-O(4)	-65.09
C(26)-C(40)-O(42)-Ni(67)	-31.08	H(57)-C(36)-C(37)-H(59)	-146.89	C(32)-O(46)-Ni(67)-N(13)	-177.09
O(41)-C(40)-O(42)-Ni(67)	149.66	N(27)-C(35)-C(37)-C(36)	-105.69	C(32)-O(46)-Ni(67)-Cl(25)	113.78
C(25)-O(39)-Ni(67)-N(7)	-151.55	N(27)-C(35)-C(37)-H(58)	-1.60	C(32)-O(46)-Ni(67)-O(49)	28.51

C(25)-O(39)-Ni(67)-O(42)	-40.92	N(27)-C(35)-C(37)-H(59)	149.29	C(42)-C(43)-C(44)-H(65)	-107.14
C(25)-O(39)-Ni(67)-O(63)	78.84	C(36)-C(35)-C(37)-H(58)	104.09	C(42)-C(43)-C(44)-H(66)	105.31
C(35)-C(36)-C(37)-H(58)	-106.87	C(36)-C(35)-C(37)-H(59)	-105.01	H(63)-C(43)-C(44)-C(42)	-105.56
C(35)-C(36)-C(37)-H(59)	108.07	H(55)-C(35)-C(37)-C(36)	109.21	H(63)-C(43)-C(44)-H(65)	147.29
H(56)-C(36)-C(37)-C(35)	-111.28	H(55)-C(35)-C(37)-H(58)	-146.69	H(63)-C(43)-C(44)-H(66)	-0.25
H(56)-C(36)-C(37)-H(58)	141.85	H(55)-C(35)-C(37)-H(59)	4.20	H(64)-C(43)-C(44)-C(42)	108.03
H(56)-C(36)-C(37)-H(59)	-3.21	N(27)-C(35)-C(36)-C(37)	104.02	H(64)-C(43)-C(44)-H(65)	0.89
H(57)-C(36)-C(37)-C(35)	110.64	N(27)-C(35)-C(36)-H(56)	-151.34	H(64)-C(43)-C(44)-H(66)	-146.65
H(57)-C(36)-C(37)-H(58)	3.77	N(27)-C(35)-C(36)-H(57)	0.77	N(34)-C(42)-C(44)-C(43)	-105.94
H(57)-C(36)-C(37)-H(59)	-141.29	C(37)-C(35)-C(36)-H(56)	104.64	N(34)-C(42)-C(44)-H(65)	-1.96
N(27)-C(35)-C(37)-C(36)	-107.05	C(37)-C(35)-C(36)-H(57)	-103.25	N(34)-C(42)-C(44)-H(66)	149.11
N(27)-C(35)-C(37)-H(58)	6.12	H(55)-C(35)-C(36)-C(37)	-106.19	C(43)-C(42)-C(44)-H(65)	103.99
N(27)-C(35)-C(37)-H(59)	147.33	H(55)-C(35)-C(36)-H(56)	-1.56	C(43)-C(42)-C(44)-H(66)	-104.95
C(36)-C(35)-C(37)-H(58)	113.17	H(55)-C(35)-C(36)-H(57)	150.55	H(62)-C(42)-C(44)-C(43)	109.59
C(36)-C(35)-C(37)-H(59)	-105.61	C(31)-C(33)-N(34)-C(32)	-61.13	H(62)-C(42)-C(44)-H(65)	-146.43
H(55)-C(35)-C(37)-C(36)	112.46	C(31)-C(33)-N(34)-H(54)	-178.65	H(62)-C(42)-C(44)-H(66)	4.64
H(55)-C(35)-C(37)-H(58)	-134.38	H(52)-C(33)-N(34)-C(32)	179.07	N(34)-C(42)-C(43)-C(44)	103.81
H(55)-C(35)-C(37)-H(59)	6.84	H(52)-C(33)-N(34)-H(54)	61.55	N(34)-C(42)-C(43)-H(63)	-151.63
N(27)-C(35)-C(36)-C(37)	107.01	H(53)-C(33)-N(34)-C(32)	60.46	N(34)-C(42)-C(43)-H(64)	0.67
N(27)-C(35)-C(36)-H(56)	-148.07	H(53)-C(33)-N(34)-H(54)	-57.06	C(44)-C(42)-C(43)-H(63)	104.56
N(27)-C(35)-C(36)-H(57)	-6.03	C(30)-C(32)-N(34)-C(33)	61.39	C(44)-C(42)-C(43)-H(64)	-103.15
C(37)-C(35)-C(36)-H(56)	104.92	C(30)-C(32)-N(34)-H(54)	178.91	H(62)-C(42)-C(43)-C(44)	-105.74
C(37)-C(35)-C(36)-H(57)	-113.04	H(50)-C(32)-N(34)-C(33)	-60.24	H(62)-C(42)-C(43)-H(63)	-1.17
H(55)-C(35)-C(36)-C(37)	-112.46	H(50)-C(32)-N(34)-H(54)	57.28	H(62)-C(42)-C(43)-H(64)	151.12
H(55)-C(35)-C(36)-H(56)	-7.54	H(51)-C(32)-N(34)-C(33)	-178.93	C(38)-C(40)-N(41)-C(39)	-61.10
H(55)-C(35)-C(36)-H(57)	134.50	H(51)-C(32)-N(34)-H(54)	-61.41	C(38)-C(40)-N(41)-H(61)	-178.61
C(31)-C(33)-N(34)-C(32)	-74.98	N(29)-C(31)-C(33)-N(34)	52.97	H(59)-C(40)-N(41)-C(39)	179.11
C(31)-C(33)-N(34)-H(54)	48.30	N(29)-C(31)-C(33)-H(52)	172.96	H(59)-C(40)-N(41)-H(61)	61.59
H(52)-C(33)-N(34)-C(32)	42.94	N(29)-C(31)-C(33)-H(53)	-69.00	H(60)-C(40)-N(41)-C(39)	60.51
H(52)-C(33)-N(34)-H(54)	166.22	H(48)-C(31)-C(33)-N(34)	-66.75	H(60)-C(40)-N(41)-H(61)	-57.01
H(53)-C(33)-N(34)-C(32)	162.53	H(48)-C(31)-C(33)-H(52)	53.24	C(37)-C(39)-N(41)-C(40)	61.33
H(53)-C(33)-N(34)-H(54)	-74.18	H(48)-C(31)-C(33)-H(53)	171.28	C(37)-C(39)-N(41)-H(61)	178.85
C(30)-C(32)-N(34)-C(33)	75.00	H(49)-C(31)-C(33)-N(34)	174.68	H(57)-C(39)-N(41)-C(40)	-179.00
C(30)-C(32)-N(34)-H(54)	-48.27	H(49)-C(31)-C(33)-H(52)	-65.33	H(57)-C(39)-N(41)-H(61)	-61.49
H(50)-C(32)-N(34)-C(33)	-42.90	H(49)-C(31)-C(33)-H(53)	52.70	H(58)-C(39)-N(41)-C(40)	-60.32
H(50)-C(32)-N(34)-H(54)	-166.17	N(29)-C(30)-C(32)-N(34)	-53.61	H(58)-C(39)-N(41)-H(61)	57.20
H(51)-C(32)-N(34)-C(33)	-162.50	N(29)-C(30)-C(32)-H(50)	68.40	N(36)-C(38)-C(40)-N(41)	53.00
H(51)-C(32)-N(34)-H(54)	74.23	N(29)-C(30)-C(32)-H(51)	-173.48	N(36)-C(38)-C(40)-H(59)	172.97
N(29)-C(31)-C(33)-N(34)	21.75	H(46)-C(30)-C(32)-N(34)	-176.17	N(36)-C(38)-C(40)-H(60)	-69.00
N(29)-C(31)-C(33)-H(52)	-92.53	H(46)-C(30)-C(32)-H(50)	-54.16	H(55)-C(38)-C(40)-N(41)	-66.72
N(29)-C(31)-C(33)-H(53)	147.53	H(46)-C(30)-C(32)-H(51)	63.96	H(55)-C(38)-C(40)-H(59)	53.26
H(48)-C(31)-C(33)-N(34)	-92.84	H(47)-C(30)-C(32)-N(34)	65.71	H(55)-C(38)-C(40)-H(60)	171.29
H(48)-C(31)-C(33)-H(52)	152.88	H(47)-C(30)-C(32)-H(50)	-172.28	H(56)-C(38)-C(40)-N(41)	174.71
H(48)-C(31)-C(33)-H(53)	32.94	H(47)-C(30)-C(32)-H(51)	-54.17	H(56)-C(38)-C(40)-H(59)	-65.31

H(49)-C(31)-C(33)-N(34)	146.95	C(24)-N(29)-C(31)-C(33)	121.36	H(56)-C(38)-C(40)-H(60)	52.72
H(49)-C(31)-C(33)-H(52)	32.66	C(24)-N(29)-C(31)-H(48)	-118.68	N(36)-C(37)-C(39)-N(41)	-53.58
H(49)-C(31)-C(33)-H(53)	-87.28	C(24)-N(29)-C(31)-H(49)	0.88	N(36)-C(37)-C(39)-H(57)	-173.44
N(29)-C(30)-C(32)-N(34)	-21.77	C(30)-N(29)-C(31)-C(33)	-49.90	N(36)-C(37)-C(39)-H(58)	68.45
N(29)-C(30)-C(32)-H(50)	92.52	C(30)-N(29)-C(31)-H(48)	70.05	H(53)-C(37)-C(39)-N(41)	-176.15
N(29)-C(30)-C(32)-H(51)	-147.56	C(30)-N(29)-C(31)-H(49)	-170.38	H(53)-C(37)-C(39)-H(57)	63.99
H(46)-C(30)-C(32)-N(34)	-146.93	C(24)-N(29)-C(30)-C(32)	-120.97	H(53)-C(37)-C(39)-H(58)	-54.13
H(46)-C(30)-C(32)-H(50)	-32.64	C(24)-N(29)-C(30)-H(46)	0.64	H(54)-C(37)-C(39)-N(41)	65.75
H(46)-C(30)-C(32)-H(51)	87.29	C(24)-N(29)-C(30)-H(47)	119.50	H(54)-C(37)-C(39)-H(57)	-54.11
H(47)-C(30)-C(32)-N(34)	92.84	C(31)-N(29)-C(30)-C(32)	50.29	H(54)-C(37)-C(39)-H(58)	-172.22
H(47)-C(30)-C(32)-H(50)	-152.87	C(31)-N(29)-C(30)-H(46)	171.90	C(31)-N(36)-C(38)-C(40)	121.61
H(47)-C(30)-C(32)-H(51)	-32.95	C(31)-N(29)-C(30)-H(47)	-69.24	C(31)-N(36)-C(38)-H(55)	-118.44
C(24)-N(29)-C(31)-C(33)	163.65	C(20)-N(27)-C(35)-C(36)	79.47	C(31)-N(36)-C(38)-H(56)	1.12
C(24)-N(29)-C(31)-H(48)	-77.66	C(20)-N(27)-C(35)-C(37)	145.54	C(37)-N(36)-C(38)-C(40)	-49.94
C(24)-N(29)-C(31)-H(49)	42.27	C(20)-N(27)-C(35)-H(55)	-70.20	C(37)-N(36)-C(38)-H(55)	70.01
C(30)-N(29)-C(31)-C(33)	38.55	C(28)-N(27)-C(35)-C(36)	-91.23	C(37)-N(36)-C(38)-H(56)	-170.43
C(30)-N(29)-C(31)-H(48)	157.23	C(28)-N(27)-C(35)-C(37)	-25.16	C(31)-N(36)-C(37)-C(39)	-121.25
C(30)-N(29)-C(31)-H(49)	-82.84	C(28)-N(27)-C(35)-H(55)	119.10	C(31)-N(36)-C(37)-H(53)	0.37
C(24)-N(29)-C(30)-C(32)	-163.58	C(20)-N(27)-C(28)-C(26)	16.60	C(31)-N(36)-C(37)-H(54)	119.22
C(24)-N(29)-C(30)-H(46)	-42.23	C(20)-N(27)-C(28)-H(45)	-161.51	C(38)-N(36)-C(37)-C(39)	50.30
C(24)-N(29)-C(30)-H(47)	77.72	C(35)-N(27)-C(28)-C(26)	-172.46	C(38)-N(36)-C(37)-H(53)	171.91
C(31)-N(29)-C(30)-C(32)	-38.53	C(35)-N(27)-C(28)-H(45)	9.43	C(38)-N(36)-C(37)-H(54)	-69.24
C(31)-N(29)-C(30)-H(46)	82.82	C(25)-C(26)-C(40)-O(41)	-153.65	C(27)-N(34)-C(42)-C(43)	-131.61
C(31)-N(29)-C(30)-H(47)	-157.24	C(25)-C(26)-C(40)-O(42)	12.03	C(27)-N(34)-C(42)-C(44)	-65.56
C(20)-N(27)-C(35)-C(36)	-152.41	C(28)-C(26)-C(40)-O(41)	15.40	C(27)-N(34)-C(42)-H(62)	77.97
C(20)-N(27)-C(35)-C(37)	-83.64	C(28)-C(26)-C(40)-O(42)	-178.92	C(35)-N(34)-C(42)-C(43)	55.59
C(20)-N(27)-C(35)-H(55)	61.96	C(25)-C(26)-C(28)-N(27)	0.66	C(35)-N(34)-C(42)-C(44)	121.65
C(28)-N(27)-C(35)-C(36)	83.07	C(25)-C(26)-C(28)-H(45)	178.76	C(35)-N(34)-C(42)-H(62)	-94.83
C(28)-N(27)-C(35)-C(37)	151.84	C(40)-C(26)-C(28)-N(27)	-168.17	C(27)-N(34)-C(35)-C(33)	15.09
C(28)-N(27)-C(35)-H(55)	-62.56	C(40)-C(26)-C(28)-H(45)	9.93	C(27)-N(34)-C(35)-H(52)	-165.29
C(20)-N(27)-C(28)-C(26)	27.50	C(19)-C(25)-O(39)-Ni(63)	166.78	C(42)-N(34)-C(35)-C(33)	-171.93
C(20)-N(27)-C(28)-H(45)	-153.77	C(26)-C(25)-O(39)-Ni(63)	-13.40	C(42)-N(34)-C(35)-H(52)	7.69
C(35)-N(27)-C(28)-C(26)	148.21	C(19)-C(25)-C(26)-C(28)	-17.80	C(32)-C(33)-C(47)-O(48)	-161.57
C(35)-N(27)-C(28)-H(45)	-33.07	C(19)-C(25)-C(26)-C(40)	151.31	C(32)-C(33)-C(47)-O(49)	13.40
C(25)-C(26)-C(40)-O(41)	-173.51	O(39)-C(25)-C(26)-C(28)	162.38	C(35)-C(33)-C(47)-O(48)	13.92
C(25)-C(26)-C(40)-O(42)	7.23	O(39)-C(25)-C(26)-C(40)	-28.51	C(35)-C(33)-C(47)-O(49)	-171.11
C(28)-C(26)-C(40)-O(41)	6.56	C(22)-C(24)-N(29)-C(30)	49.13	C(32)-C(33)-C(35)-N(34)	0.11
C(28)-C(26)-C(40)-O(42)	-172.71	C(22)-C(24)-N(29)-C(31)	-121.85	C(32)-C(33)-C(35)-H(52)	-179.50
C(25)-C(26)-C(28)-N(27)	-15.91	C(23)-C(24)-N(29)-C(30)	-130.60	C(47)-C(33)-C(35)-N(34)	-175.32
C(25)-C(26)-C(28)-H(45)	165.37	C(23)-C(24)-N(29)-C(31)	58.42	C(47)-C(33)-C(35)-H(52)	5.06
C(40)-C(26)-C(28)-N(27)	164.03	C(21)-C(23)-C(24)-C(22)	-0.16	C(26)-C(32)-O(46)-Ni(67)	173.15
C(40)-C(26)-C(28)-H(45)	-14.70	C(21)-C(23)-C(24)-N(29)	179.57	C(33)-C(32)-O(46)-Ni(67)	-6.09
C(19)-C(25)-O(39)-Ni(67)	-159.37	F(38)-C(23)-C(24)-C(22)	-178.62	C(26)-C(32)-C(33)-C(35)	-14.80
C(26)-C(25)-O(39)-Ni(67)	18.97	F(38)-C(23)-C(24)-N(29)	1.11	C(26)-C(32)-C(33)-C(47)	160.67

C(19)-C(25)-C(26)-C(28)	-0.84	C(20)-C(22)-C(24)-C(23)	0.70	O(46)-C(32)-C(33)-C(35)	164.44
C(19)-C(25)-C(26)-C(40)	179.22	C(20)-C(22)-C(24)-N(29)	-179.03	O(46)-C(32)-C(33)-C(47)	-20.09
O(39)-C(25)-C(26)-C(28)	-179.23	H(44)-C(22)-C(24)-C(23)	-178.17	C(29)-C(31)-N(36)-C(37)	48.89
O(39)-C(25)-C(26)-C(40)	0.84	H(44)-C(22)-C(24)-N(29)	2.10	C(29)-C(31)-N(36)-C(38)	-122.38
C(22)-C(24)-N(29)-C(30)	-25.12	C(19)-C(21)-C(23)-C(24)	0.22	C(30)-C(31)-N(36)-C(37)	-130.63
C(22)-C(24)-N(29)-C(31)	-155.21	C(19)-C(21)-C(23)-F(38)	178.71	C(30)-C(31)-N(36)-C(38)	58.09
C(23)-C(24)-N(29)-C(30)	155.03	H(43)-C(21)-C(23)-C(24)	179.86	C(28)-C(30)-C(31)-C(29)	0.03
C(23)-C(24)-N(29)-C(31)	24.93	H(43)-C(21)-C(23)-F(38)	-1.66	C(28)-C(30)-C(31)-N(36)	179.56
C(21)-C(23)-C(24)-C(22)	2.25	C(19)-C(20)-N(27)-C(28)	-15.99	F(45)-C(30)-C(31)-C(29)	-178.46
C(21)-C(23)-C(24)-N(29)	-177.89	C(19)-C(20)-N(27)-C(35)	173.41	F(45)-C(30)-C(31)-N(36)	1.07
F(38)-C(23)-C(24)-C(22)	-177.97	C(22)-C(20)-N(27)-C(28)	163.42	C(27)-C(29)-C(31)-C(30)	0.84
F(38)-C(23)-C(24)-N(29)	1.89	C(22)-C(20)-N(27)-C(35)	-7.18	C(27)-C(29)-C(31)-N(36)	-178.69
C(20)-C(22)-C(24)-C(23)	-0.20	C(19)-C(20)-C(22)-C(24)	-1.23	H(51)-C(29)-C(31)-C(30)	-178.27
C(20)-C(22)-C(24)-N(29)	179.94	C(19)-C(20)-C(22)-H(44)	177.61	H(51)-C(29)-C(31)-N(36)	2.20
H(44)-C(22)-C(24)-C(23)	179.41	N(27)-C(20)-C(22)-C(24)	179.36	C(26)-C(28)-C(30)-C(31)	-0.07
H(44)-C(22)-C(24)-N(29)	-0.45	N(27)-C(20)-C(22)-H(44)	-1.80	C(26)-C(28)-C(30)-F(45)	178.44
C(19)-C(21)-C(23)-C(24)	0.13	C(20)-C(19)-C(25)-C(26)	17.35	H(50)-C(28)-C(30)-C(31)	179.59
C(19)-C(21)-C(23)-F(38)	-179.65	C(20)-C(19)-C(25)-O(39)	-162.83	H(50)-C(28)-C(30)-F(45)	-1.90
H(43)-C(21)-C(23)-C(24)	179.64	C(21)-C(19)-C(25)-C(26)	-164.24	C(26)-C(27)-N(34)-C(35)	-14.75
H(43)-C(21)-C(23)-F(38)	-0.14	C(21)-C(19)-C(25)-O(39)	15.59	C(26)-C(27)-N(34)-C(42)	172.52
C(19)-C(20)-N(27)-C(28)	-22.74	C(20)-C(19)-C(21)-C(23)	-0.76	C(29)-C(27)-N(34)-C(35)	164.87
C(19)-C(20)-N(27)-C(35)	-143.73	C(20)-C(19)-C(21)-H(43)	179.61	C(29)-C(27)-N(34)-C(42)	-7.86
C(22)-C(20)-N(27)-C(28)	157.97	C(25)-C(19)-C(21)-C(23)	-179.13	C(26)-C(27)-C(29)-C(31)	-1.58
C(22)-C(20)-N(27)-C(35)	36.98	C(25)-C(19)-C(21)-H(43)	1.24	C(26)-C(27)-C(29)-H(51)	177.51
C(19)-C(20)-C(22)-C(24)	-3.96	C(21)-C(19)-C(20)-C(22)	1.29	N(34)-C(27)-C(29)-C(31)	178.81
C(19)-C(20)-C(22)-H(44)	176.42	C(21)-C(19)-C(20)-N(27)	-179.28	N(34)-C(27)-C(29)-H(51)	-2.11
N(27)-C(20)-C(22)-C(24)	175.34	C(25)-C(19)-C(20)-C(22)	179.74	C(27)-C(26)-C(32)-C(33)	14.20
N(27)-C(20)-C(22)-H(44)	-4.27	C(25)-C(19)-C(20)-N(27)	-0.83	C(27)-C(26)-C(32)-O(46)	-165.08
C(20)-C(19)-C(25)-C(26)	4.27	N(7)-C(8)-C(10)-H(16)	-49.49	C(28)-C(26)-C(32)-C(33)	-166.68
C(20)-C(19)-C(25)-O(39)	-177.30	N(7)-C(8)-C(10)-H(17)	71.50	C(28)-C(26)-C(32)-O(46)	14.04
C(21)-C(19)-C(25)-C(26)	-174.61	N(7)-C(8)-C(10)-H(18)	-169.22	C(27)-C(26)-C(28)-C(30)	-0.68
C(21)-C(19)-C(25)-O(39)	3.81	N(9)-C(8)-C(10)-H(16)	124.24	C(27)-C(26)-C(28)-H(50)	179.67
C(20)-C(19)-C(21)-C(23)	-4.37	N(9)-C(8)-C(10)-H(17)	-114.77	C(32)-C(26)-C(28)-C(30)	-179.77
C(20)-C(19)-C(21)-H(43)	176.11	N(9)-C(8)-C(10)-H(18)	4.51	C(32)-C(26)-C(28)-H(50)	0.57
C(25)-C(19)-C(21)-C(23)	174.48	N(7)-C(8)-N(9)-C(2)	3.21	C(28)-C(26)-C(27)-C(29)	1.52
C(25)-C(19)-C(21)-H(43)	-5.04	N(7)-C(8)-N(9)-H(15)	162.46	C(28)-C(26)-C(27)-N(34)	-178.85
C(21)-C(19)-C(20)-C(22)	6.41	C(10)-C(8)-N(9)-C(2)	-171.10	C(32)-C(26)-C(27)-C(29)	-179.34
C(21)-C(19)-C(20)-N(27)	-172.88	C(10)-C(8)-N(9)-H(15)	-11.85	C(32)-C(26)-C(27)-N(34)	0.28
C(25)-C(19)-C(20)-C(22)	-172.48	C(1)-N(7)-Ni(63)-O(39)	36.40	N(13)-C(14)-C(16)-H(22)	-174.57
C(25)-C(19)-C(20)-N(27)	8.23	C(1)-N(7)-Ni(63)-O(42)	136.16	N(13)-C(14)-C(16)-H(23)	-57.27
N(7)-C(8)-C(10)-H(16)	-90.11	C(1)-N(7)-Ni(63)-O(60)	-88.27	N(13)-C(14)-C(16)-H(24)	69.31
N(7)-C(8)-C(10)-H(17)	29.89	C(8)-N(7)-Ni(63)-O(39)	-153.70	N(15)-C(14)-C(16)-H(22)	8.62
N(7)-C(8)-C(10)-H(18)	149.89	C(8)-N(7)-Ni(63)-O(42)	-53.93	N(15)-C(14)-C(16)-H(23)	125.91
N(9)-C(8)-C(10)-H(16)	90.27	C(8)-N(7)-Ni(63)-O(60)	81.64	N(15)-C(14)-C(16)-H(24)	-107.51

N(9)-C(8)-C(10)-H(17)	-149.72	C(1)-N(7)-C(8)-N(9)	-3.47	N(13)-C(14)-N(15)-C(8)	-1.70
N(9)-C(8)-C(10)-H(18)	-29.72	C(1)-N(7)-C(8)-C(10)	170.81	N(13)-C(14)-N(15)-H(21)	-169.36
N(7)-C(8)-N(9)-C(2)	-14.83	Ni(63)-N(7)-C(8)-N(9)	-175.41	C(16)-C(14)-N(15)-C(8)	175.56
N(7)-C(8)-N(9)-H(15)	-137.09	Ni(63)-N(7)-C(8)-C(10)	-1.14	C(16)-C(14)-N(15)-H(21)	7.89
C(10)-C(8)-N(9)-C(2)	164.84	C(3)-C(5)-C(6)-C(4)	-0.72	C(7)-N(13)-Ni(67)-O(1)	137.60
C(10)-C(8)-N(9)-H(15)	42.57	C(3)-C(5)-C(6)-H(14)	-179.32	C(7)-N(13)-Ni(67)-O(4)	49.66
C(1)-N(7)-Ni(67)-O(39)	161.10	H(13)-C(5)-C(6)-C(4)	177.62	C(7)-N(13)-Ni(67)-Cl(25)	-129.25
C(1)-N(7)-Ni(67)-O(42)	48.15	H(13)-C(5)-C(6)-H(14)	-0.98	C(7)-N(13)-Ni(67)-O(46)	161.70
C(1)-N(7)-Ni(67)-O(63)	-75.53	C(2)-C(4)-C(6)-C(5)	1.49	C(7)-N(13)-Ni(67)-O(49)	-43.83
C(8)-N(7)-Ni(67)-O(39)	-17.67	C(2)-C(4)-C(6)-H(14)	-179.91	C(14)-N(13)-Ni(67)-O(1)	-44.11
C(8)-N(7)-Ni(67)-O(42)	-130.62	H(12)-C(4)-C(6)-C(5)	-178.32	C(14)-N(13)-Ni(67)-O(4)	-132.05
C(8)-N(7)-Ni(67)-O(63)	105.70	H(12)-C(4)-C(6)-H(14)	0.27	C(14)-N(13)-Ni(67)-Cl(25)	49.04
C(1)-N(7)-C(8)-N(9)	10.93	C(1)-C(3)-C(5)-C(6)	-1.43	C(14)-N(13)-Ni(67)-O(46)	-20.01
C(1)-N(7)-C(8)-C(10)	-168.73	C(1)-C(3)-C(5)-H(13)	-179.76	C(14)-N(13)-Ni(67)-O(49)	134.45
Ni(67)-N(7)-C(8)-N(9)	-170.12	H(11)-C(3)-C(5)-C(6)	176.57	C(7)-N(13)-C(14)-N(15)	1.46
Ni(67)-N(7)-C(8)-C(10)	10.22	H(11)-C(3)-C(5)-H(13)	-1.77	C(7)-N(13)-C(14)-C(16)	-175.51
C(3)-C(5)-C(6)-C(4)	-2.20	C(1)-C(2)-N(9)-C(8)	-1.54	Ni(67)-N(13)-C(14)-N(15)	-177.10
C(3)-C(5)-C(6)-H(14)	178.01	C(1)-C(2)-N(9)-H(15)	-160.88	Ni(67)-N(13)-C(14)-C(16)	5.93
H(13)-C(5)-C(6)-C(4)	178.03	C(4)-C(2)-N(9)-C(8)	170.28	C(9)-C(11)-C(12)-C(10)	-0.08
H(13)-C(5)-C(6)-H(14)	-1.76	C(4)-C(2)-N(9)-H(15)	10.94	C(9)-C(11)-C(12)-H(20)	179.61
C(2)-C(4)-C(6)-C(5)	-0.01	C(1)-C(2)-C(4)-C(6)	-0.03	H(19)-C(11)-C(12)-C(10)	-179.75
C(2)-C(4)-C(6)-H(14)	179.78	C(1)-C(2)-C(4)-H(12)	179.79	H(19)-C(11)-C(12)-H(20)	-0.06
H(12)-C(4)-C(6)-C(5)	-179.61	N(9)-C(2)-C(4)-C(6)	-170.96	C(8)-C(10)-C(12)-C(11)	-0.34
H(12)-C(4)-C(6)-H(14)	0.18	N(9)-C(2)-C(4)-H(12)	8.86	C(8)-C(10)-C(12)-H(20)	179.97
C(1)-C(3)-C(5)-C(6)	0.33	C(2)-C(1)-N(7)-C(8)	2.36	H(18)-C(10)-C(12)-C(11)	179.56
C(1)-C(3)-C(5)-H(13)	-179.90	C(2)-C(1)-N(7)-Ni(63)	173.79	H(18)-C(10)-C(12)-H(20)	-0.13
H(11)-C(3)-C(5)-C(6)	-179.24	C(3)-C(1)-N(7)-C(8)	-167.14	C(7)-C(9)-C(11)-C(12)	0.45
H(11)-C(3)-C(5)-H(13)	0.53	C(3)-C(1)-N(7)-Ni(63)	4.29	C(7)-C(9)-C(11)-H(19)	-179.89
C(1)-C(2)-N(9)-C(8)	13.35	C(2)-C(1)-C(3)-C(5)	2.90	H(17)-C(9)-C(11)-C(12)	-179.53
C(1)-C(2)-N(9)-H(15)	135.74	C(2)-C(1)-C(3)-H(11)	-175.05	H(17)-C(9)-C(11)-H(19)	0.13
C(4)-C(2)-N(9)-C(8)	-166.69	N(7)-C(1)-C(3)-C(5)	171.38	C(7)-C(8)-N(15)-C(14)	1.20
C(4)-C(2)-N(9)-H(15)	-44.31	N(7)-C(1)-C(3)-H(11)	-6.56	C(7)-C(8)-N(15)-H(21)	168.99
C(1)-C(2)-C(4)-C(6)	4.45	C(3)-C(1)-C(2)-C(4)	-2.18	C(10)-C(8)-N(15)-C(14)	-176.12
C(1)-C(2)-C(4)-H(12)	-175.95	C(3)-C(1)-C(2)-N(9)	170.15	C(10)-C(8)-N(15)-H(21)	-8.33
N(9)-C(2)-C(4)-C(6)	-175.50	N(7)-C(1)-C(2)-C(4)	-172.72	C(7)-C(8)-C(10)-C(12)	0.41
N(9)-C(2)-C(4)-H(12)	4.10	N(7)-C(1)-C(2)-N(9)	-0.39	C(7)-C(8)-C(10)-H(18)	-179.49
C(2)-C(1)-N(7)-C(8)	-2.01			N(15)-C(8)-C(10)-C(12)	177.42
C(2)-C(1)-N(7)-Ni(67)	179.04			N(15)-C(8)-C(10)-H(18)	-2.48
C(3)-C(1)-N(7)-C(8)	176.67			C(8)-C(7)-N(13)-C(14)	-0.64
C(3)-C(1)-N(7)-Ni(67)	-2.29			C(8)-C(7)-N(13)-Ni(67)	178.04
C(2)-C(1)-C(3)-C(5)	4.14			C(9)-C(7)-N(13)-C(14)	176.07
C(2)-C(1)-C(3)-H(11)	-176.29			C(9)-C(7)-N(13)-Ni(67)	-5.25
N(7)-C(1)-C(3)-C(5)	-174.38			C(8)-C(7)-C(9)-C(11)	-0.39
N(7)-C(1)-C(3)-H(11)	5.19			C(8)-C(7)-C(9)-H(17)	179.59

C(3)-C(1)-C(2)-C(4)	-6.57			N(13)-C(7)-C(9)-C(11)	-176.85
C(3)-C(1)-C(2)-N(9)	173.39			N(13)-C(7)-C(9)-H(17)	3.13
N(7)-C(1)-C(2)-C(4)	172.22			C(9)-C(7)-C(8)-C(10)	-0.04
N(7)-C(1)-C(2)-N(9)	-7.82			C(9)-C(7)-C(8)-N(15)	-177.51
				N(13)-C(7)-C(8)-C(10)	177.06
				N(13)-C(7)-C(8)-N(15)	-0.40
				H(5)-O(4)-Ni(67)-O(1)	146.68
				H(5)-O(4)-Ni(67)-N(13)	-120.97
				H(5)-O(4)-Ni(67)-Cl(25)	16.89
				H(5)-O(4)-Ni(67)-O(46)	61.09
				H(5)-O(4)-Ni(67)-O(49)	-33.83
				H(6)-O(4)-Ni(67)-O(1)	34.91
				H(6)-O(4)-Ni(67)-N(13)	127.27
				H(6)-O(4)-Ni(67)-Cl(25)	-94.87
				H(6)-O(4)-Ni(67)-O(46)	-50.67
				H(6)-O(4)-Ni(67)-O(49)	-145.59
				H(2)-O(1)-Ni(67)-O(4)	56.98
				H(2)-O(1)-Ni(67)-N(13)	-32.13
				H(2)-O(1)-Ni(67)-Cl(25)	-124.27
				H(2)-O(1)-Ni(67)-O(46)	148.77
				H(2)-O(1)-Ni(67)-O(49)	-103.61
				H(3)-O(1)-Ni(67)-O(4)	175.80
				H(3)-O(1)-Ni(67)-N(13)	86.68
				H(3)-O(1)-Ni(67)-Cl(25)	-5.45
				H(3)-O(1)-Ni(67)-O(46)	-92.41
				H(3)-O(1)-Ni(67)-O(49)	15.21

Table (S.8): Antibacterial activity as Activity index (%)

	Activity index (%)			
	Gram-positive bacteria		Gram-negative bacteria	
	B. cereus	*S. aureus*	*E. coli*	*P. aeruginosa*
MIM	22.86	23.53	33.33	30.00
CIP	28.57	29.41	36.67	33.33
NIAC	85.71	85.29	86.67	93.33
NIPE	82.86	79.41	83.33	90.00
NICL	74.29	76.47	80.00	83.33
Gentamicin	100	100	100	100

Table (S.9): Anti-fungal activity as Activity index (%)		
	Activity index (%)	
**Comp.**	*C. albicans*	*A. flavus*
MIM	40.00	44.44
CIP	50.00	50.00
NIAC	95.00	94.44
NIPE	90.00	94.44
NICL	80.00	83.33
clotrimazole	100	100

Table (S.10): Molecular docking interaction profile of the studied ligands (CIP, MIM, NIAC, NIPE, and NICL) with the target protein, showing interacting amino acid residues, intermolecular distances (Å), types of interactions (electrostatic, hydrogen bonding, halogen, and hydrophobic), and corresponding binding energies (kcal/mol).

	Residue	Distance	Interaction	Binding energy (kcal/mol)
CIP	LYS103	4.44	Electrostatic	-7.10
	ASP73	3.08	Halogen	
	ARG76	2.30	Hydrogen Bond	
	ASP73	2.21	Hydrogen Bond	
	LYS103	3.50	Hydrogen Bond	
	VAL43	2.88	Hydrogen Bond	
	ILE78	4.33	Hydrophobic	
	ILE94	4.30	Hydrophobic	
	LYS103	4.10	Hydrophobic	
MIM	ASP73	4.92	Electrostatic	-6.40
	ASP73	2.05	Hydrogen Bond	
	ILE78	3.55	Hydrophobic	
	VAL120	5.14	Hydrophobic	
NIAC	LYS103	4.69	Electrostatic	-8.50
	ALA100	2.88	Halogen	
	GLY101	3.09	Halogen	
	ALA100	2.80	Hydrogen Bond	
	ARG136	3.10	Hydrogen Bond	
	ARG76	2.28	Hydrogen Bond	
	ASN46	2.55	Hydrogen Bond	
	GLU50	2.69	Hydrogen Bond	
	GLY119	3.03	Hydrogen Bond	
	LYS103	2.61	Hydrogen Bond	
	ILE78	4.95	Hydrophobic	
	ILE94	4.80	Hydrophobic	
	LYS103	3.80	Hydrophobic	
NIPE	ASP49	5.48	Electrostatic	-8.10

	GLU50	4.20	Electrostatic	
	LYS103	4.43	Electrostatic	
	ARG136	2.19	Hydrogen Bond	
	GLY101	3.39	Hydrogen Bond	
	PRO79	3.44	Hydrogen Bond	
	HIS83	4.55	Hydrophobic	
	ILE78	4.52	Hydrophobic	
	PRO79	4.55	Hydrophobic	
NICL	GLU50	4.16	Electrostatic	-7.90
	LYS103	4.19	Electrostatic	
	ASN46	2.04	Hydrogen Bond	
	GLY101	2.53	Hydrogen Bond	
	HIS83	2.66	Hydrogen Bond	
	PRO79	2.48	Hydrogen Bond	
	ILE78	4.73	Hydrophobic	
	PRO79	4.36	Hydrophobic	

(a)

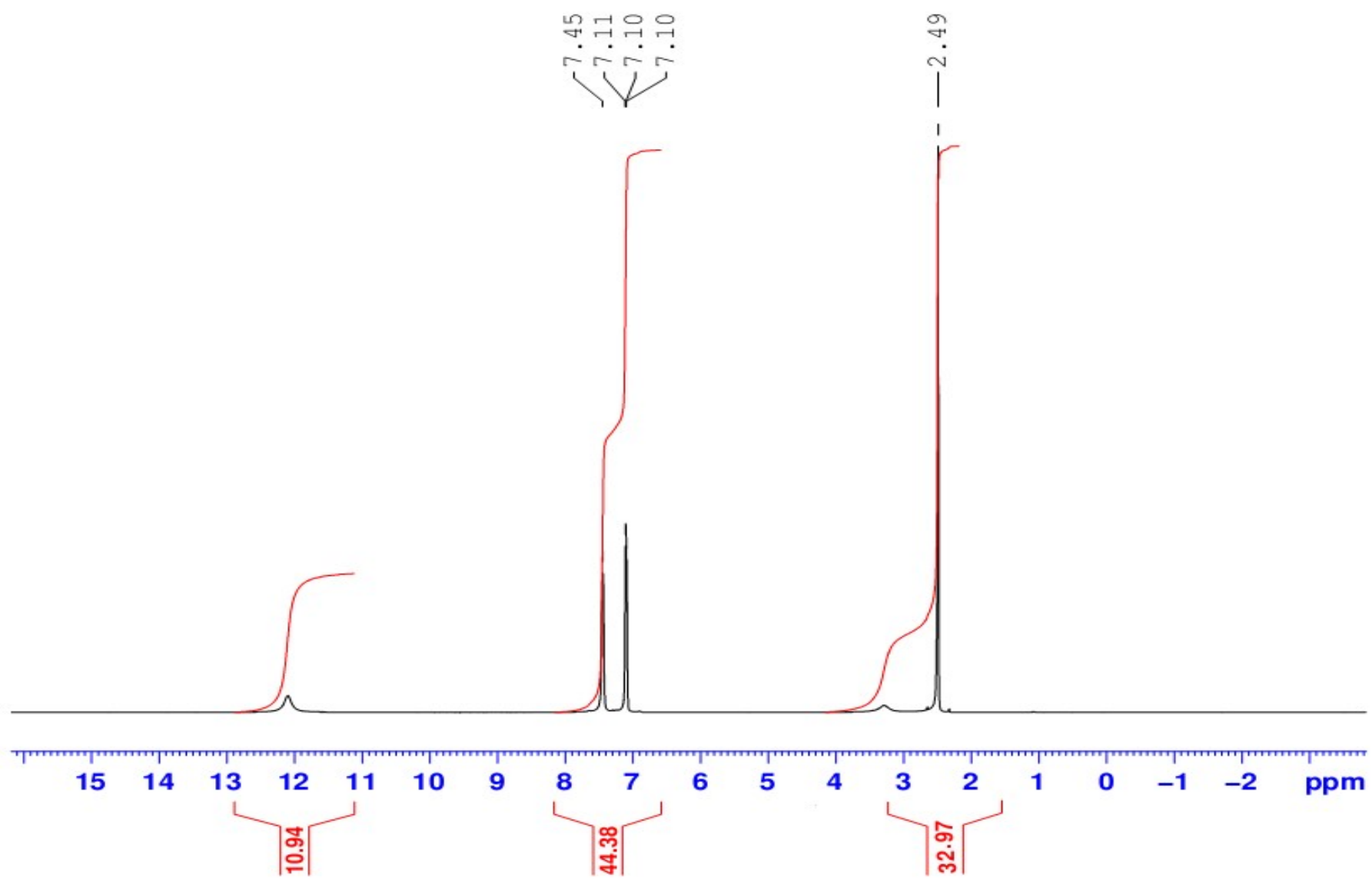

(b)

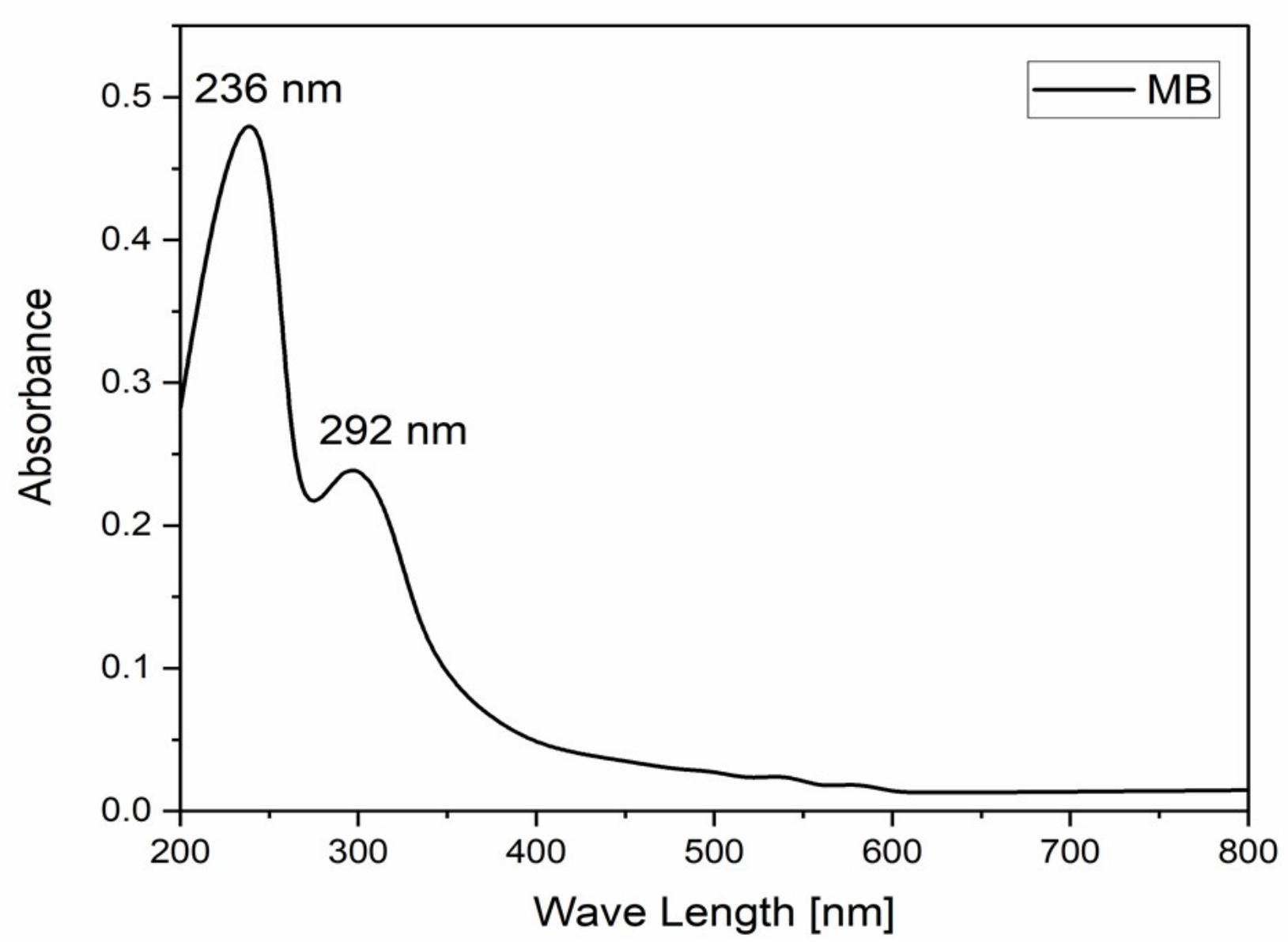

Fig. (S.1): The (a) ^{1}H-NMR spectrum, (b) UV-vis. spectra for the **MIM** ligand.

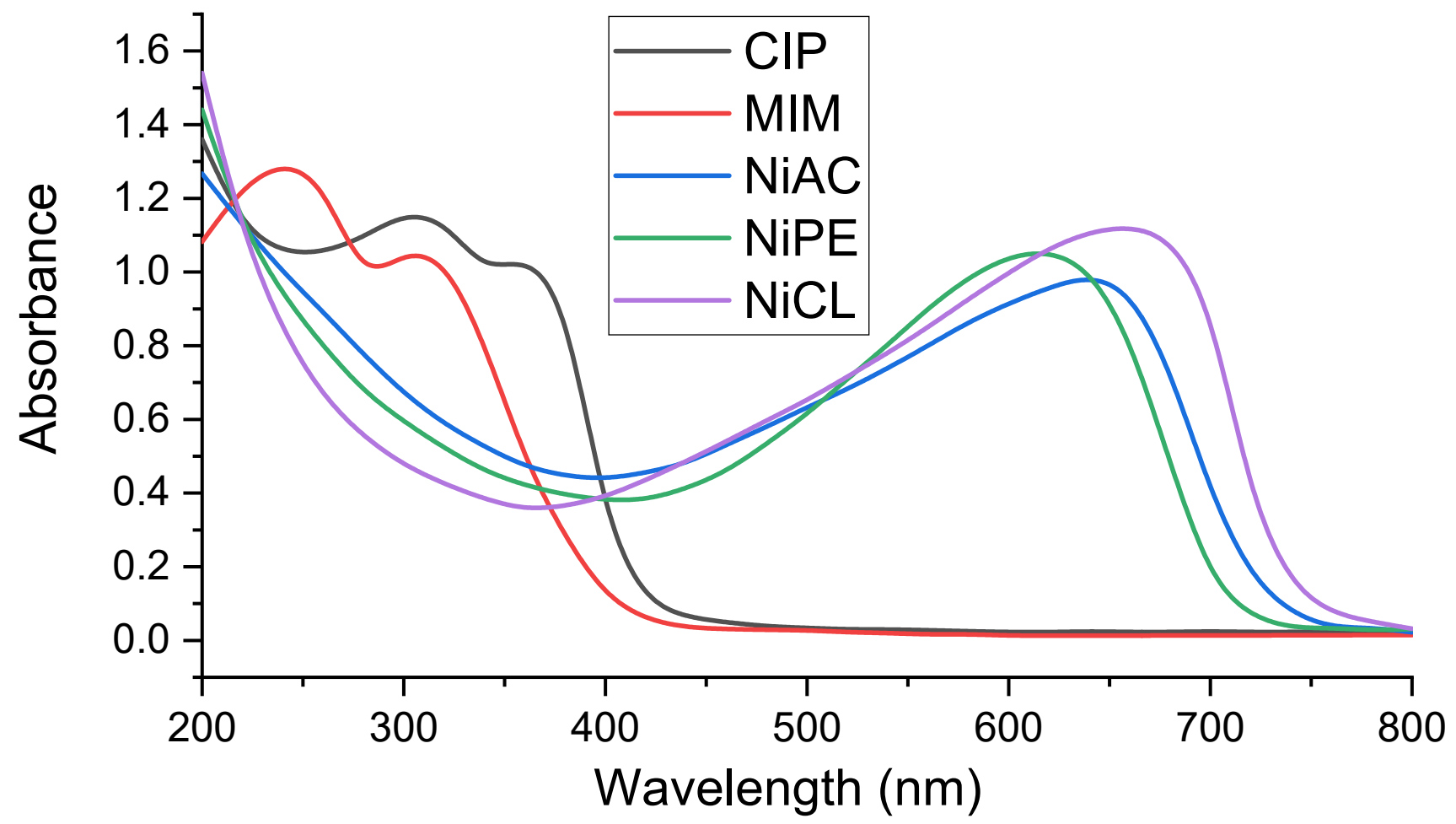

Fig. (S.2): The UV-vis. spectra of the of the NiAC, NiPE, and NiCL at 10^{-3} M.

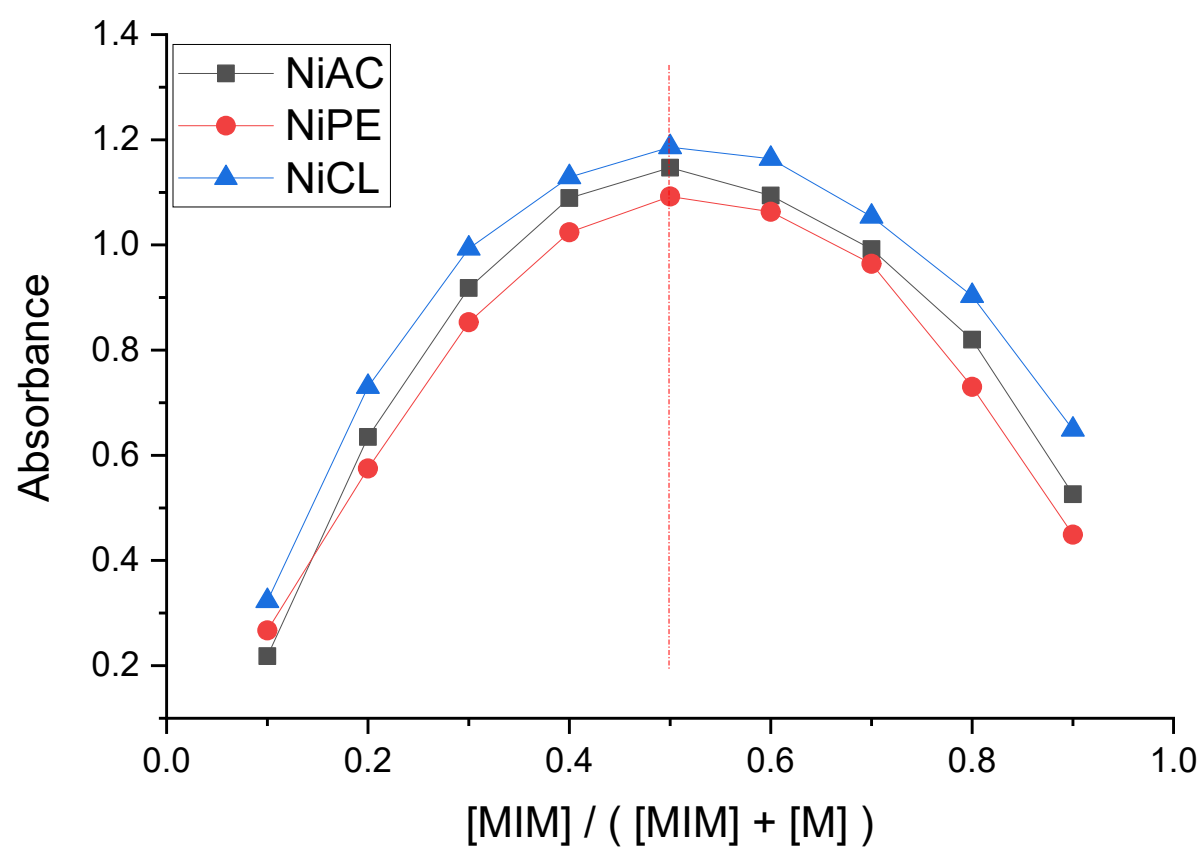

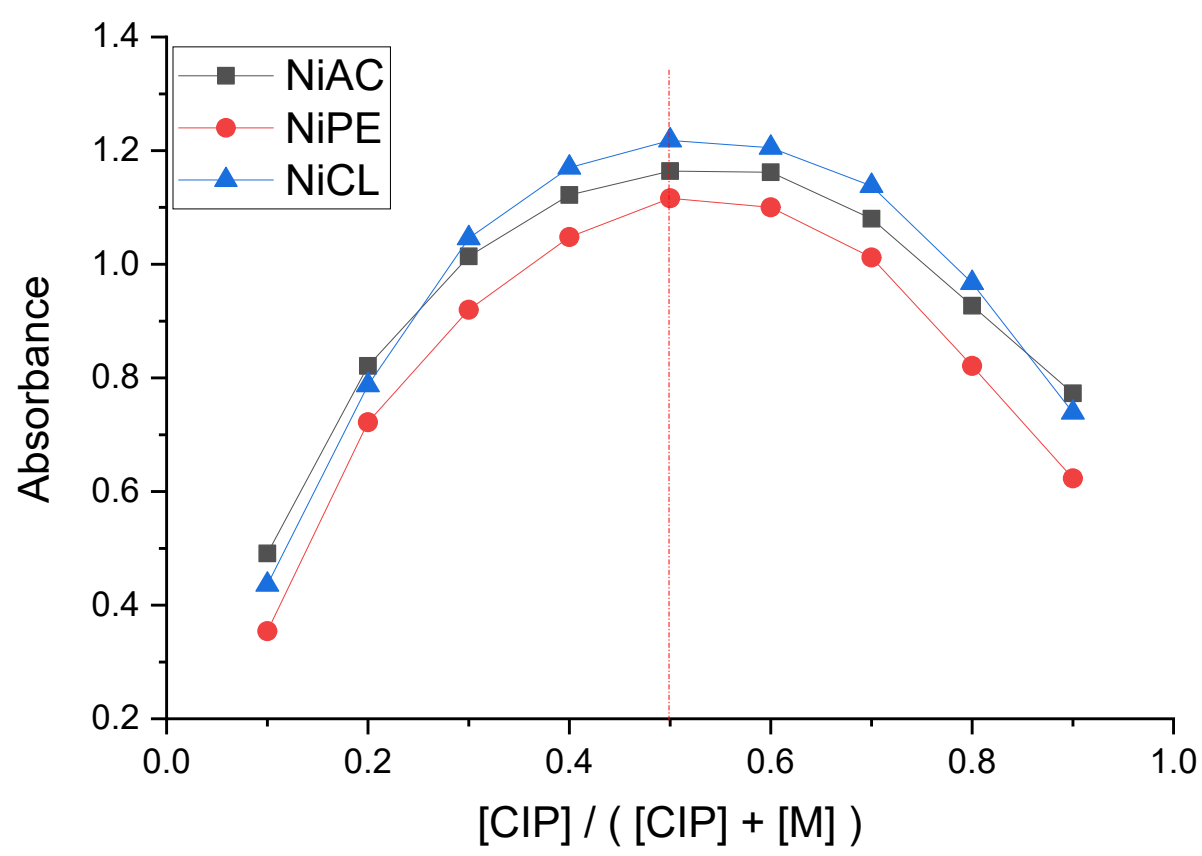

Fig. (S.3): The Stiochiometry of the NiAC, NiPE, and NiCL

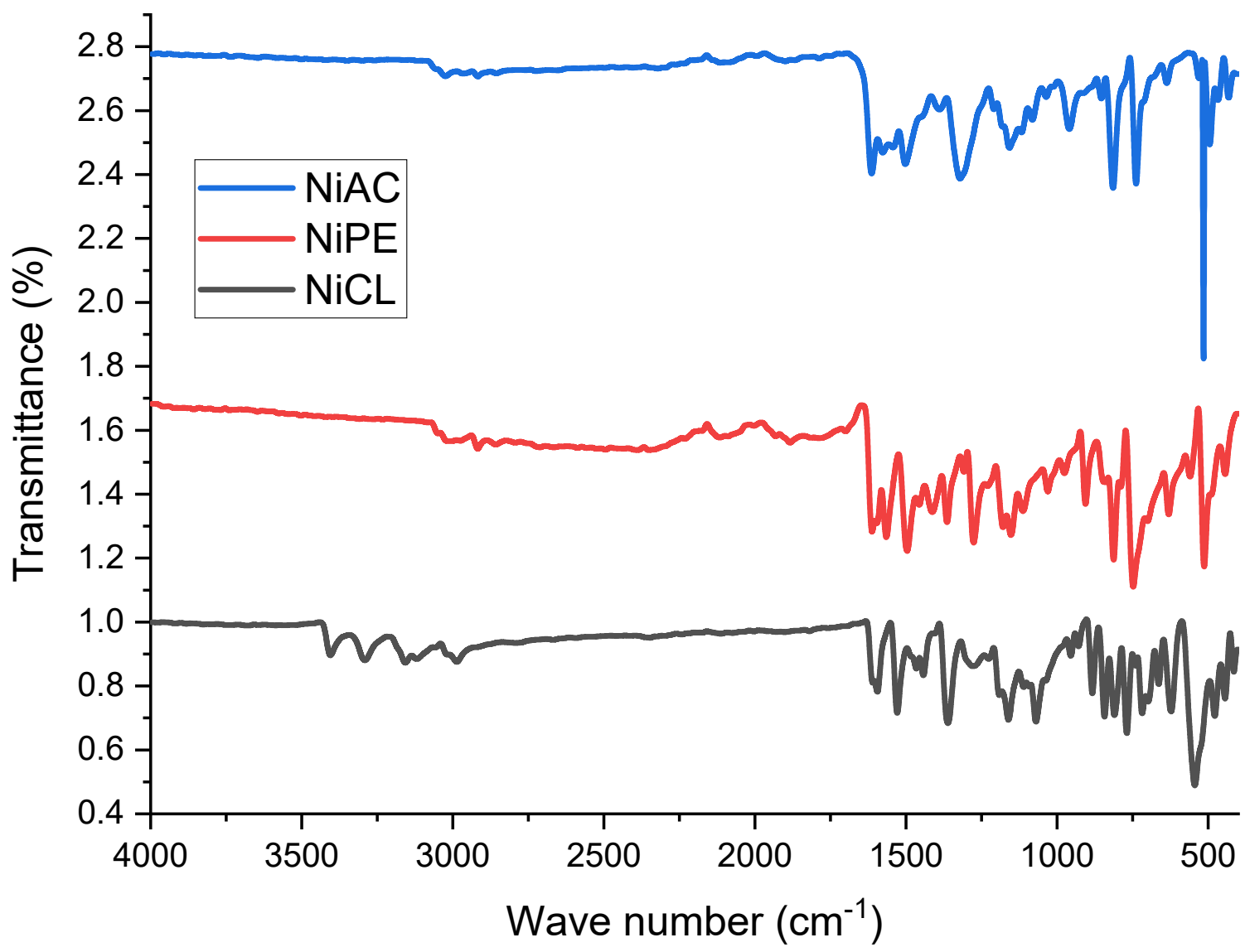

Fig. (S.4): The FT-IR. spectra of the of the NiAC, NiPE, and NiCL complexes

NiAC

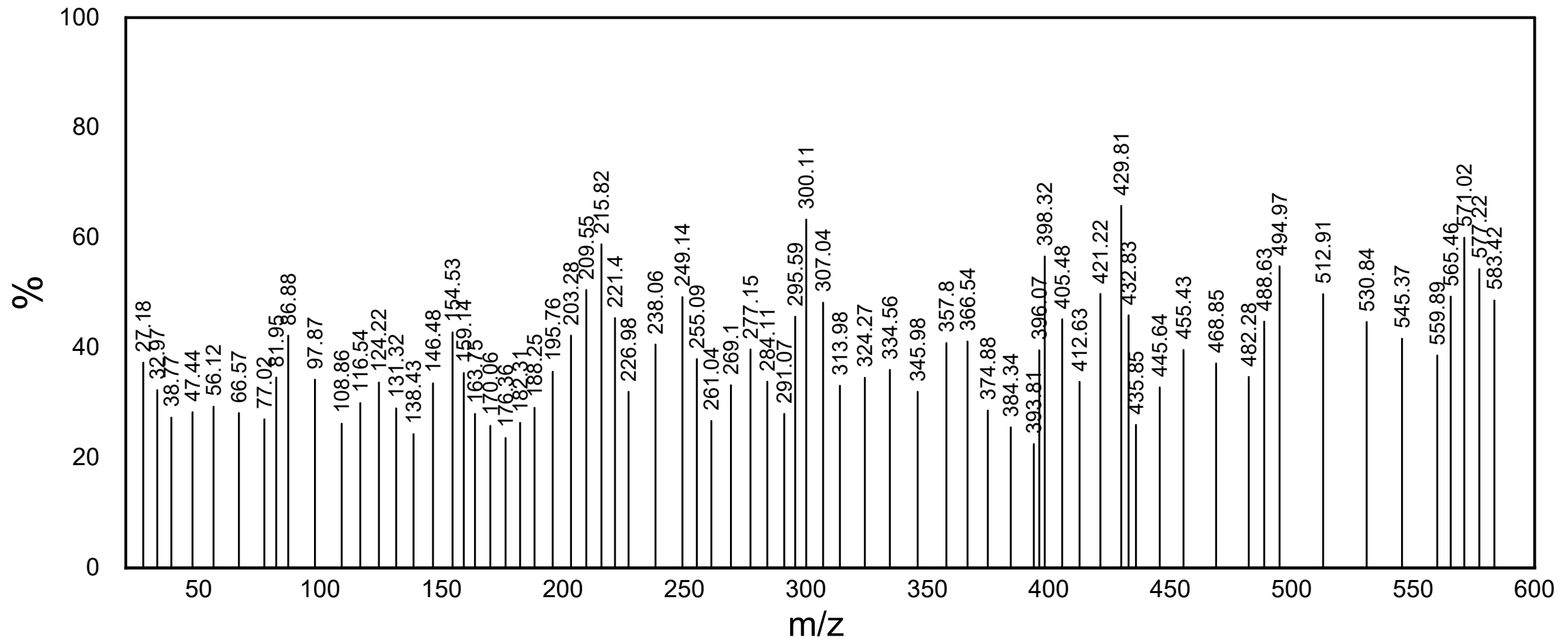

NiPE

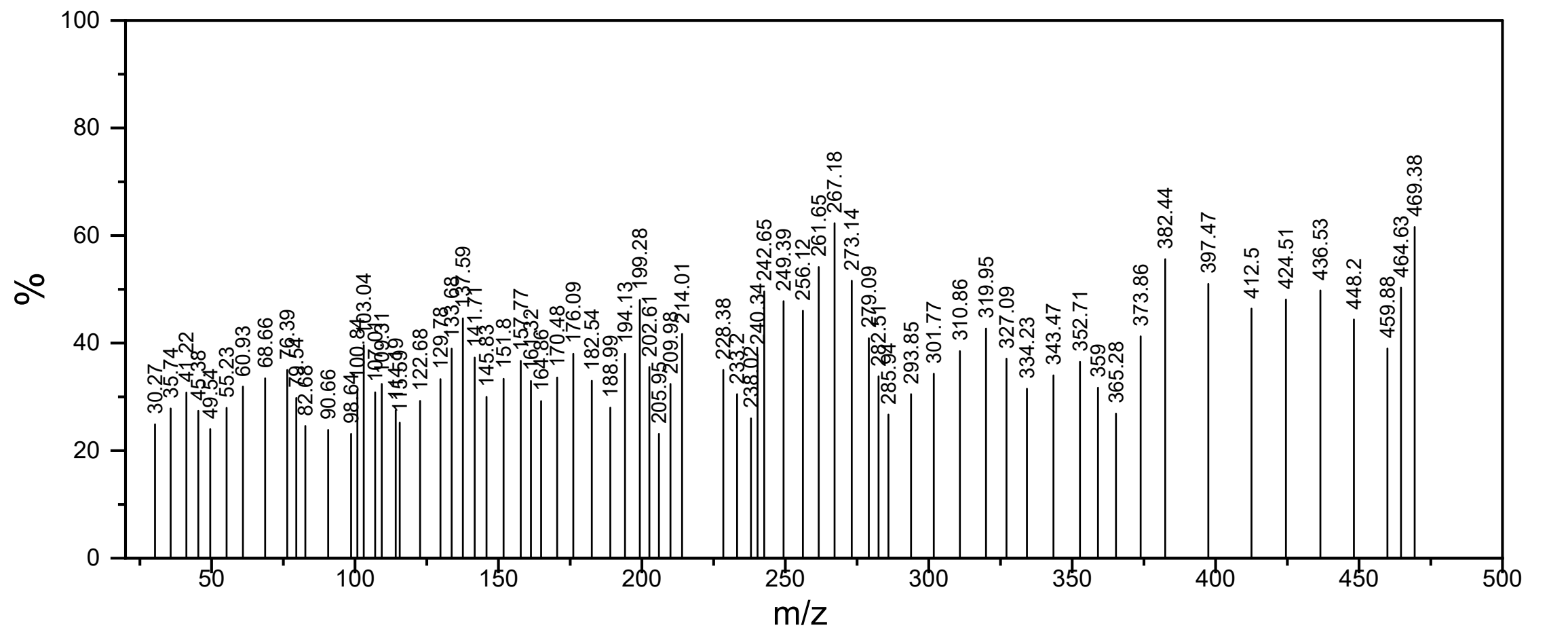

NiCL

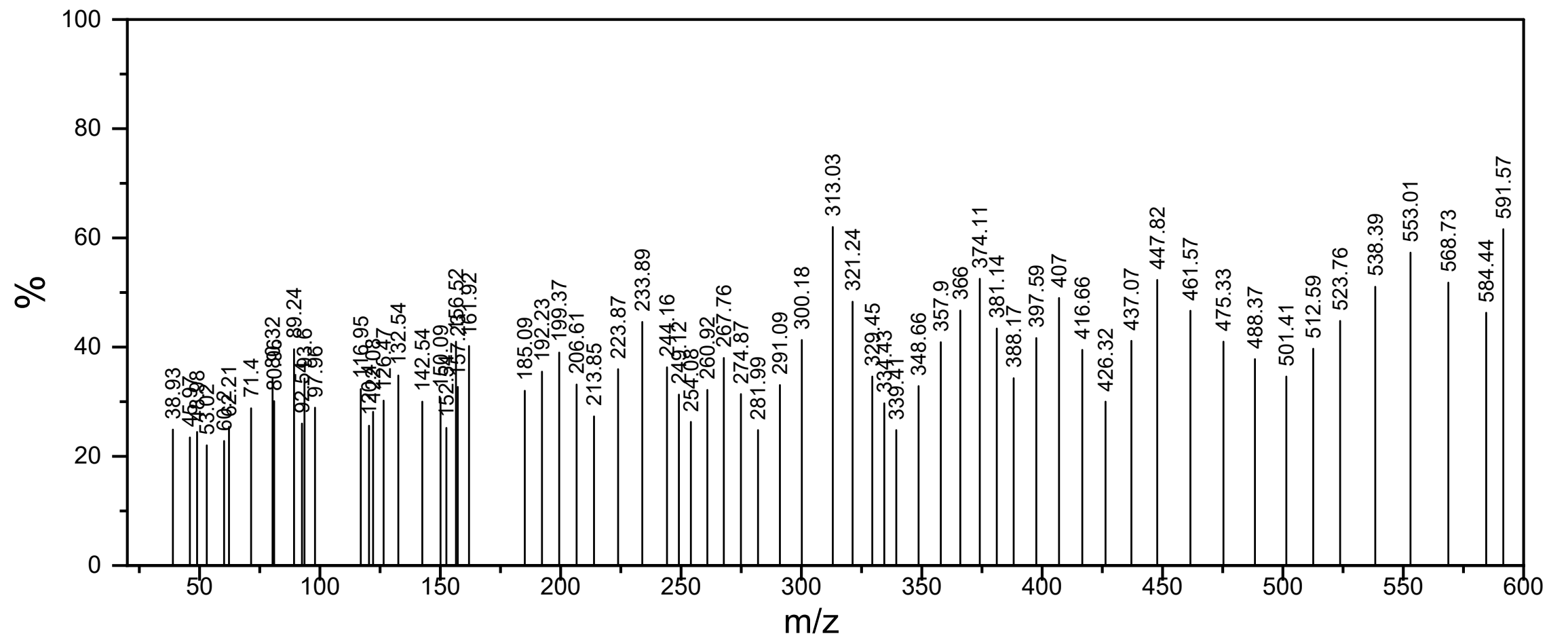

Fig. (S.5): Mass spectra of the of the NiAC, NiPE, and NiCL

NiAC

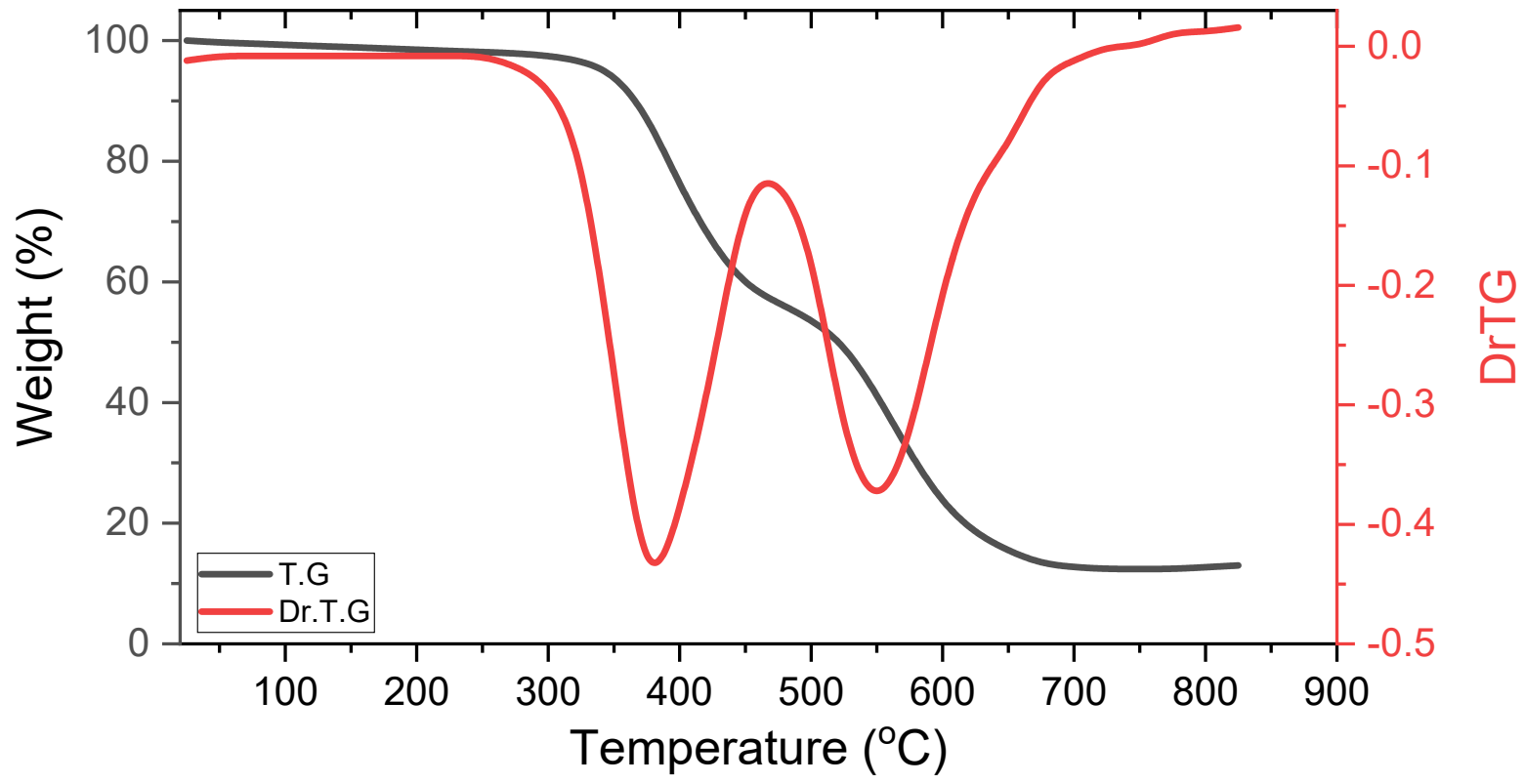

NiPE

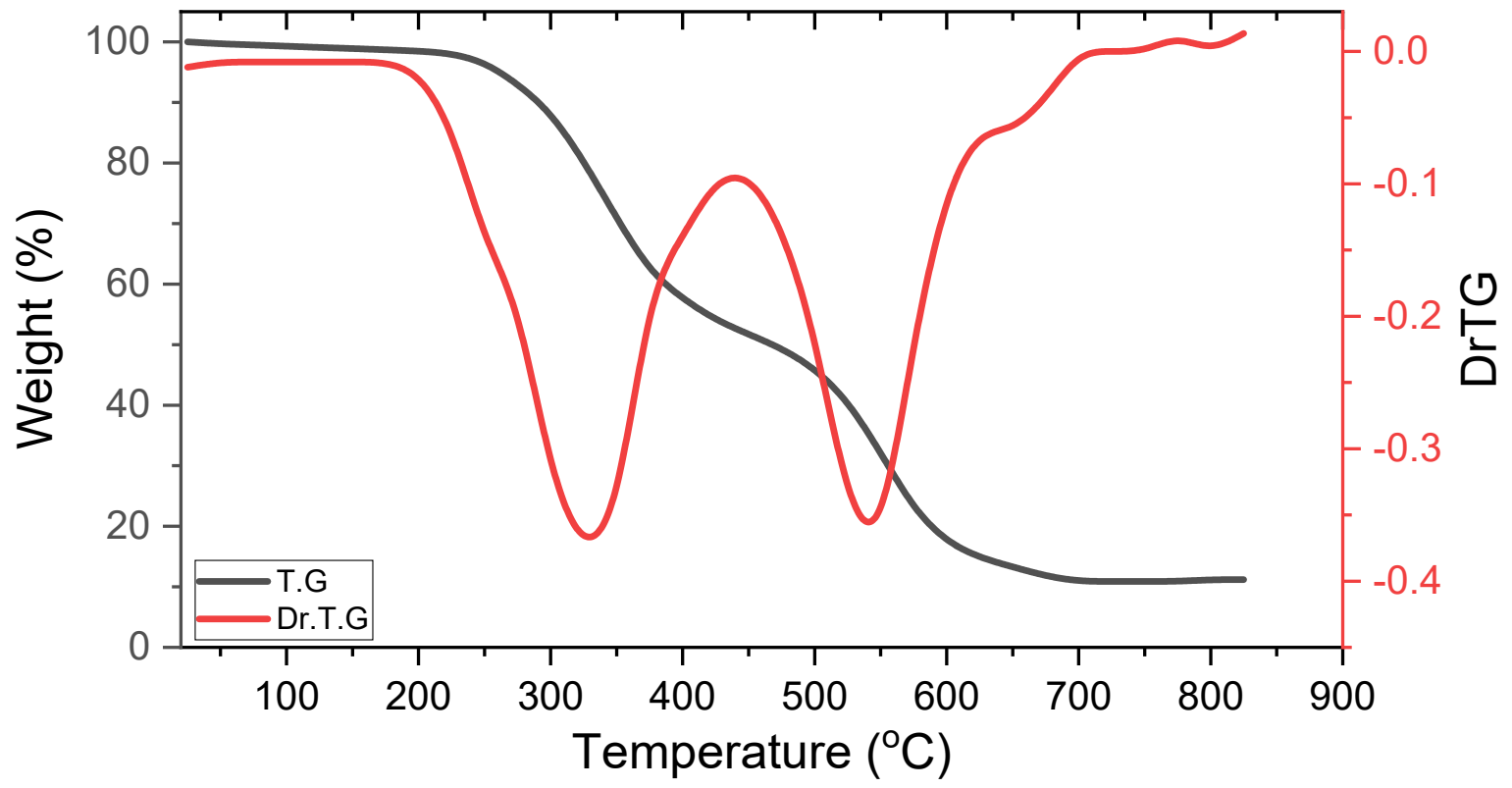

NiCL

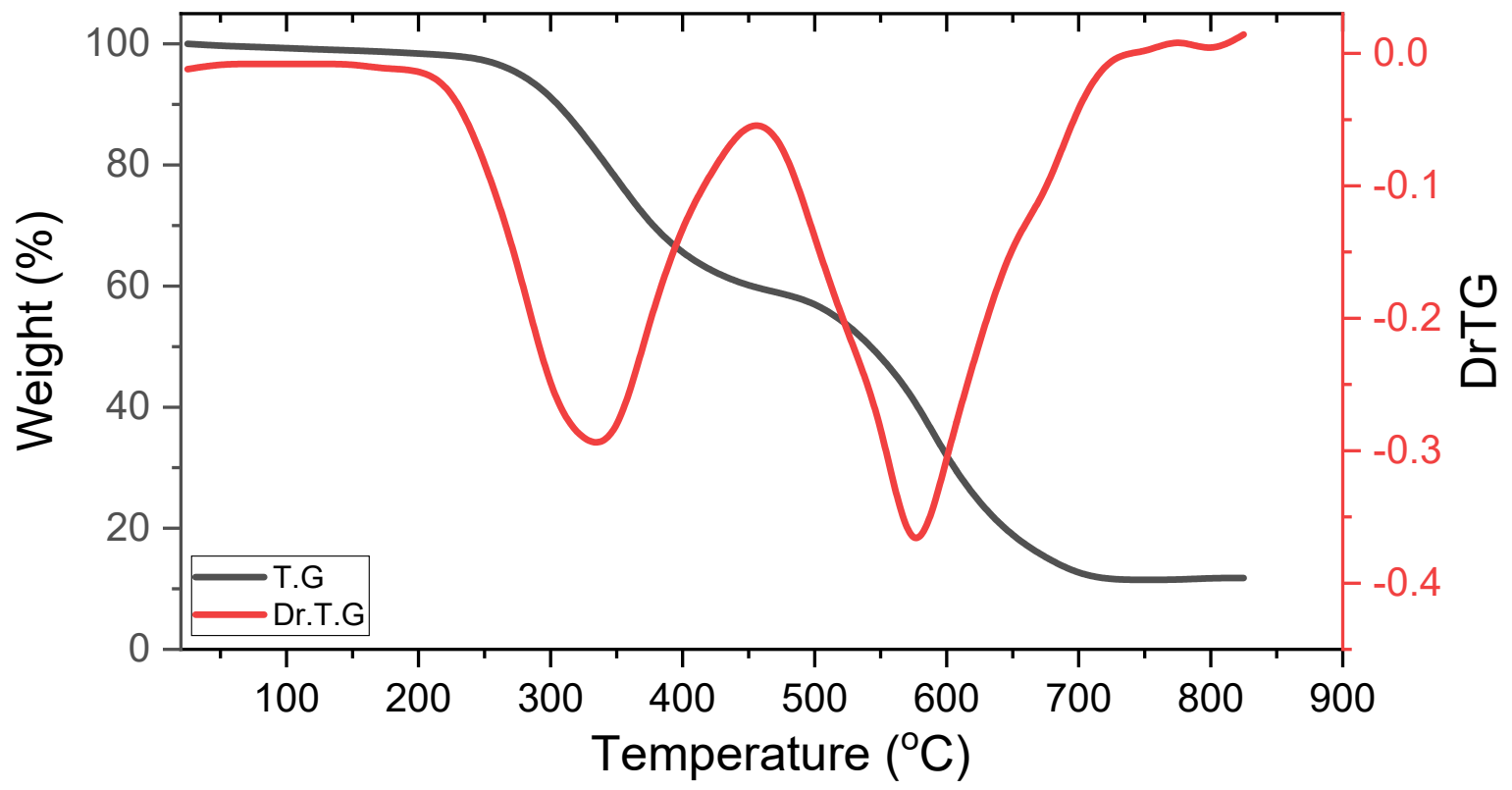

Fig. (S.6): Thermal degradation of the NiAC, NiPE, and NiCL complexes

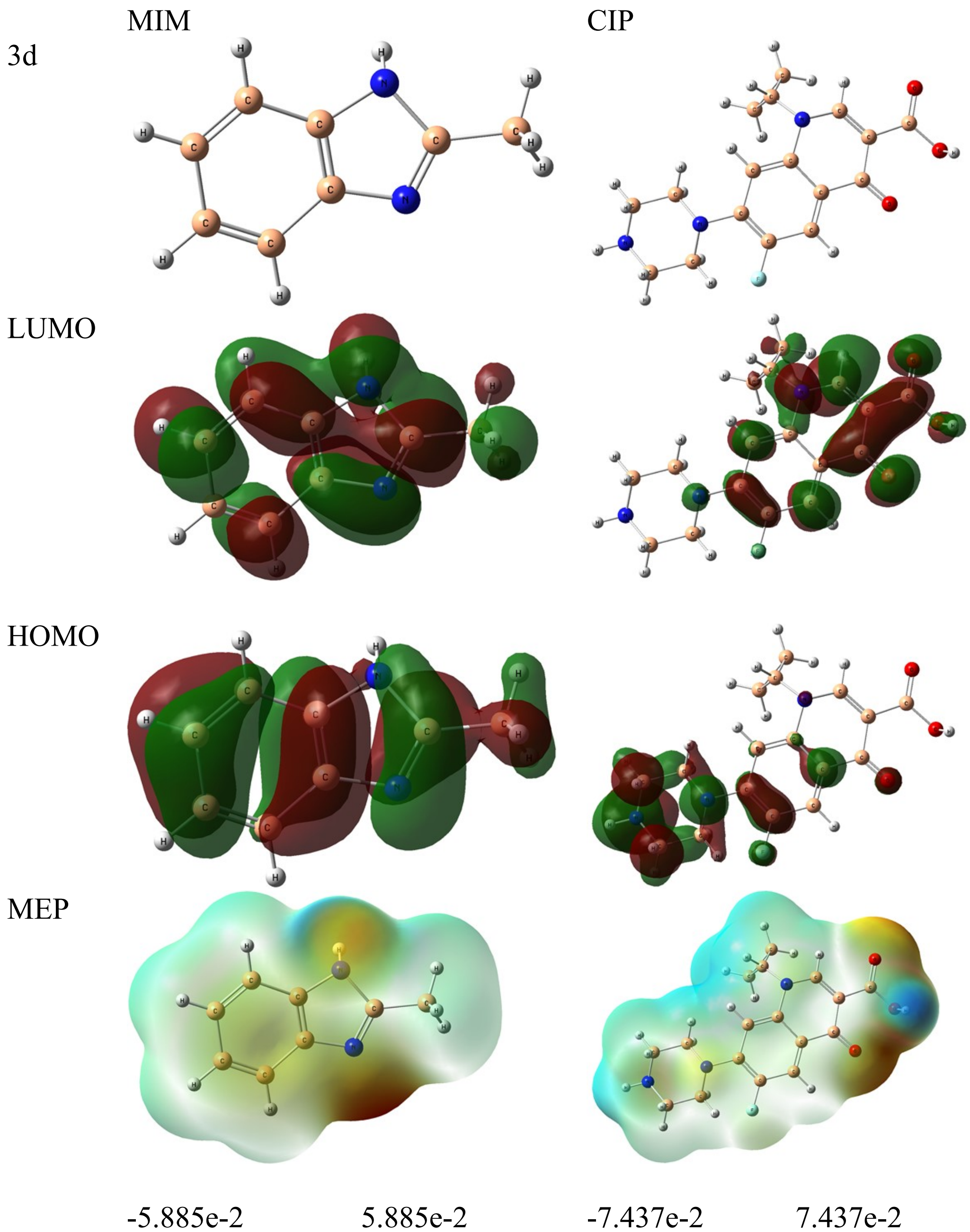

Fig. (S.7): 3D, HOMO, LUMO, and MEP of MIM, and CIP

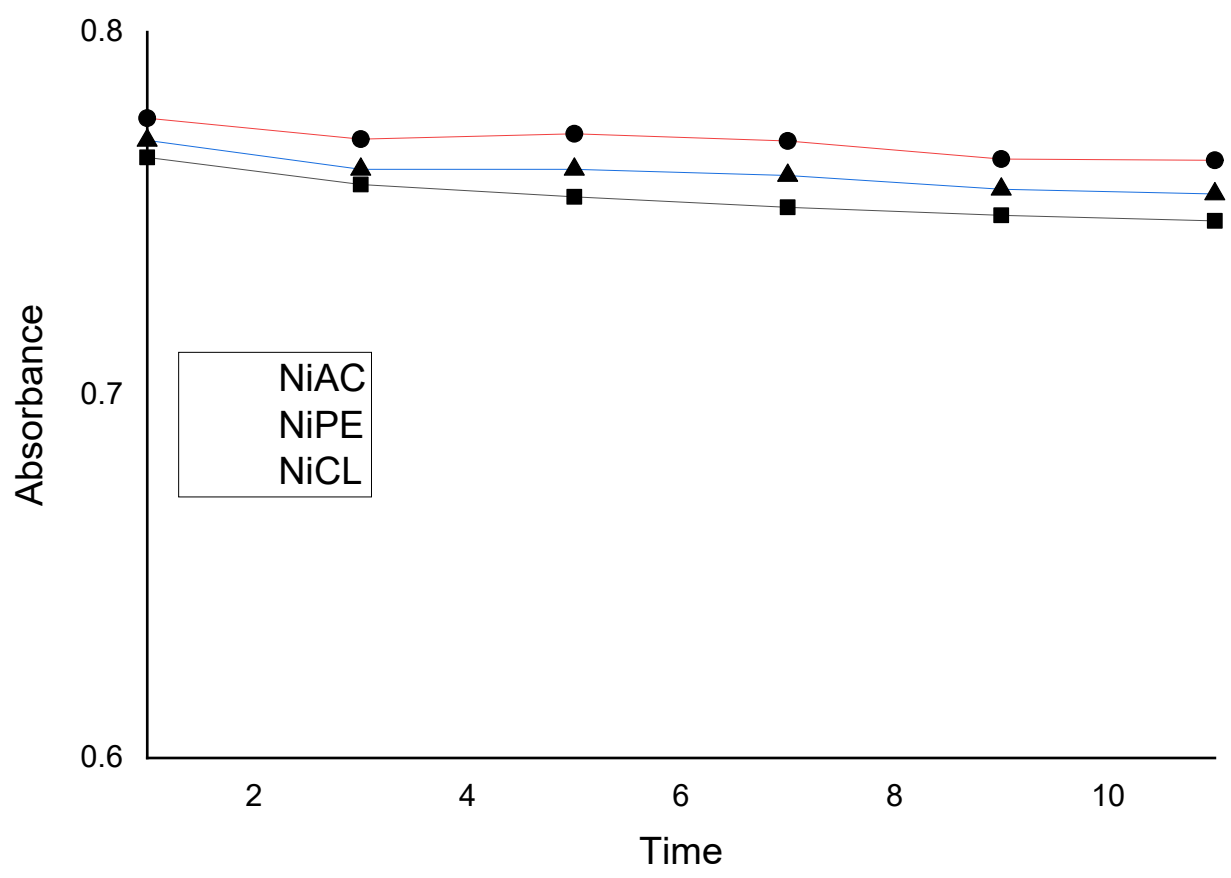

Fig. (S.8): stability curve of the NiAC, NiPE, and NiCL complexes in biologically relevant media containing DMSO, the absorbance was measured at the absorption maxima (λmax) for each Ni(II) complex (at 640 nm, 610 nm, and 675 nm for NIAC, NIPE and NICL complexes, respectively)

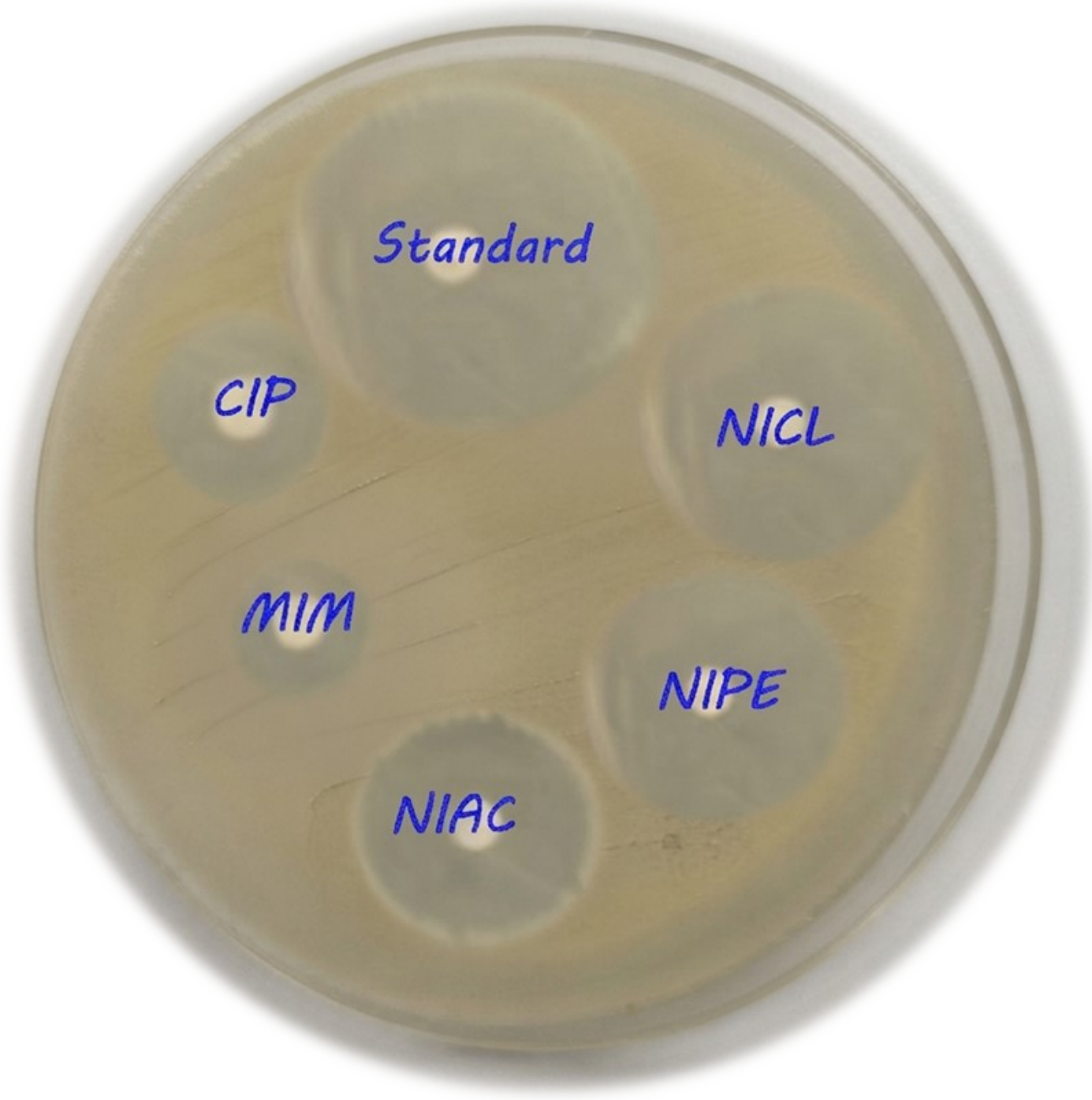

Fig. (S.9): The antibacterial activity as the inhibition zone photo in the case of *E. coli*.

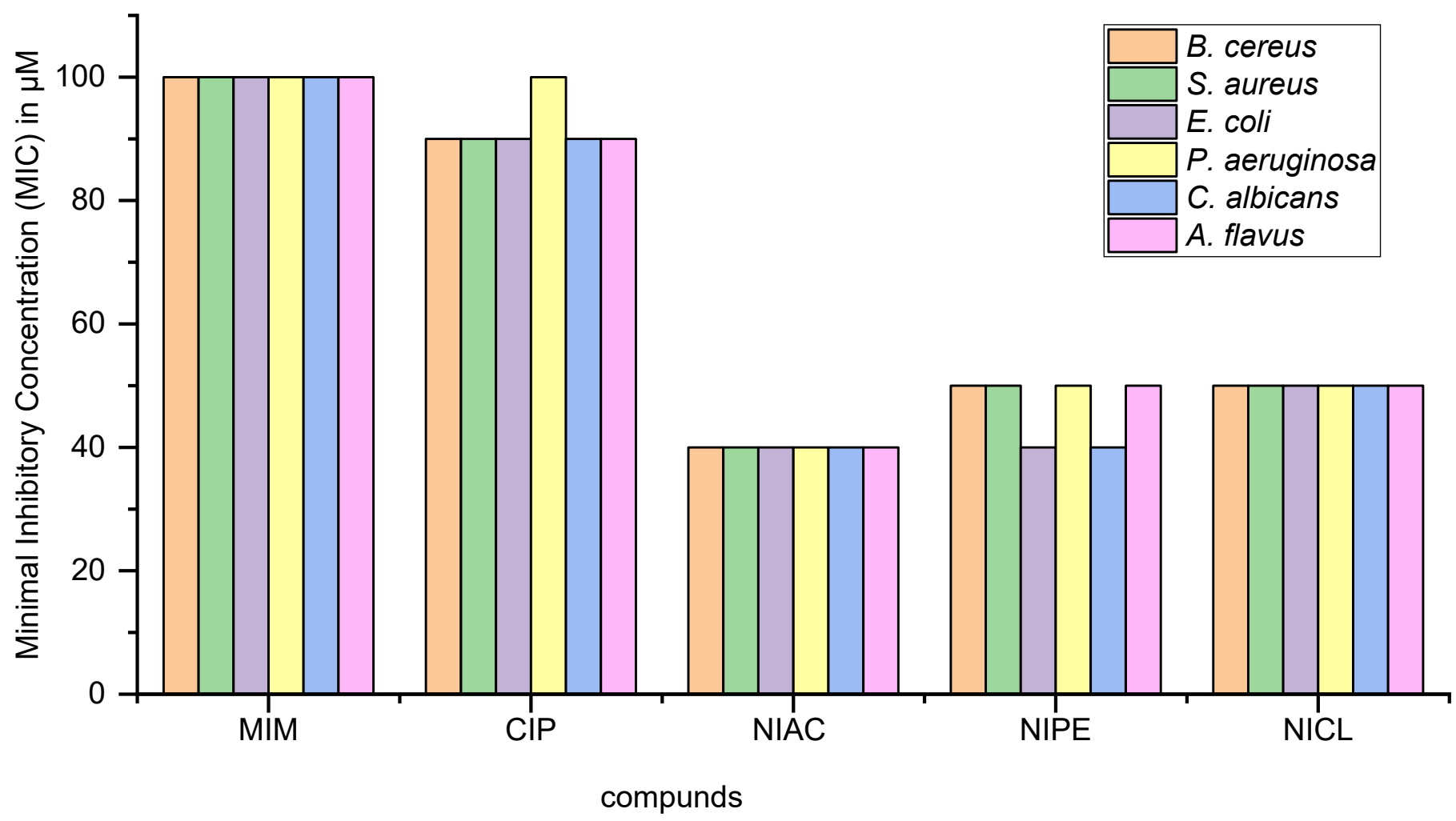

Fig. (S.10): Antibacterial and Antifungal activity as Minimum Inhibition concentration (MIC)

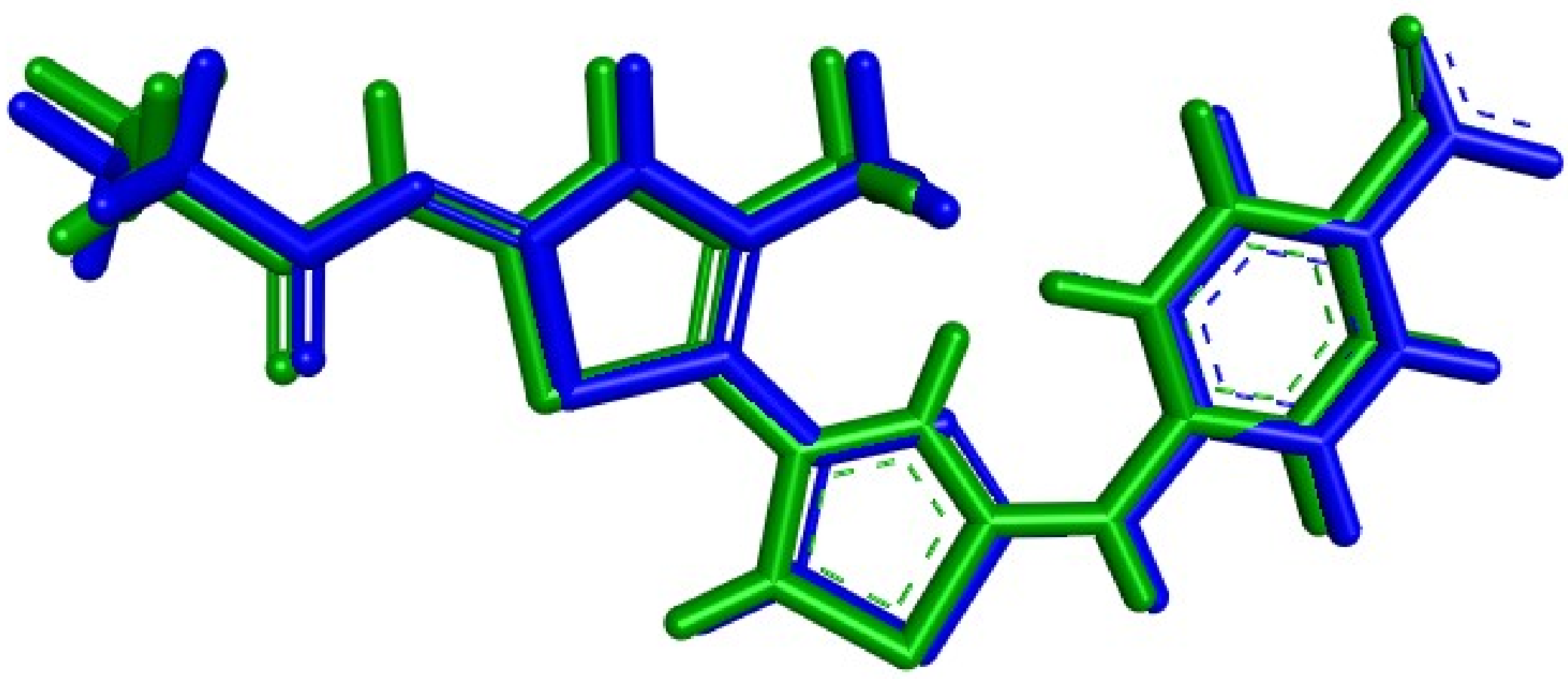

Fig. (S.11): 3D representation of the superimposition of the co-crystallized (blue) and the redocked (green) ligand of 4DUH

[1] G. Mariappan, R. Hazarika, F. Alam, R. Karki, U. Patangia, S. Nath, Synthesis and biological evaluation of 2-substituted benzimidazole derivatives, Arabian Journal of Chemistry 8(5) (2015) 715-719.

[2] S.I. Alaqeel, Synthetic approaches to benzimidazoles from o-phenylenediamine: A literature review, Journal of Saudi Chemical Society 21(2) (2017) 229-237.

[3] N.A. Elkanzi, A.M. Ali, M. Albqmi, A. Abdou, New benzimidazole-based Fe (III) and Cr (III) complexes: characterization, bioactivity screening, and theoretical implementations using DFT and molecular docking analysis, Applied Organometallic Chemistry 36(11) (2022) e6868.

[4] F. Neese, Software update: The ORCA program system—version 6.0, Wiley Interdisciplinary Reviews: Computational Molecular Science 15(2) (2025) e70019.

[5] A. Schäfer, C. Huber, R. Ahlrichs, Fully optimized contracted Gaussian basis sets of triple zeta valence quality for atoms Li to Kr, The Journal of chemical physics 100(8) (1994) 5829-5835.

[6] F. Weigend, R. Ahlrichs, Balanced basis sets of split valence, triple zeta valence and quadruple zeta valence quality for H to Rn: Design and assessment of accuracy, Phys. Chem. Chem. Phys. 7(18) (2005) 3297-3305.

[7] Mamta, Pinki, A. Chaudhary, Synthesis, spectroscopic elucidation, density functional theory calculation, and molecular docking studies of a novel series of tetradentate macrocyclic Schiff base ligands and their Zn (II) complexes and investigations of their antimicrobial, anti-inflammatory, and anticancer activities, Applied Organometallic Chemistry 38(2) (2024) e7330.

[8] M.D. Hanwell, D.E. Curtis, D.C. Lonie, T. Vandermeersch, E. Zurek, G.R. Hutchison, Avogadro: an advanced semantic chemical editor, visualization, and analysis platform, Journal of cheminformatics 4 (2012) 1-17.

[9] T. Lu, F. Chen, Multiwfn: A multifunctional wavefunction analyzer, Journal of computational chemistry 33(5) (2012) 580-592.

[10] M. Miar, A. Shiroudi, K. Pourshamsian, A.R. Oliaey, F. Hatamjafari, Theoretical investigations on the HOMO–LUMO gap and global reactivity descriptor studies, natural bond orbital, and nucleus-independent chemical shifts analyses of 3-phenylbenzo[d]thiazole-2(3H)-imine and its para-substituted derivatives: Solvent and substituent effects, Journal of Chemical Research 45(1-2) (2020) 147-158.

[11] V. Choudhary, A. Bhatt, D. Dash, N. Sharma, DFT calculations on molecular structures, HOMO–LUMO study, reactivity descriptors and spectral analyses of newly synthesized diorganotin(IV) 2-chloridophenylacetohydroxamate complexes, Journal of Computational Chemistry 40(27) (2019) 2354-2363.

[12] M.A.E.A.A.A. El-Remaily, O. Elhady, A. Abdou, D. Alhashmialameer, T.N.A. Eskander, A.M. Abu-Dief, Development of new 2-(Benzothiazol-2-ylimino)-2,3-dihydro-1H-imidazol-4-ol complexes as a robust catalysts for synthesis of thiazole 6-carbonitrile derivatives supported by DFT studies, Journal of Molecular Structure 1292 (2023) 136188.

[13] A.E. Saidi, N. Bouzidi, M. Ziane, M. Gherib, C. Rahila, M. Mioc, In silico and in vitro studies: investigating the chemical composition, DFT, molecular docking, and dynamic simulation of Satureja candidissima (Munby) Briq essential oil as a potential antibacterial agent, Journal of Biomolecular Structure and Dynamics 43(8) (2025) 4043-4062.

[14] M. Mackeen, A. Ali, S. El-Sharkawy, M. Manap, K. Salleh, N. Lajis, K. Kawazu, Antimicrobial and cytotoxic properties of some Malaysian traditional vegetables (ulam), Int. J. Pharm. 35(3) (1997) 174-178.

[15] N. Raman, A. Sakthivel, K. Rajasekaran, Synthesis and spectral characterization of antifungal sensitive Schiff base transition metal complexes, Mycobiology 35(3) (2007) 150-153.

[16] A.D. Kulkarni, S.A. Patil, P.S. Badami, Electrochemical properties of some transition metal complexes: synthesis, characterization and in-vitro antimicrobial studies of Co (II), Ni (II), Cu (II), Mn (II) and Fe (III) complexes, International Journal of Electrochemical Science 4(5) (2009) 717-729.

[17] I. Wiegand, K. Hilpert, R.E. Hancock, Agar and broth dilution methods to determine the minimal inhibitory concentration (MIC) of antimicrobial substances, Nat. Protoc. 3(2) (2008) 163-175.

[18] A. Gahlan, M. El-Mottaleb, N. Badawy, F. Kamale, S.H. Ali, Spectrophotometeric studies on binary and ternary complexes of some metal ions with alizarin red S and cysteine, Int. J. Adv. Res 2(10) (2014) 570-584.

[19] R.M. El-Khatib, L.A.-M.E. Nassr, Spectrophotometric study of some Mn (II) ternary complexes and their analytical applications, Monatshefte für Chemie-Chemical Monthly 140 (2009) 1139-1142.

[20] H. Temel, Ü. Çakir, H.İ. Uğraş, M. Şekerci, The synthesis, characterization and conductance studies of new Cu (II), Ni (II) and Zn (II) complexes with the Schiff base derived from 1, 2-bis-(o-aminophenoxy) ethane and salicylaldehyde, Journal of Coordination Chemistry 56(11) (2003) 943-951.
